# Neurophysiological and Behavioral Effects of Micro- and Nanoplastics in Aquatic Organisms

**DOI:** 10.3390/ani16060941

**Published:** 2026-03-17

**Authors:** Rachelle M. Belanger, Levi Storks

**Affiliations:** Biology Department, University of Detroit Mercy, 4001 W. McNichols Rd., Detroit, MI 48221, USA; storksle@udmercy.edu

**Keywords:** microplastics, nanoplastics, aquatic toxicity, neurotoxicity, behavioral toxicity, molecular toxicity

## Abstract

Industrialization has caused extensive environmental change, including a global surge in plastic production and pollution. This has resulted in the accumulation of microplastics (MPs; <5 mm) and nanoplastics (NPs; <1 μm) in ecosystems worldwide. MPs originate from both primary sources, such as cosmetics and industrial applications, and secondary sources, through the degradation of larger plastic debris. As a result, MPs and NPs have become ubiquitous contaminants, posing significant toxicological risks to living organisms. These persistent pollutants are diverse polymers that vary in size, shape, and chemical composition, making their impacts on organism physiology complex and difficult to disentangle. Plastic pollution is particularly severe in aquatic environments, where particles accumulate from terrestrial sources such as urban dust, agricultural runoff, industrial discharges, and wastewater effluents. Although most research has centered on marine ecosystems, emerging evidence indicates that freshwater environments may contain comparable or even higher concentrations of MPs. Once inside the body, MPs can translocate into tissues and exert toxic effects on multiple organ systems. Collectively, plastic pollution poses not only physiological but also neurological and behavioral risks to aquatic life, with potential consequences for ecosystem stability and trophic interactions. Both MPs and NPs are sufficiently small to cross the blood–brain barrier, raising concerns about their potential impacts on the nervous system by interfering with neuronal function and brain development. Plastic particles can accumulate in neural tissues, inducing oxidative stress, neuroinflammation, and disruption of neurotransmitter signaling. Such neurotoxic effects are linked to altered locomotion, feeding, predator avoidance, and social behaviors across multiple species. This review examines current evidence on the neurotoxic effects of plastic pollution in aquatic organisms and underscores the urgent need for further research and action to mitigate its impact. In light of escalating plastic production and inadequate waste management, the growing evidence that MPs and NPs disrupt aquatic nervous systems, behavior, and ecosystem stability underscores an urgent need for intensified research, improved mitigation strategies, particularly for nanoplastics, and the accelerated development of truly safe and sustainable alternatives.

## Simple Summary

The widespread use of plastics has led to growing pollution in the environment, especially in the aquatic ecosystem. Tiny plastic pieces called microplastics and even smaller nanoplastics now contaminate rivers, lakes, and oceans around the world. These particles come from products like cosmetics, clothing fibers, and the breakdown of larger plastic waste. Freshwater environments may contain as much, or even more, plastic pollution than oceans. Because these plastic particles are so small, animals can accumulate them in their bodies. Once inside, the particles can move into organs, including the brain. Research shows that microplastics and nanoplastics can harm aquatic animals by causing stress and inflammation in brain and nervous tissue, interfering with normal nerve signaling. These effects may change how animals move, eat, avoid predators, and interact with each other. Such behavioral and neurological changes can disrupt food webs and threaten the health of entire ecosystems. This review summarizes current research on how plastic pollution affects the nervous systems of aquatic organisms and highlights the urgent need for more research and efforts to reduce plastic pollution in the environment.

## 1. Introduction

Human activities during the Anthropocene have profoundly reshaped the Earth, with plastic contamination standing out as a major driver of anthropogenic change [1,2,3]. Since the 1950s, a rapid global increase in plastic production has resulted in unprecedented amounts of plastic waste entering the environment through a wide range of human activities. Globally, plastic production has risen from approximately 2 million tonnes in the 1950s to 359 million tonnes in 2018, with China, Europe, and North America accounting for the largest shares of plastic manufacturing [4,5]. A substantial portion of this waste occurs as microplastics (MPs), defined as plastic particles less than 5 mm in diameter [6,7,8,9]. MPs originate from both primary and secondary sources, where primary MPs are intentionally manufactured for use in cosmetics, pharmaceuticals, detergents, and industrial plastic production; and secondary sources, where secondary MPs form through the degradation of larger plastic items [6,7,10]. MPs can break down into smaller fragments through physical, chemical, and biological processes into particles called nanoplastics (<1 μm; NPs) [8,9,11]. Diversity in polymer type, size, and shape makes MPs a highly complex pollutant that can persist for millennia [7,10,12,13]. MPs have permeated ecosystems globally where they pose widespread toxicological risks to organisms [6,14].

Widespread MP contamination in aquatic ecosystems has emerged as a significant global ecological and scientific concern, as they serve as primary sinks for plastic debris and provide continuous exposure pathways for a wide range of organisms [15]. MP pollution is severe in aquatic environments, where MPs accumulate from terrestrial inputs, including city dust, agricultural and urban runoff, industrial activity, landfill leachate, or wastewater effluents, resulting in persistent and often bioavailable particulate contamination [7,10,16,17]. Aeolian transportation and precipitation events have also been shown to be a major contributor to primary and secondary plastics in aquatic environments [18,19,20]. Aquatic organisms readily uptake and internalize MPs and NPs across trophic levels, allowing these particles to interact directly with sensitive tissues, including the nervous system [21,22,23]. Such plastic particles enter the body of aquatic organisms through direct and indirect ingestion, gill uptake, and transdermal exposure and can translocate across biological barriers into the nervous system, where they can induce oxidative stress (OS) and inflammation [23,24,25,26,27,28]. Disruption of neural function may manifest as altered behavior, impaired sensory processing, and reduced predator avoidance or foraging efficiency, outcomes that have direct consequences for survival and ecosystem services these species provide [21,23,29,30,31,32,33]. Despite growing evidence of behavioral alterations, physiological dysfunction, and neurological changes in aquatic species, these MP- and NP-induced endpoints remain less systematically synthesized. This review synthesizes current knowledge on (1) the occurrence and characteristics of MPs and NPs in aquatic environments, (2) their uptake, translocation, and neurotoxic mechanisms in aquatic organisms, and (3) the resulting physiological, neurological, genetic, and behavioral consequences, with implications for ecosystem stability and future research priorities. By integrating these findings, the review highlights critical knowledge gaps and underscores the urgent need for effective strategies to mitigate plastic pollution in aquatic ecosystems. Although previous reviews have addressed MP toxicity broadly and provided foundational insights into neurotoxic mechanisms, comprehensive integration of recent evidence across cellular, neurochemical, genetic, and behavioral endpoints remains limited. Since 2019, the literature has expanded substantially, particularly in relation to NPs, environmentally aged particles, co-exposure scenarios, and transgenerational effects. This review synthesizes these emerging findings across aquatic taxa and biological scales, providing an updated and cross-level perspective on the neurobehavioral risks of plastic pollution in aquatic ecosystems.

### 1.1. Sources, Characteristics, and Environmental Distribution of MPs and NPs

#### 1.1.1. Sources of MPs and NPs

Plastics are persistent pollutants that exist in many forms and continue to accumulate ubiquitously in the Earth’s waterways, posing potential adverse effects on aquatic ecosystems [34]. Primary MPs are intentionally manufactured as tiny particles used in a range of products (e.g., cosmetic microbeads, soaps, paints, abrasive products, pharmaceuticals, or pellets). These particles can bypass wastewater treatment systems due to their small size and are readily discharged into aquatic environments. Secondary plastics form from unintentional breakdown of larger plastic items (e.g., bottles, packaging, fishing nets, bags, tires, etc.) [35,36,37,38,39]. Environmental stressors, including thermal, thermo-oxidative, photo-oxidative, biological processes, UV radiation, and mechanical forces such as tire abrasion, drive the degradation of plastics [35]. Over time, this breakdown produces a continuum of particle sizes, including MPs and NPs that exhibit enhanced mobility and bioavailability [40]. Both primary and secondary sources contribute substantially to the widespread and persistent presence of MPs and NPs in aquatic ecosystems, underscoring the need for source-specific mitigation strategies and improved waste management practices.

Plastics enter aquatic ecosystems through multiple interconnected pathways originating from land- and sea-based human activities. MPs and NPs are found in virtually all aquatic ecosystems on the planet, from the deep sea to estuaries, freshwater rivers and lakes [41]. They accumulate from terrestrial inputs, aeolian transport, and precipitation events [7,10,16,17,18,19,20,42]. Terrestrial inputs include city dust, stormwater runoff, wastewater effluents, landfill leachate, and industrial activities. Wastewater treatment plants are significant sources of MPs, as fibers from synthetic textiles and particles from personal care products are incompletely removed during treatment and subsequently released into receiving waters, making urban areas a large contributor of discharged MPs [43,44]. Some treatment plants that use skimming and settling treatment processes can significantly decrease the amount of plastic discharge from wastewater treatment plants [43,45]. Rivers are the major conduit for plastics entering lakes and oceans, resulting in persistent and often bioavailable particulate contamination [46].

Global plastic production reached approximately 367 million tonnes in 2020, up from just 2 million tonnes in 1950 [47]. This number is expected to double within the next 20 years, driven by continued global reliance on plastics across packaging, consumer goods, medical applications, and industrial sectors [46]. Ongoing global dependence on plastics is expected to increase plastic discharge into aquatic environments, as approximately 10% of annual production enters these systems as debris that subsequently degrades into MP and NP particles [48]. Although plastic pollution has received increasing attention in recent years, available evidence suggests the problem is escalating [49]. The pervasive accumulation and widespread distribution of MP and NP particles in aquatic environments make exposure of aquatic organisms unavoidable [23].

#### 1.1.2. Polymer Types, Sizes, Shapes, and Chemical Additives

Plastics in aquatic environments exhibit substantial heterogeneity in polymer composition, size, shape, and chemical complexity, all of which influence their environmental fate and biological interactions. Plastics are synthetic organic polymers generated through addition or condensation of hydrocarbon monomers, characterized by their chemical stability, lipophilicity, and water resistance [21]. Items such as single-use plastic beverage, food, and consumer product packaging are inexpensive to produce, have short lifespans, and are readily discarded into the environment, where they fragment into progressively smaller particles. In addition to primary MPs, secondary MPs can also be classified based on physical and chemical defining properties [37]. These properties influence their environmental behavior, persistence, and interactions with organisms. MPs are characterized by physical properties such as size, shape, density, surface roughness, and color, as well as chemical properties including polymer composition, hydrophobicity, chemical stability, additives, and pollutant sorption [39,40,50]. Casagrade et al. [50] suggests that size, shape, and the polymer type of MPs are the most important physical properties with respect to ecotoxicology; however, laboratory studies on the effects of plastics use regularly shaped MP and NP particles of one polymer type, while the majority of plastics found in the environment are irregularly shaped (fibers and fragments) and composed of a variety of polymers [6,50]. The polymer types most frequently detected in aquatic environments include low- and high-density polyethylene (LDPE and HDPE), polyethylene (PE), polypropylene (PP), polystyrene (PS), polyvinyl chloride (PVC), and polyethylene terephthalate (PET), reflecting their widespread use, diverse densities, and resistance to degradation [8,39,50,51].

Plastic polymers come in a variety of sizes and shapes, including fibers, fragments, films, foams, and pellets, which differentially affect transport, bioavailability, and organismal uptake [8,21,50,51,52]. Plastics also contain numerous chemical additives, including plasticizers, flame retardants, stabilizers, pigments, and antioxidants, many of which can leach into surrounding waters during environmental weathering [53,54]. In addition, plastic particles readily sorb hydrophobic organic contaminants and metals, such as polycyclic aromatic hydrocarbons, pesticides, and pharmaceuticals, allowing plastics to act as vectors for the transport and bioavailability of co-occurring pollutants [55,56,57]. Sorbed contaminants may desorb within aquatic organisms, though the mechanisms governing this process are not well characterized [18,58]. Together, the diversity of polymer types, variation in particle size and shape, and the presence of chemical additives and sorbed pollutants collectively drive the complex and heterogeneous toxicological profiles of plastics in aquatic ecosystems.

#### 1.1.3. Environmental Fate and Transport

Plastics and their degradation products are transported to aquatic environments through many pathways mentioned previously. As a result of these transport pathways, land-based sources dominate MP and NP inputs to aquatic environments, while the slow degradation of plastics drives their ongoing accumulation in marine, freshwater, and terrestrial ecosystems [6,7,10,15,49]. Riverine discharge is widely recognized as one of the dominant pathways for plastic transport into freshwater and marine environments, as approximately 80% of the global population resides within river basins, contributing an estimated 0.45 million tonnes of plastic released annually worldwide [59]. Rivers receive mobilized plastics from urban and agricultural runoff, wastewater treatment plant effluents, and mismanaged wastes [7,10,16,46]. Rain events lead to an increase in MP particles in river water due to a higher suspension and lower sedimentation concentrations. Additionally, increased rainfall events elevate water velocity, thereby extending transport distances [60]. Physical forces (e.g., winds and currents) and atmospheric deposition further facilitate long-range transport of plastics, allowing MPs and NPs to be redistributed across watersheds and deposited into remote freshwater and marine ecosystems [61,62,63]. Collectively, weather- and wind-driven processes play a critical role in shaping the distribution and concentration of MPs and NPs in aquatic environments, underscoring important differences between freshwater and marine systems.

Research on MPs has traditionally emphasized marine environments; however, freshwater systems are increasingly recognized as harboring comparable or, in some cases, higher concentrations of MPs [16,64,65,66,67,68]. There are similarities between marine and freshwater systems with respect to MP transportation, prevalence of MPs, approaches used for detection, identification, and quantification, and potential impacts [61]. Patterns of plastic contamination differ between freshwater and marine environments due to different salinity, hydrodynamics, residence times, and physical-chemical conditions [61,66]. Freshwater systems often exhibit highly variable MP and NP concentrations influenced by proximity to population centers, wastewater inputs, and seasonal flow dynamics, with rivers functioning both as sinks and conduits for plastic transport [19,61,66,68]. By contrast, marine systems are defined by extended residence times, large-scale circulation dynamics, and accumulation zones, including oceanic gyres and coastal sediments, which promote the broad dispersal and prolonged persistence of MPs and NPs [8,14,38,48,69]. Across freshwater and marine systems, environmental factors such as UV radiation, temperature, salinity, microbial activity, and mechanical abrasion influence the degradation, fragmentation, and persistence of plastic particles. These factors not only regulate the rate at which larger plastics break down into MPs and NPs but also affect particle density, surface properties, and biofouling, ultimately shaping transport pathways, bioavailability, and ecological risk in aquatic ecosystems [11,70].

### 1.2. Exposure Pathways and Uptake Mechanisms in Aquatic Organisms

#### 1.2.1. External Exposure Routes

Aquatic organisms are externally exposed to both primary and secondary MPs, as well as NPs, through multiple routes. A comprehensive review by Li et al. [71] reports the widespread occurrence of plastics across major aquatic taxa, including both invertebrates and vertebrates (fish being the most extensively studied aquatic organisms), resulting from the uptake of plastic particles spanning a wide size range through filter feeding, direct ingestion, gill capture, and trophic transfer. Plastics have been detected in fish prey items and benthic soils, thereby impacting benthic organisms and associated food webs [72]. One major concern is that aquatic organisms may misidentify MP particles as prey, leading to blockages in the digestive tract and reduced nutritional intake as well as the uptake, transfer, and bioaccumulation of toxic chemicals [73,74,75]. MP particles in wild-caught fish ranging from 11 to 4011 μm (mean ± SD: 205 ± 326 μm) have been detected accumulating in fish, accounting for 3.4–90.2% of the total MP particles counted [76,77,78,79]. Aquatic invertebrates can also ingest MPs and NPs through filter feeding, by mistaking them for natural prey items and via uptake across the gills [80,81,82]. These fish and aquatic invertebrates (e.g., mussels and clams) can accumulate plastics that can then be transferred to humans during consumption [83,84,85,86]. Generally, individuals with higher activity levels or larger body sizes, due to increased feeding rates, ingest and accumulate more MPs [71]. Plastics readily sorb a wide range of environmental contaminants from water and sediment, including polychlorinated biphenyls (PCBs), polycyclic aromatic hydrocarbons (PAHs), persistent organic pollutants (POPs), metals (e.g., arsenic [As], cadmium [Cd], copper [Cu], lead [Pb], zinc [Zn]), and dioxins [87,88,89,90]. Over 50% of plastic types have been shown to consist of hazardous monomers, additives, and byproducts, which can leach out [55,91]. Consequently, both vertebrates and invertebrates can uptake plastics and their sorbed contaminants from aquatic environments; once internalized, these contaminants may desorb from the plastic particles accumulated in the body, leading to additive toxic effects [88,92].

#### 1.2.2. Internal Translocation and Tissue Accumulation

MPs and NPs can be absorbed across gill and digestive membranes, be transported through the circulatory system of aquatic organisms, traverse epithelial barriers, and translocate and accumulate into various tissues and cells [93]. In addition, these plastic particles can act as vectors for previously sorbed contaminants, thereby enhancing their potential toxicity [15]. Once internalized, MPs and NPs accumulate in multiple organ systems, including the gut, liver, gills, brain, and reproductive tissues [24]. The liver often acts as a major deposition site due to its central role in detoxification and circulation, while persistence in the gut can lead to localized inflammation and barrier dysfunction [94,95]. Translocation of MP and NP particles occurs when MPs are <20 µm, as those ≥20 µm have been shown to collect on gill and intestinal tissue surfaces [96]. MPs and NPs may cross the intestinal epithelium via paracellular transport, endocytosis, or M cell–mediated uptake, subsequently entering the circulatory or lymphatic systems [97,98,99]. Similarly, branchial uptake through gill epithelia allows particles to bypass digestive processing and directly access systemic circulation [100]. Particle size, surface charge, and functionalization strongly influence translocation efficiency, with NPs exhibiting higher bioavailability and tissue penetration than larger MPs [101,102,103]. Once in the blood, MPs and NPs are small enough to cross the blood–brain barrier, as particles were found in brain tissue after laboratory exposure [23,104,105]. NP particles can be internalized by neurons and glial cells, and exposure to MPs and NPs induced OS, inhibited acetylcholinesterase (AChE) activity, altered neurotransmitter levels, and decreased mitochondrial function [23,29,105,106]. These neural alterations may translate into behavioral changes, as multiple behavioral effects have been reported following MP exposure [23,80,105,107]. Additionally, the transfer of plastics and contaminants can result in several types of adverse effects, including pathological damage, lysosomal membrane destabilization, DNA damage, apoptosis, and inflammatory response [108,109,110,111]. Additionally, translocation to reproductive organs has been documented, suggesting potential impacts on gametogenesis and transgenerational effects [112]. Together, these findings highlight the capacity of MPs and NPs for internal transport and tissue-specific accumulation, thereby driving neurobehavioral dysfunction and long-term physiological consequences.

## 2. Methods

### Literature Search and Inclusion Criteria

To review the neurotoxic potential of MP and NP particles, a literature search was conducted to cover articles in the database PubMed between 1 December 2019 and 30 December 2025, though some 2026 preprints were also included. This date range was used as a literature review by Prϋst et al. [23], who thoroughly covered the neurotoxic effects of MPs and NPs before 1 December 2019. We refer you to that review paper for a comprehensive look at plastics-induced neurotoxicity before 1 December 2019. This review reflects on those findings and summarizes findings since then. To examine recent findings, the following combinations of search words were used in PubMed: Neurotox* AND Microplastic* AND Aquatic* (130 research papers were retrieved, including 34 review articles, 1 modeling study, 2 retracted papers, 15 studies involving terrestrial animals, 5 studies without neurotoxic endpoints, and 1 non-English article that was excluded). From this search, 72 original research articles met the inclusion criteria; Neurotox* AND Microplastic* AND Aquatic* AND Behavior* (no new papers were retrieved); Neurotox* AND Nanoplastic*AND Aquatic* (5 new research papers were identified; of which 1 was a review and excluded, resulting in 4 additional studies); Neurotox* AND plastic particles AND Aquatic* identified 5 new research papers; however, 1 was excluded due to the use of terrestrial animals, so a total of 4 additional studies were included in this review. This review excluded studies that did not evaluate neurotoxic endpoints or involve plastic exposure in laboratory or field experiments.

In total, 80 articles met the inclusion criteria. Among these, 57 investigated physiological endpoints related to OS and inflammation. Thirty-three studies examined cellular and molecular markers of neurotoxicity, 60 evaluated neurochemical alterations, 31 reported genetic changes, and 31 assessed behavioral outcomes. Several studies addressed multiple endpoint categories; therefore, these totals are not mutually exclusive. This review synthesizes the findings reported in the results sections of the studies included herein. The review is organized to systematically evaluate plastics-induced effects across four principal domains: physiological alterations, neurotoxic outcomes, genetic modifications, and behavioral endpoints.

The majority of studies concentrated on fish, primarily zebrafish (*Danio rerio*) embryos and larvae, and mollusks, with additional representation from other aquatic invertebrate and vertebrate taxa. The plastics most commonly examined included PS, PE, PET, polyethylene vinyl acetate (PEVA), HDPE, LDPE, and PVC. Several studies also exposed organisms to bio-based MPs, including polylactic acid (PLA), while others employed plastics of unknown polymer composition. Several studies (43) investigated the (neuro)toxicity of MP or NP co-exposed with other substances: Copper (Cu; 5 studies) and other metals (Cadmium (Cd), Arsenic (As), Lead (Pb), Silver (AgNPs), Zinc (Zn), Methylmercury (MeHg)), bisphenol A (BPA; 3 studies), the antimicrobial/antifungal agent Triclosan (TCS; 2 studies), antibiotics (Ciprofloxacin (CIP; 2 studies), Sulfamethoxazole (SMX), Oxytetracycline (OTC), Levofloxcin, and Enrofloxacin (ENR)), insecticides (Chlorpyrifos, Clothianidin, Thiamethoxam (TMX), and Abamectin), flame retardant chemicals Tris(1-chloro-2-propyl) phosphate (TCPP) and (2,2′,4,4′-tetrabromodiphenyl ether (BDE-47)), Perfluorooctanoic Acid (PFOA; 2 studies), the sunscreen component Avobenzone, the β-blocker propranolol (PRP), PAHs, polychlorinated biphenyls (PCBs), the antidepressants Fluoxetine, the fungicide thifluzamide (TF), estrogen receptor antagonist ICI 182,720 (ICI), the herbicide Metolachlor (MET), the industrial chemicals Pyrogallol (PG) and Acrylamide, the imaging dye/rare earth element Gadolinium and the plasticizer metabolite mono-(2-ethylhexyl) phthalate (MEHP). Although co-exposure studies represent over half of the results reviewed herein, the diversity of co-exposed substances and their multilayered effects preclude strong conclusions about synergistic effects of microplastic co-exposure in our review. Additionally, papers examining changes in plastics charge, varying salinity, MP/NP aging/ultraviolet exposure, turbidity, and the presence of Biochar with NPs were also examined. The specific results of these studies are summarized in Tables S1–S5 and synthesized in the text. Specifically, Table S1 presents findings on OS and inflammation, Table S2 on cellular and molecular neurotoxicity, Table S3 on neurochemical disruptions, Table S4 on genetic changes, and Table S5 on behavioral modifications. A summary of these findings is presented in Table 1. Some results summaries and images found in the graphical abstract, tables, and figures were generated in part using Chat GPT 5.2 [113].

## 3. Physiological Impacts of MPs and NPs

**Figure 1:**
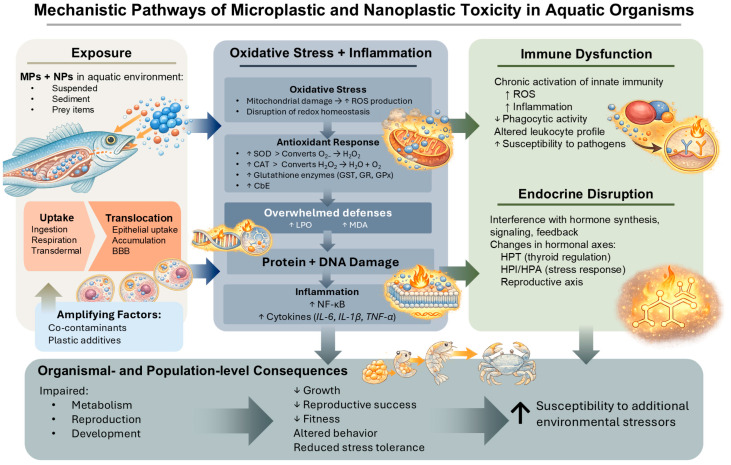
Summary of the mechanistic pathways by which MPs and NPs induce toxicity in aquatic organisms (↑ indicates an increase; ↓ indicates a decrease). Following environmental exposure, MPs and NPs are taken up through ingestion and epithelial absorption (gills and gut) and distributed to internal tissues, including the liver, brain, and gonads. Cellular internalization disrupts mitochondrial function, leading to increased ROS production and OS, which activate antioxidant defenses (e.g., SOD, CAT, and glutathione-related enzymes). Prolonged or high-dose exposure overwhelms these protective systems, resulting in LPO (elevated MDA), protein and DNA damage, and activation of inflammatory signaling pathways such as NF-κB, along with altered CYP450–mediated detoxification. These processes contribute to metabolic dysregulation, impaired growth and reproduction, immune dysfunction, and endocrine disruption across multiple hormonal axes. Collectively, these mechanistic effects scale to population-level consequences, including reduced fitness, reproductive failure, altered stress tolerance, and increased susceptibility to additional environmental stressors.

Growing evidence indicates that MPs and NPs exert multifaceted physiological toxicity in aquatic organisms through OS and inflammation, metabolic and reproductive dysregulation, and immune and endocrine disruption (Figure 1).

### 3.1. Oxidative Stress and Inflammatory Responses

Exposure to MPs and NPs can disrupt redox homeostasis in aquatic organisms; like other xenobiotics, these particles can stimulate biochemical pathways that enhance reactive oxygen species (ROS) production, often through mitochondrial damage in exposed cells, ultimately leading to OS [109,114,115,116,117,118]. Several studies suggest that OS may be a key mechanism underlying MP toxicity across a wide range of organisms ([115,117]; see Table S1). Laboratory and field studies show that accumulation of MPs and NPs disrupts redox homeostasis and increases the production of ROS through various intracellular and extracellular mechanisms [115,116,117,118]. Elevated ROS levels can lead to lipid peroxidation (LPO), protein oxidation, altered gene expression, and disruptions in cellular redox status, ultimately contributing to DNA damage, disease and premature aging of aquatic organisms [117,119,120]. The most important enzymes for the detoxification of ROS in all organisms are superoxide dismutase (SOD), catalase (CAT), glutathione-related enzymes (e.g., glutathione-S-transferase (GST), glutathione reductase (GR), and glutathione peroxidase (GPx)), and carboxylesterase (CbE) [121]. These defense systems are often upregulated in response to ROS; however, prolonged or high-dose exposure to xenobiotics can overwhelm these protective mechanisms and result in cellular damage [122,123]. SOD is considered a first line of defense and indicates an early cellular response to increased ROS as it converts the free radicals (SO^−^) into hydrogen peroxide (H_2_O_2_) [117,120]. Increased SOD activity indicates that contaminant exposure is increasing oxidative pressure [122]. CAT is often measured alongside SOD activity as CAT breaks down H_2_O_2_ into water and oxygen. By measuring CAT activity, the cell’s ability to break down H_2_O_2_, generated by SOD activity, can be assessed. Collectively, examining SOD and CAT activity reveals whether antioxidant defenses are functioning in balance or becoming overwhelmed [122]. NPs, due to their smaller size and higher surface reactivity, generally induce stronger oxidative responses than larger MPs, likely because of enhanced cellular uptake and subcellular localization [109,124]. Further, CbE and GST play critical roles in detoxification by participating in phase I and phase II biotransformation, respectively, thereby facilitating the conversion and removal of harmful substances from cells [121]. Malondialdehyde (MDA) is a toxic end product of LPO, a process in which ROS damage lipids in cell membranes. Because elevated MDA levels directly reflect the extent of LPO, MDA is widely used as a reliable biomarker of ROS-induced membrane damage [119]. Unlike SOD and CAT, measuring MDA allows us to determine if cellular damage has occurred post-exposure to MPs and NPs, as LPO compromises essential cellular functions. Additionally, 8-hydroxy-2′-deoxyguanosine (8-OHdG) is used as a biomarker for endogenous oxidative damage to DNA [125]. Accordingly, the amount of MDA and 8-OHdG is widely regarded as a biomarker of contaminant exposure, and exposure to MPs and NPs has caused elevations in these biomarkers ([87,109,126,127,128,129,130,131,132,133,134,135,136], see Table 1 and Table S1).

OS is closely linked to inflammation, as MP- and NP-induced ROS can activate pro-inflammatory signaling pathways such as NF-κB, which serves as a key molecular bridge between OS and inflammatory responses [134,135]. Activation of these pathways leads to increased expression of inflammatory cytokines (e.g., interleukin-6 (*il-6*), interleukin-1 beta (*il-1β*), and tumor necrosis factor-alpha (*tnf-α*)), as well as chemokines and other inflammatory mediators in brain, gill, gut, and liver tissues [136]. Two typical detoxification enzymes, 7-Ethoxyresorufin-O-deethylase (EROD) and Benzyloxy-4-trifluoromethylcoumarin-O-debenzyloxylase (BFCOD), reflect the activity of cytochrome P450 (CYP450), a crucial player in the detoxification process of external substances [137]. Acid phosphatase (ACP), alkaline phosphatase (ALK) and lysozyme (LYZ) activities are key indicators of an innate immune response, especially in aquatic animals and invertebrates, reflecting the body’s defense status against pathogens or stressors, with changes in their activity signaling immune activation or impairment [138]. Reduced detoxification efficiency hinders the removal of plastics from the body, potentially leading to heightened inflammatory responses. This plastic-induced inflammation leads to tissue damage, immune system infiltration, autophagy, and lysosomal membrane destabilization [139]. Collectively, OS and inflammation are recognized as key mechanisms underlying MP- and NP-induced toxicity in aquatic organisms. These processes may contribute to impaired physiological function, reduced growth and fitness, endocrine disruption, induction of apoptosis, altered immune responses, reproductive disturbances, and increased susceptibility to additional environmental stressors [135,140,141,142,143].

### 3.2. Effects on Metabolism, Growth, and Reproduction

Xenobiotic contaminants in aquatic environments are increasingly recognized for their ability to disrupt metabolism, growth, and reproductive function in aquatic organisms [21,144,145]. Upon ingestion or internalization, MPs and NPs can interfere with energy metabolism by altering lipid, carbohydrate, and protein pathways, often through OS–mediated mitochondrial dysfunction and altered enzyme activity, leading to increased energetic costs for maintenance and detoxification at the expense of growth [146,147]. Chronic exposure has been associated with reduced somatic growth, delayed development, and impaired condition in fish and invertebrates, particularly during early life stages when metabolic demands are high [148,149,150,151,152]. Reproductive endpoints are also adversely affected, with MPs and NPs shown to disrupt gametogenesis, reduce fecundity and egg quality, alter sex hormone levels, and impair spawning success in multiple taxa, including fish, mollusks, and crustaceans [142,153,154,155]. These effects are further exacerbated by the ability of MPs and NPs to act as vectors for co-contaminants and additives with endocrine-disrupting properties, amplifying metabolic and reproductive toxicity [156,157]. Choi et al. [158] found that PET microfibers (MF) negatively affected estradiol and testosterone and the gonadal index of mussels (*Mytilus galloprovincialis*), suggesting that long-term exposure can lead to reproductive failure. Collectively, evidence suggests that MP- and NP-induced metabolic dysregulation and reproductive impairment may have population-level consequences and pose significant risks to aquatic ecosystem stability.

### 3.3. Immune Disruption and Endocrine Effects

The widespread presence of MPs and NPs in aquatic environments has been accompanied by increasing evidence of their adverse effects on immune function and endocrine regulation in aquatic organisms [145,159,160]. Following tissue uptake, plastic particles can activate innate immune responses, characterized by elevated production of ROS, inflammatory cytokines, and altered expression of immune system-related genes, resulting in inflammation and immunotoxicity in fish and aquatic invertebrates [161,162]. Chronic exposure suppresses immune competence through impaired phagocytic activity, dysregulated leukocyte populations, and heightened susceptibility to pathogens. Moreover, MPs and NPs act as endocrine-disrupting agents by interfering with hormone synthesis, receptor signaling, and feedback regulation within the hypothalamic–pituitary–thyroid (HPT), hypothalamic–pituitary–interrenal (HPI/HPA), and reproductive axes, thereby altering thyroid, stress, and sex steroid hormone levels [160,163,164,165]. These endocrine effects are frequently linked to OS and inflammatory signaling pathways, such as NF-κB, which serve as mechanistic bridges between immune activation and hormonal dysregulation ([124,125]; see Table 1 and Table S1, and Figure 1). Additionally, the ability of MPs and NPs to adsorb and transport endocrine-disrupting chemicals and plastic additives may further exacerbate immune and endocrine perturbations, amplifying their toxicological impacts at individual and population levels [144,156,157,163].

## 4. Neurotoxic Effects of MPS and NPS

**Figure 2:**
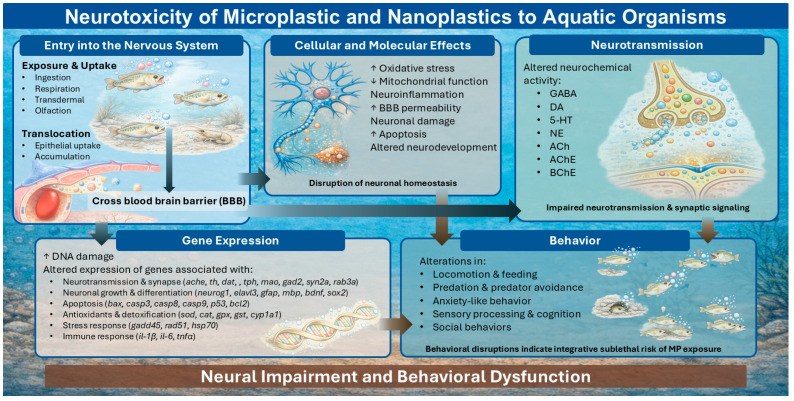
Neurotoxic effects of MPs and NPs in aquatic organisms. Schematic overview illustrating major entry routes of MPs and NPs into aquatic organisms, including ingestion, gill uptake, olfactory exposure, and transdermal absorption, followed by systemic distribution and translocation to neural tissues; cellular and molecular mechanisms of neurotoxicity, including OS, mitochondrial dysfunction, neuroinflammation, and neuronal damage; alterations in neurotransmission, and downstream genetic and behavioral consequences, including DNA damage, altered gene expression, and behavioral impairments in fish. Collectively, the figure highlights multilevel mechanisms linking MP/NP exposure to neurobehavioral dysfunction in aquatic organisms (↑ indicates an increase; ↓ indicates a decrease).

MPs and NPs have emerged as pervasive neurotoxic contaminants, capable of entering the nervous system and disrupting neural homeostasis across multiple levels of biological organization. This section synthesizes current evidence on the pathways of MP/NP entry into neural tissues, the ensuing cellular and molecular mechanisms of neurotoxicity, and resulting behavioral impairments observed in exposed aquatic organisms (Figure 2).

### 4.1. Entry of MPs/NPs into the Nervous System

In aquatic environments, plastic particles vary widely in polymer type, chemical composition, size, and shape; all factors that critically influence their capacity to cross biological membranes. These particles enter aquatic organisms via direct and indirect ingestion, gill uptake, olfactory exposure, and transdermal exposure, and can subsequently translocate across biological barriers (via endocytosis, phagocytosis, or micropinocytosis) into the nervous system, where they induce OS and inflammatory responses [23,24,25,26,27,28]. The toxicity of plastic particles is influenced by weathering and aging processes, in addition to environmental conditions such as temperature, salinity, and water velocity in aquatic systems [92,166,167,168,169,170]. Moreover, their toxicity can be modulated by surface charge (e.g., anionic carboxyl [–COOH] or cationic amino [–NH_2_] groups), functional groups, and by the sorption of co-occurring contaminants or pharmaceuticals, which may either exacerbate or mitigate toxic effects [169,171,172,173]. Additionally, size of plastic particles impacts whether they translocate across biological membranes, with larger particles (MPs > 20 µm) collecting on intestinal membranes [96]. MPs (<20 μm) and NP particles have an increased ability to cross into the plasma membrane, enter the bloodstream and cross the blood brain barrier (BBB) and translocate into the brain, nervous system and other essential organs [174,175]. Lin et al. [176] demonstrated in a video that 20 nm PS-NPs were able to cross into the bloodstream of zebrafish embryos, subsequently accumulating in neural tissues, resulting in cellular damage and an increased vulnerability to develop neuronal disorders and alter behavior. These internalized plastics can damage the BBB, which can be influenced by the charge of the plastic and sex of the animal. Teng et al. [171] demonstrated that exposure to PS-NPs resulted in BBB disruption across all PS-exposed groups, characterized by irregular morphology and rupture of the double-membrane structure. This compromised barrier integrity was associated with increased accumulation of PS-NPs in the reticular formation (RF) and ventral hypothalamus of zebrafish. Accumulation of PS-NPs also led to damage in several brain regions of zebrafish embryos [177]. In invertebrates, following uptake, MPs and NPs can migrate along hemolymph pathways and accumulate directly into organs and tissues, causing dysfunction [115,168,178,179,180]. Collectively, Table S2 illustrates that MPs and NPs can penetrate nervous tissues in both vertebrate and invertebrate aquatic organisms, providing a mechanistic foundation for observed neurobehavioral and neurophysiological effects reported across diverse species.

### 4.2. Cellular and Molecular Neurotoxicity

A wide range of neurotoxic effects occur in aquatic invertebrates and vertebrates following exposures to MPs and NPs through interconnected cellular, molecular, and neurochemical mechanisms (see Table 1, Tables S2 and S3). Collectively these studies demonstrate that MPs and NPs accumulate systemically, preferentially in the digestive tract but with secondary accumulation in neural tissues, particularly under prolonged exposure or co-exposure scenarios. This accumulation is closely associated with structural damage to brain and sensory tissues, BBB disruption, altered neurodevelopment, endocytosis, and genotoxic effects, including oxidative DNA damage and apoptosis [128,157,161,171,177,181,182]. Tissue accumulation increased when animals were exposed to aged plastics and alterations in brain and nervous system histopathology including edema and cell damage occurred post-exposure [92,157,173,176,177]. Early life stages appear particularly vulnerable, with embryonic and larval exposures leading to reduced brain size, disrupted motor neuron development, sensory organ damage, and altered neural differentiation [157,165,183]. These alterations are exacerbated under chronic conditions or in the presence of other contaminants [75,87,129,130,177,184,185]. Several studies demonstrate that MP and NP exposure can also enhance the bioaccumulation and toxicity of metals, pesticides, and pharmaceuticals, resulting in molecular stress responses and genomic changes [92,127,128,133,161,181,183,186]. The plastic-induced cellular changes reported in Table S2 can be a result of damage associated with OS and may then lead to changes in neurotransmitter release and modulation [128,133,165,168,185,187,188].

Neurochemical disruption represents a central mechanistic pathway linking these cellular effects to functional impairment (Table S3). In aquatic invertebrates, MPs and NPs frequently alter cholinergic signaling, most commonly through changes in AChE and butirylcholinesterase (BChE) activity [129,170,184,189,190]. Both inhibition and induction of AChE have been reported, reflecting species-specific sensitivity, exposure duration, particle properties, and the influence of co-contaminants [92,173,191]. Inhibition of AChE, particularly under co-exposure conditions, suggests impaired neurotransmission and potential overstimulation of cholinergic pathways, while elevated enzyme activity may represent compensatory responses to oxidative or inflammatory stress [128,157,161,173]. Alterations in nitric oxide (NO) signaling and neurotransmitter precursors such as BChE further indicate broad interference with neural communication [129,180]. In vertebrates, MPs and NPs consistently disrupt neurotransmitter homeostasis and associated gene expression, with reported alterations in acetylcholine (ACh), dopamine (DA), serotonin (5-HT), γ-aminobutyric acid (GABA), norepinephrine (NE), and melatonin pathways [128,142,163,165,166,192,193,194,195]. NPs generally exert stronger neurochemical effects than MPs, with smaller and positively charged particles producing greater enzyme inhibition, neurotransmitter imbalance, and transcriptional dysregulation [196]. Multiple studies report concurrent suppression of AChE activity and altered expression of genes involved in dopaminergic, serotonergic, and GABAergic signaling, indicating impaired synaptic function and neural development [127,142,195]. Co-exposure with metals, pesticides, and pharmaceuticals frequently intensifies these effects by increasing contaminant bioavailability and amplifying neurochemical and molecular stress responses ([86,178,183,185]; Table S3). Collectively, the evidence summarized in Table 1, Tables S2 and S3 demonstrates that MPs and NPs disrupt neural systems across aquatic taxa through overlapping cellular, molecular, and neurochemical mechanisms. These perturbations provide a mechanistic foundation for the behavioral impairments consistently reported following plastic exposure and underscore the importance of integrating molecular and neurochemical endpoints when assessing the neurotoxic and ecological risks of plastic pollution.

### 4.3. Genetic Alterations

Collectively, genetic evidence across aquatic invertebrates and vertebrates indicates that MPs and NPs act as broad-spectrum molecular stressors, increasingly linking transcriptomic and epigenetic alterations in the nervous system to early molecular events underlying neurotoxicity. Moreover, co-exposure to environmental contaminants like metals, pesticides, pharmaceuticals, or other environmental stressors consistently amplifies gene dysregulation. NPs, aged plastics, and chemically functionalized particles produce stronger and more persistent transcriptional disruptions, highlighting their heightened ecological and neurotoxic risk. The most conserved and sensitive genetic targets include OS defenses, apoptotic pathways, neurodevelopmental regulators, and neurotransmitter systems (Table 1 and Table S4). In both aquatic invertebrates and vertebrates, MPs and NPs modulated antioxidant and detoxification genes (e.g., *sod*, *cat*, *gpx*, *gst*, *cyp1a1*), with early adaptive upregulation often giving way to suppression under higher doses, chronic exposure, or combined stress conditions, indicating overwhelmed cellular defenses [92,110,133,167,170,177,192,197,198]. Apoptosis-related genes (*bax*, *casp3*, *casp8*, *casp9*, *p53*, *bcl2*) were broadly dysregulated, with co-exposures and NPs, especially aged or surface-modified particles, producing the strongest pro-apoptotic signatures in both invertebrates and vertebrates [128,157,170,196,197,198,199]. Suppression of DNA repair and stress response genes (*gadd45*, *rad51*, *hsp70*) further suggests compromised genomic stability under severe or combined exposures [128,161,170,197,198,200,201].

Neurodevelopmental and neurotransmission-related gene regulation is also a sensitive target of MP and NP toxicity in vertebrates. Disruptions are consistently observed in cholinergic (*ache*) [167,176,192,197,198], dopaminergic (*th*, *dat*) [157,195], serotonergic (*tph*, *mao*) [193,202], GABAergic (*gad2*) [195], and synaptic genes (*syn2a*, *rab3a*) [176,191,203], often accompanied by altered expression of neuronal growth and differentiation markers (*neurog1*, *elavl3*, *gfap*, *mbp*, *bdnf*, *sox2*) [128,161,171,172,173,181,191,192]. Co-exposure scenarios frequently intensified these transcriptional disturbances, revealing synergistic or additive neurotoxic effects that extended to endocrine and immune signaling pathways, including HPT/HPA axis genes and inflammatory cytokines (*il-1β*, *il-6*, *tnfα*) [92,142,171,204]. Collectively, these findings indicate that MPs and NPs function as pervasive molecular stressors across aquatic taxa, with NPs, aged plastics, and chemical co-contaminants posing heightened risks for long-term neurotoxicity, impaired development, behavior, and reduced organismal resilience.

### 4.4. Behavioral Consequences

Across aquatic invertebrates and vertebrates, MPs and especially NPs consistently cause alterations to locomotor activity [142,205,206], feeding behavior [106,207], anxiety-like behavior, sensory processing [127,142,161,163,181,192,195,204,208], cognition [183], aggression [142,209], and social behaviors [142,209] (Additional examples are summarized in Table S5). Such changes in behavior are likely related to neurotoxic effects of MPs and NPs on underlying neural mechanisms, as particle size, surface chemistry, aging, and polymer type strongly modulate behavioral outcomes [23,105]. Behavioral impairments were negatively correlated with neurotransmitter levels, linking altered gene expression, neurotransmitter imbalance, and neurobehavioral toxicity [157,161,163,166,176,191,192,194,195,196,203,204]. Co-exposure with environmental contaminants often exacerbates neurobehavioral toxicity, revealing MPs/NPs as both stressors and vectors [92,133]. Behavioral endpoints emerge as early, integrative indicators of ecological risk, highlighting the importance of incorporating behavior into environmental plastic risk assessments. In invertebrates, MP and NP exposure alters key functional behaviors such as burrowing, filtration, and feeding, often without affecting overall survival or gross mobility [131,197,210]. These effects are frequently linked to neurophysiological disruption, including reduced AChE activity, altered neurotransmitter levels, impaired neuromuscular coordination, and OS, particularly in polychaetes and bivalves [23,130,210]. Environmentally derived, aged, or smaller-sized particles generally induce stronger behavioral alterations than pristine (or virgin) MPs, indicating that particle characteristics strongly influence biological outcomes [131,197]. Collectively, these findings establish a mechanistic and ecological foundation for examining how MP and NP exposure disrupts neurobehavioral processes. These effects on complex behaviors may have direct implications for fitness, population dynamics, and ecosystem function.

In aquatic vertebrates, particularly amphibians and fish, MPs and NPs induce pronounced alterations in locomotion, anxiety-like behavior, sensory responsiveness, learning, memory, and social interactions, with NPs typically exerting greater neurobehavioral toxicity than larger MPs [171,187,194,196,211]. Behavioral responses include hypoactivity or hyperactivity, disrupted circadian locomotor rhythms, impaired escape and startle responses, increased thigmotaxis, and altered predator avoidance [142,157,163,212,213]. Changes in shoaling behavior and cohesion suggest that plastic exposure alters cognition [171,183]. Co-exposure with co-occurring environmental contaminants, such as antibiotics, pesticides, metals, endocrine disruptors, and persistent organic pollutants, frequently results in additive or synergistic behavioral impairment, often when single-compound exposures produce minimal effects [127,161,165,166,191]. Further, ER receptor antagonism can reverse NP-induced behavioral deficits, suggesting endocrine-neural crosstalk [157]. Notably, some behavioral disturbances persist after depuration and can extend to subsequent generations following chronic exposure, underscoring the potential for long-term and transgenerational neurobehavioral consequences of plastic pollution, where offspring of polystyrene anionic carboxyl (PS-COOH)-exposed parents exhibited increased activity and swimming velocity. Because exposure to MPs and NPs consistently disrupts behavior in both aquatic invertebrates and vertebrates (Table 1 and Table S5), behavioral endpoints represent a sensitive and ecologically relevant measure of plastic toxicity, as such alterations can impair environmental fitness and adversely affect feeding, reproduction, and survival [214,215,216].

**Table 1:** Mechanistic synthesis of physiological, neurotoxic, and behavioral alterations induced by MP/NP exposure. Reported effects are organized into standardized mechanistic domains spanning neurotransmission, oxidative metabolism, neurotoxicity, neurovascular integrity, neuroendocrine signaling, and behavior. Direction denotes qualitative consensus across studies (↑ indicates an increase; ↓ indicates a decrease). Reference lists are exhaustive for each endpoint. For further details, please refer to Tables S1–S5.

Domain	Effect	Endpoints	Direction	References
Neurotransmission	Cholinergic signaling	AChE/BChE/ChE activity, gene expression altered	Mixed	[75,87,92,100,123,126,128,129,130,132,142,157,158,161,163,165,166,167,168,169,170,176,179,183,184,185,186,188,189,190,191,192,194,195,197,198,203,204,208,213,217,218,219,220,221,222,223,224,225,226,227,228,229,230,231,232,233]
	Monoaminergic signaling	DA and/or 5-HT activity, gene expression altered	Mixed	[128,142,157,163,165,166,177,179,192,193,194,195,196,202,204,222]
	Excitatory/Inhibitory balance	Glutamate and/or GABA signaling, gene expression altered	Mixed	[142,165,166,187,192,194,195,204,222]
Oxidative metabolism	Oxidative stress	ROS and OS markers (e.g., LPO, MDA)	Predominantly ↑	[87,92,123,126,127,128,129,130,131,132,133,142,158,161,166,168,177,181,185,186,187,188,190,191,192,193,197,199,200,201,202,203,208,212,218,226,228,231,232,233,234,235,236]
	Antioxidant defense	Antioxidant enzyme activity (SOD, CAT, GPx, GST, GSH), TAC altered	Mixed	[75,87,92,100,123,126,127,129,130,131,132,133,158,161,166,167,168,170,177,179,180,184,185,186,187,188,189,190,191,192,193,197,198,199,200,201,202,203,210,217,218,219,221,222,225,226,228,231,232,233,234,236]
Neurotoxicity	Apoptotic signaling	Apoptosis-related markers (e.g., caspase) increased	Predominantly ↑	[128,157,161,170,177,181,196,197,198,199,202,235]
	Neuronal structure and development	Histopathological brain alterations (e.g., degeneration, edema, lesions), developmental gene expression	Direction not applicable	[126,127,128,157,161,163,168,171,172,173,176,177,181,191,195,203,204,212,222]
	Neuroinflammation	Pro-inflammatory signaling (e.g., cytokines, EROD, CYP450) altered	Predominantly ↑	[92,100,126,132,142,161,169,171,173,180,181,195,198,201,204,208,222,231]
	Genotoxic stress	DNA damage increased	Predominantly ↑	[127,170,173,177,185,198]
	Neurovascular integrity	BBB permeability altered, translocation to CNS	Permeability ↑	[171,176,196]
	Neuroendocrine signaling	Stress-axis/hormonal signaling (e.g., cortisol), gene expression altered	Mixed	[163,170,196,198,200,201,203]
	Neural accumulation	MP particles translocate to and accumulate in brain/CNS	Predominantly present following exposure	[75,100,127,163,168,169,171,173,176,194,196,204,224,231,237]
Behavior	Motor behavior	Locomotor activity altered (swimming/movement levels)	Mixed	[127,131,142,157,161,163,165,166,171,172,176,181,187,191,192,193,194,195,196,204,208,211,212,213,236,237]
	Feeding behavior	Feeding or ingestion behavior altered	Mixed	[130,131,187]
	Anxiety-like behavior	Anxiety-like or stress-related behavior altered	Mixed	[142,157,161,163,165,171,181,183,187,192,193,204,208,211,212,213]
	Cognitive function	Learning and memory impaired	Performance ↓	[171,183,193,195]
	Social behavior	Social interaction or aggression altered	Mixed	[142,171,183,224]
	Predator avoidance	Antipredator responses impaired	Mixed	[176,224]
	Burrowing/burying behavior	Substrate interaction behavior (e.g., burrow latency) altered	Mixed	[197,210]

## 5. Ecological and Evolutionary Implications

### 5.1. Impacts on Population Dynamics and Community Structure

Plastics-induced nervous system alterations, including impaired neurodevelopment, disrupted neurotransmission, and sensory and motor dysfunction, can compromise feeding efficiency, predator avoidance, habitat use, and mating behaviors, thereby reducing individual fitness and reproductive success [3,10,51,238]. These effects can manifest as transgenerational impacts in the offspring of exposed aquatic organisms, potentially impairing the fitness of affected populations [171,218,239]. For example, larval zebrafish derived from exposed parents exhibited altered hatching rates, OS markers, neurodevelopmental, inflammation, apoptosis, and changes in behavior, including increased swimming activity and speed [171,239]. Thus, the consequences of contamination can have far-reaching ecological and evolutionary implications for aquatic invertebrates and vertebrates by affecting survival, fitness, reproduction, and population dynamics. These neurobehavioral impairments occur alongside bioaccumulation of MPs/NPs and associated contaminants, which can be transferred across trophic levels through predator–prey interactions, increasing exposure in higher-order consumers and magnifying toxic effects within food webs [23]. Chronic exposure has been linked to reduced growth, fecundity, and offspring viability, with consequences that may persist across life stages and generations [240]. At the population level, the combined impacts of neurotoxicity, bioaccumulation, and altered trophic interactions can reduce recruitment, shift population structure, and impose selective pressures that may drive evolutionary responses, ultimately destabilizing aquatic communities and ecosystem function [21,241].

### 5.2. Potential for Evolutionary Responses or Adaptation

Chronic exposure to MPs and NPs has the potential to impose multiple selection pressures on aquatic invertebrates. In aquatic invertebrates such as crustaceans, mollusks, and zooplankton, plastic exposure can reduce feeding efficiency due to accumulation in the digestive tract, slow growth, impair reproduction, and cause morphological deformities and developmental delays [80,112,152,218]. These effects can select for individuals that discriminate or avoid MPs, maintain energy balance, or reproduce successfully despite exposure [242]. As chronic exposure to MPs and NPs induces OS, including ROS production, LPO, and induction of antioxidant defenses (Table S1), increased plastics pollution may favor species that have more robust detoxification pathways. Further, continued exposure to MPs in highly polluted areas could lead to future tolerance in some aquatic species as seen with exposure to pesticides [243]. Behavioral changes, such as altered swimming, burrowing, or predator avoidance, further contribute to selective pressures, with organisms able to maintain normal behaviors likely having higher survival and fitness. Collectively, these physiological, behavioral, and reproductive pressures may drive population-level shifts, potentially favoring organisms that tolerate MP/NP exposure and altering interactions within aquatic ecosystems.

## 6. Future Directions and Recommendations

### 6.1. Challenges and Limits in Studying MP/NP Neurotoxicity

Despite the growing body of literature on MP and NP neurotoxicity in aquatic invertebrates and vertebrates, future research priorities should emphasize the standardization of experimental methodologies, particularly with respect to particle characterization (e.g., size, shape, polymer type, surface chemistry, and aging state) and the use of environmentally relevant exposure concentrations, to improve reproducibility and facilitate meaningful cross-study comparisons [244]. Greater integration of neurobiology, ecotoxicology, and environmental chemistry is needed to mechanistically link particle fate and transport with uptake, neurotoxicity, and ecological impacts across trophic levels. The adoption of integrative tools, including multi-omics approaches, advanced imaging techniques, and machine learning-based data integration, will enhance the ability to identify molecular pathways, spatial accumulation patterns, and predictive toxicity signatures associated with MP and NP exposure. Despite the important progress in analysis of the toxicity of MPs, detection technologies for identifying nano-sized plastic particles are still lacking, and therefore should be developed swiftly as NPs have been identified as highly neurotoxic [23,245,246].

### 6.2. Major Knowledge Gaps

In aquatic environments, MPs are highly heterogeneous, encompassing diverse polymer types, shapes, and sizes, including weathered and charged forms, and often co-occur with other contaminants capable of adsorbing to or being absorbed by these particles. Currently, systematic comparisons of the neurotoxic effects of MPs and NPs across varying polymer types, exposure concentrations, and durations are lacking, yet are urgently needed to better elucidate their neurotoxic hazards and associated exposure risks [23]. Moreover, aquatic organisms collected from the wild frequently contain mixtures of plastic types [217,232,233], indicating that neurotoxic effects may arise from synergistic interactions among plastic polymer types, sizes, and shapes, an area that remains largely unexplored. Several studies have also shown that aged MPs and NPs, reflecting more environmentally realistic conditions, exhibit greater toxicity than virgin plastics. Yet, only 8% of the studies examined actually used aged plastics [92,165,166,169,194,219]. Additionally, other industrial or commercial chemicals, metals, pharmaceuticals, and pesticides are released into aquatic environments and can co-occur with MPs and NPs [87,88,89,90]. Variations in salinity, which are expected to shift with climate change, can enhance the dispersion of MPs in coastal ecosystems, modifying exposure patterns and potentially amplifying neurotoxic effects, yet this remains an important gap for future research [170,247]. The majority of studies summarized in Tables S1–S5 were conducted under controlled laboratory conditions, often involving exposure to only one or two additional putative contaminants. In contrast, only three studies, representing approximately 4% of those examined, have evaluated plastic-associated toxicity in wild-caught fish [217,232,233]. Lastly, toxicity is often evaluated at a single life stage, despite evidence that vulnerability to MPs and NPs varies across developmental stages [248,249,250]. Consequently, much of the existing literature may have limited environmental relevance, highlighting a critical gap between laboratory findings and real-world exposure scenarios, as more field studies are needed to validate laboratory findings.

Nearly half of the studies reviewed here include experimental treatments in which organisms are co-exposed to additional environmental pollutants alongside MPs and NPs. The chemical composition and mechanisms of action of these co-occurring pollutants are highly diverse, complicating efforts to synthesize the available evidence. Reported outcomes of co-exposure are similarly heterogeneous, with plastics exposure exacerbating toxic effects in some contexts while attenuating them in others. Nevertheless, the occurrence of synergistic responses under certain co-exposure scenarios underscores the potential for complex toxicant–particle interactions that may amplify adverse outcomes in aquatic organisms. Future work should prioritize systematic synthesis and mechanistic evaluation of co-exposure effects to better resolve how MPs interact with other environmental contaminants.

Biodegradable plastics, or bioplastics, have been proposed as an alternative to plastics as they can be broken down by microorganisms or naturally broken down by enzymes. However, bioplastics can still fragment into MPs, often referred to as biomicroplastics (BioMPs) [3,251,252]. PLA has emerged as a widely used bio-based polymer and a potential alternative to conventional plastics, including PS, owing to its mechanical performance and biodegradability, biocompatibility, and compostability [253,254]. PLA is widely used in the medical field and as packaging for food items [254,255]. Although bioplastics such as PLA are promoted as sustainable alternatives to conventional plastic, the toxicological effects and environmental safety of PLA BioMPs remain poorly understood.

Of the 80 studies included in this review, only five (6%) investigated the effects of PLA MPs (BioMPs) on aquatic organisms, underscoring the limited research in this area. Notably, all five studies reported concerning biological effects following exposure (Tables S1–S5 [75,132,189,213,224]). Both aquatic invertebrates marine rotifers (*Brachionus plicatilis*), brine shrimps (*Artemia salina*) and vertebrates (zebrafish) have been shown to accumulate PLA MPs and NPs in the brain, digestive tract, and ocular tissues, with accumulation increasing in a concentration-dependent manner [75,224]. Furthermore, exposure to these PLA BioMPs also induced OS, including SOD, CAT, alanine aminotransferase (ALT), GST activation and LPO, disrupted AChE activity, and caused changes in locomotion, social behavior, thigmotaxis, and predator avoidance in zebrafish embryos and larvae [132,189,213,224]. Hence, the neurological and behavioral changes documented in aquatic invertebrates and vertebrates warrant serious concern and highlight the need for further assessment of the short- and long-term ecological effects of PLA MPs and NPs.

Although PLA is marketed as biodegradable and environmentally friendly, biodegradation under industrial composting conditions does not necessarily translate to rapid or benign degradation in aquatic ecosystems. Fragmentation into micro- and nanoscale particles may still result in bioavailability, tissue accumulation, OS, neurotoxicity, and behavioral disruption, as observed in the limited studies available. Given that all available studies report adverse biological effects despite the limited number of investigations, the assumption that PLA represents a benign alternative to conventional plastics warrants careful scrutiny [253,254]. Without comprehensive evaluation of their persistence, degradation products, trophic transfer, and long-term ecological impacts, it remains premature to conclude that PLA MPs and NPs are inherently safer than conventional petroleum-based plastics.

## 7. Conclusions

Despite the increased attention given to plastic pollution in the last decade, evidence suggests that the problem is worsening [49]. By 2060, global plastic production and use are projected to triple from the current levels of approximately 359 million tonnes per year [4,5,46]. Without substantial investment in waste management infrastructure, the accelerating accumulation of plastic waste will continue to pose serious environmental risks and threaten aquatic organisms. There is a pressing need to expedite the development of environmentally friendly, nontoxic bioplastics to reduce the ecological risks associated with conventional plastics. Collectively, the evidence reviewed here indicates that plastic exposure can induce profound and multifaceted effects on the nervous system of aquatic organisms, encompassing OS, accumulation, disrupted neuron morphology and neurotransmitter release/regulation, and downstream impacts on gene regulation, physiology, and behavior. Disruption of appropriate behavioral responses to environmental stimuli, such as predator recognition and avoidance, can profoundly alter population dynamics and propagate negative effects throughout aquatic food webs. Collectively, the evidence synthesized in this review underscores an urgent need for intensified research and decisive action to mitigate the toxicological impacts of MP and NP exposure, as these effects pose a significant threat to ecosystem stability and trophic interactions. Although strategies have been developed to mitigate MP pollution, no established methods currently exist for addressing NP contamination, underscoring the urgent need for additional research and resources.

## Data Availability

The original contributions presented in this study are included in the article/Supplementary Material. Further inquiries can be directed to the corresponding author.

## Supplementary Materials

The following supporting information can be downloaded at: https://www.mdpi.com/article/10.3390/ani16060941/s1, Supplemental Materials include five tables (Tables S1–S5) that provide additional data and supporting information referenced in the main text. Table S1. Oxidative stress (OS) and inflammation responses witnessed in aquatic organisms following differential acute and chronic exposures to MP and NPs. These include OS markers and inflammation. * the same paper covers both invertebrates and vertebrates. Table S2. Cellular and molecular neurotoxicity changes witnessed in aquatic organisms following acute and chronic exposures to MP and NPs. These include changes in tissue morphology, DNA damage, and accumulation. Table S3. Summary of neurochemical disruptions, including altered neurotransmitter release and degradative or hydrolytic enzyme responses, in aquatic organisms exposed acutely or chronically to MP and NP. Table S4. Genetic changes witnessed in aquatic organisms following acute and chronic exposures to MP and NPs. These gene changes include neuronal growth, apoptosis, stress, and genes regulating neurotransmitters. Table S5. Behavioral alterations in aquatic invertebrates and vertebrates following acute and chronic exposure to MPs and NPs.

## Abbreviations

5-HT	Serotonin
8-OHdG	8-hydroxy-2′-deoxyguanosine
ACh	Acetylcholine
AChE	Acetylcholinesterase
ACP	Acid phosphatase
ACR	Acrylamide
ALK	Alkaline phosphatase
ALT	Alanine aminotransferase
BBB	Blood–brain barrier
BChE	Butirylcholinesterase
BFCOD	Benzyloxy-4-trifluoromethylcoumarin-O-debenzyloxylase
BioMPs	Biomicroplastics
BPA	Bisphenol A
CAT	Catalase
CAT-L	Catalase (liver)
CbE	Carboxylesterase
ChAT	Choline acetyltransferase
ChE	Cholinesterase
CIP	Ciprofloxacin
CNS	Central nervous system
CYP450	Cytochrome P450
DA	Dopamine
dpf	Days post-fertilization
DOPA	Levodopa
EROD	7-Ethoxyresorufin-O-deethylase
EMPs	Environmental microplastics
ENV	Environmental
ENR	Enrofloxacin
FI	Fluorescence intensity
GABA	Gamma-Aminobutyric Acid
GFAP	Glial fibrillary acidic protein
GFP	Green fluorescent protein
GPx	Glutathione peroxidase
GR	Glutathione reductase
GSSG	Oxidized states of glutathione levels
GSH	Reduced glutathione
GSH-PX	Glutathione peroxidase
GST	Glutathione-S-transferase
HDPE	High-density polyethylene
hpf	Hours post-fertilization
LDPE	Low-density polyethylene
LPO	Lipid peroxidation
LYZ	Lysozyme
MAO	Monoamine oxidase
MDA	Malondialdehyde
MEHP	Mono-(2-ethylhexyl) phthalate
MET	Metolachlor
MP	Microplastic
MT	Metallothionein
NE	Norepinephrine
NEGR	Neuronal Growth Regulator
NO	Nitric oxide
NP	Nanoplastic
NPY	Neuropeptide Y
OS	Oxidative stress
OTC	oxytetracycline
p.s.u	Practical salinity unit
PA	Polyamide
PAA	Polyacrylic
PACAP	Pituitary Adenylate Cyclase-Activating Polypeptide
PAH	Polyaromatic hydrocarbons
PAN	Polyacrylonitrile
PCNA	Proliferating cell nuclear antigen
PE	Polyethylene
PEI	Polyethyleneimine
PES	Polyester
PET	Polyethylene terephthalate
PEVA	Polyethylene vinyl acetate
PFOA	Perfluorooctanoic acid
PG	Pyrogallol
PHX	Phenoloxidase
PLA	Polylactic acid
PMF	Plastic microfibers
PMMA	Polymethyl methacrylate
PP	Polypropylene
PRP	Propranolol
PS	Polystyrene
PS-COOH	Polystyrene Anionic Carboxyl
PS-NH_2_	Polystyrene Cationic Amino
PVC	Polyvinyl chloride
RF	Reticular formation
ROS	Reactive oxygen species
se-GPX	Selenium-dependent glutathione peroxidase
SMX	Sulfamethoxazole
SOD	Superoxide dismutase
TAC	Total antioxidant capacity
TCPP	tris(1-chloro-2-propyl) phosphate
TCS	Triclosan
TEM	Transmission electron microscopy
TF	Thifluzamide
TMX	Thiamethoxam
TNF-α	Tumor Necrosis Factor-alpha
Trp	Tryptophan
Tyr	Tyrosine
