# Supplementary material for: Neurophysiological and Behavioral Effects of Micro- and Nanoplastics in Aquatic Organisms
Source: Animals (Basel). 2026 Mar 17;16(6):941. doi: 10.3390/ani16060941 (PMC13023340; doi:10.3390/ani16060941)
Supplement: Supplementary file 1 [file animals-16-00941-s001.zip › animals-4149551-supplementary.pdf]

SUPPLEMENT

The following tables summarize findings from studies investigating the effects of microplastics (MPs) and nanoplastics (NPs) on aquatic invertebrates and vertebrates, including several studies that examined co-exposures to MPs or NPs with other substances. Specifically, Table S1 presents outcomes related to oxidative stress and inflammation, Table S2 details cellular and molecular neurotoxicity, Table S3 summarizes neurochemical disruptions, Table S4 reports genetic changes, and Table S5 describes behavioral modifications.

Table S1. Oxidative stress (OS) and inflammation responses witnessed in aquatic organisms following differential acute and chronic exposures to MP and NPs. These include OS markers and inflammation. * the same paper covers both invertebrates and vertebrates

Species	Plastic/size	Exposure Dose(s)	Exposure Method	Physiological Effect(s)	Reference
**Invertebrates**					
Marine rotifers (*Brachionus plicatilis*) and Brine shrimp (*Artemia salina*)	Polylactic acid (PLA) NPs (250 nm)	0.1, 1, and 100 mg/L	In exposure media for 24 h	• No changes in catalase (CAT) activity for Marine rotifers or Brine Shrimp	Mustapha et al. [1]*
Polychaete (*Hediste diversicolor*)	PET-MPs (125 µm to 1 mm) with and without the antibiotic ciprofloxacin (CIP) and industrial effluents	MPs (0.032 g/L and 0.054 g/L)<br>CIP (130 ng/L and 1300 ng/L)<br>Industrial effluents (3%)	In sea water for 28 days	• CAT activity increased in CIP- and MP-exposed organisms<br>• Superoxide dismutase (SOD) activity was affected by treatments<br>• SOD activity increased in organisms exposed to CIP alone at both concentrations<br>• Low CIP combined with MPs maintained high SOD activity, with 3% effluent + + low CIP + low MP differing significantly from control, effluent, and 3% effluent + + high CIP + low MP groups<br>• High CIP combined with MPs resulted in a smaller increase in SOD activity than CIP alone<br>• Higher CIP concentrations led to lower SOD activity than lower CIP concentrations, an effect exacerbated by high MP density<br>• Combined exposure to CIP and MPs markedly reduced both Selenium-dependent glutathione peroxidase (Se-GPx) and total Glutathione peroxidase (.GPx) activities	Araújo et al. [2]
Brine shrimp nauplii and juveniles (*Artemia franciscana*)	Polymer MPs (1–5 µm)	0.4 and 1.6 mg/mL	In water for 2 and 5 days	• Nauplii: increases in Carboxylesterase (CbE), Glutathione reductase (GR), and Glutathione-S-transferase (GST) occurred only at the higher concentration, but CAT remained unchanged (2 days)<br>• Juveniles: After 5 days at 1.6 mg/mL, CbE activities were significantly reduced, while CAT activity was increased	Peixoto et al. [3]
Water flea (*Daphnia magna*)	Tire wear particles (TWP) MPs (0.7-70 µm) and leachate extraction from TWP for 30 and 60 min (E-30 and E-120)	TWP, 30-E, and 120-E (50, 100, 200, 400, 600, and 800 mg/L each)	In culture media for 24 and 48 h	• Antioxidant defenses were suppressed, with significant inhibition of SOD and Total antioxidant capacity (TAC) at higher exposures<br>• Malondialdehyde (MDA) levels increased following exposure to high concentrations of particles and leachates	Liu et al. [4]
Water flea (*Simocephalus vetulus*)	Polystyrene (PS)-NPs (500 nm)	1, 5 and 10 mg/L	In water for 14 days	• NP ingestion induced OS in the water flea	Zhu et al. [5]
Mysid (*Neomysis awatschensis*)	PS-MP (1 µm) and As, Cd, Cu, Pb,	1. Metals,<br>2. Metals with fresh MPs (10 particles mL),	In water for 30 and 60 days	MDA<br>• Juveniles: MDA levels were elevated under metal + MPs	Eom et al. [6]

	and Zn (1/10th of LC_{50})	3. Metals premixed with MPs for 30 days, and<br>4. Metals premixed with MPs for 60 days		exposure across feeding (F), 30 d, and 60 d treatments, with the strongest increases observed for Pb, Cd, Cu, and Zn at longer durations, indicating enhanced oxidative damage compared with metals alone<br>• Adults: MDA content was also increased under metal + MPs treatments, particularly after 30 d and 60 d exposure, with broad responses across As, Cd, Cu, Pb, and Zn, and the highest values generally at 60 d<br>GSH<br>• Juveniles: Reduced glutathione (GSH) content was reduced under metal + MPs exposure at 30 d (Cd, Zn) and 60 d (As, Cd, Pb, Zn), suggesting depletion of antioxidant reserves<br>• Adults: In contrast, GSH levels were increased under metal + MPs (F) treatment with Cu, Pb, and Zn, indicating an inducible antioxidant response at early exposure<br>SOD<br>• Juveniles: SOD activity showed a mixed response, increased mainly under metal + MPs (F) and Cu treatments, but markedly decreased at 30 d and 60 d for several metals (As, Cd, Pb, Zn), reflecting potential enzymatic inhibition with prolonged exposure<br>• Adults: SOD activity was strongly induced under metal + MPs (F) for all metals tested, but significantly suppressed at 30 d and 60 d for most metals, except for a transient increase with Cu at 60 d<br>CAT<br>• Juveniles: CAT activity was reduced under metal + MPs exposure at 30 d and 60 d for Cd, Pb, Zn, and Cu, while an increase was observed only under metal + MPs (F) with Cu<br>• Adults: CAT activity was generally enhanced under metal + MPs (F) and during longer exposure with Cu, As, Pb, and Zn, but decreased under metal + MPs (30 d) with Pb	
Freshwater shrimp (*Caridina fossarum*)	Polyethylene (PE)-MPs (15–25 µm) with and without lead acetate	PE-MPs (500, and 1000 µg/L)<br>Lead (2.5, and 5 mg/L)	In water for 15 days	• TAC increased in Pb-exposed shrimp but was unaffected by PE-MPs alone<br>• MDA levels increased with Pb, PE-MPs, and especially under co-exposure, reaching the highest values at high PE-MPs combined with 2.5–5 mg/L Pb	Gholamhosseini et al. [7]
South American native shrimp (*Palaemon argentines*)	PLA-MPs (minor side: 116.8 ± 27.8 µm; major side: 175.8 ± 44.5 µm; mean area: 0.017 ± 0.007 mm^2) with and without metolachlor (MET)	7.5 µg/L MET ($MET_{[7.5]}$)<br>5 mg/L PLA-MPs ($MPs_{[5]}$)<br>7.5 µg/L MET + 5 mg/L PLA-MPs ($MET_{[7.5]}$+$MPs_{[5]}$)	In water for 7 days	• Exposure to $MET_{[7.5]}$ inhibited CAT activity in both the cephalothorax and abdomen<br>• PLA-MPs ($MPs_{[5]}$) and the co-exposure ($MET_{[7.5]}$+$MPs_{[5]}$) did not alter CAT activity, suggesting mitigation of MET toxicity by MPs adsorption<br>• GST activity:<br>• Positive trend in $MET_{[7.5]}$ and $MPs_{[5]}$ groups<br>• Co-exposure treatment induced GST activity, indicating enhanced detoxification response	Bertrand et al. [8]

Red Swamp Crayfish (*Procambarus clarkii*)	MPs (size and type not stated) with and without PG	PG (10 mg/L),<br>MPs (100 mg/L), and Combination (10 mg/L PG + 100 mg/L MPs)	In water for 15 days	• Decreased immune parameters:<br>• Serum lysozyme (LYZ) activity showed changes in the PG, MPs, and PG + MPs groups<br>• LYZ activity was highest in the MPs-only group but decreased in the combined PG + MPs treatment<br>• Phenoloxidase (PHX) activity declined under PG, MPs, and PG + MPs exposures, with the strongest reduction observed in the PG-only group<br>• Acid phosphatase (ACP) activity decreased in the PG-exposed group<br>• ACP responses in the MPs-only and PG + MPs treatments were comparable and differed from the control<br>• No significant differences were detected between MPs and PG + MPs treatments for PHX or ACP	Hamed et al. [9]
Aquatic midge (*Chironomus riparius*)	PS; 1 µm	22,400, 112,000, and 224,000 plastics/kg sediments dry weight	In soil for 56 days (2 generations; 28 days for the 1st generation and other 28 days for the 2nd generation)	• No difference in reactive oxygen species (ROS), CAT, SOD, and GST (2nd generation)	Sbarberi et al. [10]
Freshwater bivalves (*Corbicula fluminea*)	PS-MPs (6 µm) and PS-NPs (80 nm) with and without the antibiotic CIP	PS-MPs and PS-NPs (10 µg/g dw);<br>CIP (low; 0.5 µg/g and high; 5 µg/g, and 50 µg/g dry weight (dw))	In sediment for 10 days	• SOD activity in digestive glands increased at low CIP (0.5 µg/g) and then decreased at high CIP (50 µg/g) in both single and co-treatments<br>• CAT activity decreased at low CIP and recovered/ increased at higher concentrations, showing a complementary relationship with SOD<br>• Co-exposure with PS-NPs caused a greater SOD decrease than PS-MPs at the highest CIP concentration, while CAT recovery was similar between PS-NP and PS-MP co-treatments<br>• GSH increased at low contaminant levels (10 µg/g PS, 0.5 µg/g CIP, and co-treatments)<br>• GSH decreased at high CIP concentrations (50 µg/g), indicating impaired antioxidant defense<br>• PS-NP co-exposure aggravated GSH depletion more than PS-MP co-exposure at high CIP<br>• GSH-related enzymes (Glutathione peroxidase (GSH-Px) and GR):<br>• GSH-Px activity increased at low contaminant levels and decreased at high CIP, consistent with GSH content.<br>• GR activity showed an opposite trend, increasing at high CIP to regenerate GSH from Oxidized states of glutathione levels (GSSG)<br>• Presence of PS-NP/MP had no significant effect on GR at high CIP concentration<br>• Lipid peroxidation (LPO):<br>• MDA content significantly increased at higher CIP (5 and 50 µg/g) and in co-exposures<br>• PS-MP co-exposure significantly reduced MDA levels compared to CIP alone, indicating a mitigation of peroxidation damage<br>• PS-NP co-exposure slightly reduced MDA but not significantly	Guo et al. [11]

				• Indicates ROS levels exceeded antioxidant elimination capacity at high CIP, causing oxidative damage<br>• GST activity trend was similar to GSH suggesting that GST plays a role in detoxification and protection against OS under single and co-exposure conditions	
Freshwater bivalves (*Corbicula fluminea*)	Commercial PS-NPs (200 nm) and NPs generated from macro-sized plastics collected in the field (Environmental (ENV)-NPs; 235 ± 70 nm)	Microalgae were contaminated for 48 h with 0.008, 10 and 100 µg/L PS-NPs and ENV-NPs	Bivalves were then fed with the contaminated algae every 48 h for 21 days	Gill CAT activity – 7 days:<br>• CAT activity was increased in organisms exposed to PS 10, ENV NP 0.008, and ENV NP 100 compared with controls<br>Gill CAT activity – 21 days:<br>• Slightly elevated CAT activity was observed in organisms exposed to PS 0.008, ENV NP 10, and ENV NP 100 relative to controls<br>Visceral mass CAT activity – 7 days:<br>• CAT activity was reduced in organisms exposed to PS 100 compared with the control group<br>Visceral mass CAT activity – 21 days:<br>• Exposure to PS 0.008, ENV NP 0.008, and ENV NP 10 resulted in increased CAT activity compared with controls	Latchere et al. [12]
Marine clam (*Donax trunculus*)	PE-MPs (150–250 µm) with and without Gadolinium (Gd)	MPs (0.1 or 100 µg/L)<br>Gd (500 µg/L)<br>mixture of contaminants (0.1 MPs + 500 Gd µg/L; 100 MPs + 500 Gd µg/L)	In water for 14 days (examined every 7 days)	Antioxidant responses (SOD, TAC):<br>• SOD activity decreased in both MPs-only treatments during weeks 1 and 2<br>• SOD activity increased in the 500 Gd µg/L treatment and in both mixture (MPs + Gd) treatments at both time points<br>• A temporal increase in SOD activity was observed in the 0.1 MPs µg/L + 500 Gd µg/L treatment, with higher activity in week 2 than week 1<br>• TAC decreased in both MPs-only treatments in week 1 and remained significantly reduced after week 2<br>• TAC levels were significantly higher in both mixture treatments after week 2<br>• A between-week difference in TAC was detected in the 100 MPs + 500 Gd µg/L treatment, with higher values in week 2<br>Biotransformation enzymes (CbEs, GSTs):<br>• CbE activity increased in both MPs-only and 500 Gd µg/L treatments after weeks 1 and 2<br>• GST activity decreased in the 100 MPs µg/L treatment, whereas increases were observed in the 500 Gd µg/L and both mixture treatments during week 1<br>• The same GST response pattern persisted after week 2<br>• Compared to week 1, GST activity was lower in week 2 in the 100 MPs µg/L, 500 Gd µg/L, and 0.1 MPs + 500 Gd µg/L treatments<br>Cellular damage (LPO):<br>• LPO levels decreased in week 1 in clams exposed to 0.1 MPs µg/L and 500 Gd µg/L<br>• A similar decrease in LPO was observed after week 2 in the same treatments	Secco et al. [13]

				• An increase in LPO was detected in the 0.1 MPs μg/L + 500 Gd μg/L treatment after week 2	
Blue mussels (*Mytilus edulis*)	Pristine PE-MPs (27 – 45 μm) and Aged PE-MPs, with and without Polyaromatic Hydrocarbons (PAHs) (phenanthrene and fluoranthene)	Pristine MPs (10 μg/L)<br>Phe (1 μg/L)<br>Fluo (0.5 μg/L)	In water for 8 days	• CAT activity was elevated in the digestive gland of mussels exposed to aged MPs with PAHs (AMP-PAH)<br>• SOD activity decreased in the digestive gland under aged MP exposure and in the mantle under AMP-PAH exposure<br>• GST activity was highest in the digestive gland and increased in the MP group compared with AMP and MP-PAH treatments	Moncrieffe et al. [14]
Mediterranean mussel larvae (*Mytilus galloprovincialis*)	Environmental MPs (EMPs) consisting of PE, PET, polypropylene (PP), polyethylene vinyl acetate (PEVA), High-density polyethylene (HDPE), and low-density polyethylene (LDPE) (sizes 1.22 to 30 μm) in control and high salinity salt water	EMPs (1, 10, 50, and 100 μg/L)<br>Control salinity (33 p.s.u)<br>High salinity (37 p.s.u)	In salt water (33 or 37 p.s.u) for 48 h	• Activities of CAT and GST were affected by EMP exposure, salinity, and their combined effects in mussel D-larvae<br>• At physiological salinity (33 p.s.u.), CAT activity increased significantly from 10 μg/L EMPs, while GST activity increased only at the highest concentration (100 μg/L)<br>• Elevated salinity alone (37 p.s.u.) significantly increased CAT and GST activities compared with 33 p.s.u.<br>• Under combined EMP and high-salinity exposure (37 p.s.u.), CAT and GST activities initially increased, peaking at 10 μg/L EMPs, then declined at 50 and 100 μg/L	Boukadida et al. [15]
Mediterranean mussel (*Mytilus galloprovincialis*)	PET-microfibers (MF; 100 μm)	0.0005, 0.1, 1, 10, and 100 mg/L	For 32 days	• PET-MFs induce OS as CAT activity increased in the digestive gland, while no change was observed in the gills<br>• SOD activity increased in a dose-dependent manner in both the digestive gland and gills following PET MF exposure	Choi et al [16]
Mediterranean mussel (*Mytilus galloprovincialis*)	HDPE-MP (≤ 22 μm) and microalgae (MA; 3 and 8 μm) with and without chlorpyrifos (CPF)	MP (1.5 mg/L)<br>CPF (7.6 μg/L)<br>MP-CPF<br>MA-CPF	In sea water for able7 and 21 days	7 days:<br>• Increased GST and SOD activities in the digestive gland of mussels exposed to CPF, MA-CPF, and MP-CPF<br>• Elevated GST activity in gills of CPF- and MA-CPF–exposed mussels<br>• Reduced GR activity in the digestive gland of MP-CPF–exposed mussels<br>21 days:<br>• Decreased GR activity in the digestive gland of mussels exposed to dissolved CPF<br>• Reduced SOD and GR activities in the digestive gland of MA-CPF–exposed mussels<br>• Reduced GR activity in digestive gland and increased GP activity in gills of MP-CPF–exposed	Fernández et al. [17]
Mediterranean mussels (*Mytilus galloprovincialis*)	PS, PP, PET (80 - 240 μm) alone and co-exposed with Cadmium chloride ($CdCl_2$)	2 mg/L (~25,700 particles/L) plastics individually and with $CdCl_2$ (30 μg/L Cd^{2+})	In water for 7 days	• Tissues from mussels exposed to a combination of PET and Cd exhibited lower levels of Cd accumulation<br>• PP + Cd and PET+Cd have lower MP quantities<br>• Increased ROS in Cd-treated or in combination with MPs (PS + Cd and PP + Cd, not PET+Cd)<br>• Decreased GSH in all treated groups<br>• Increase LPO, Alanine aminotransferase (ALT), and lactate dehydrogenase in the mantle<br>• Decreased CAT, SOD, GPx, GR, and GST in all groups	Tuncelli et al. [18]
Mussels (*Mytilus galloprovincialis*)	Leachate obtained from abandoned or lost fishing nets	1, 10, and 100 mg/L	In water for 0, 3, 7, 14, 21, and 28 days of exposure	• SOD activity: increased in gills after 21 days at 1 mg/L and earlier (after 3 days) at 10 mg/L, while in the	Vilke et al. [19]

	and cables of seven color groups (Contained PP, PE, and polyamide (PA) (various sizes)). Cu, Fe, Mn, Ni, Pb, and Zn, and other organic compounds were also detected in the leachate			digestive gland SOD increased after 21 days at both concentrations and remained elevated at 28 days at 10 mg/L<br>• CAT activity: decreased in gills after 28 days at 1 mg/L, whereas in the digestive gland CAT activity increased at 1 mg/L after 21 and 28 days, increased earlier (3 days) and again at 28 days at 10 mg/L, and increased after 3 days at 100 mg/L<br>• GPx activity: increased in gills at 10 mg/L after 21 and 28 days and at 100 mg/L from 14 days through the end of exposure, while in the digestive gland GPx activity increased at 10 mg/L after 21 days and at 100 mg/L after 3 and 21 days.<br>• GR activity: increased in gills throughout exposure at 10 mg/L (except day 14) and at 100 mg/L between days 7 and 14, while in the digestive gland, GR activity increased only at 10 mg/L after 21 days<br>• GST activity: GST activity decreased in gills after 14 days at 10 mg/L but increased after 28 days, and increased at 100 mg/L after 7 days; in the digestive gland, GST activity increased at 1 mg/L after 14 and 28 days and at 10 mg/L after 21 days, while decreasing at 100 mg/L from 21 days to 28 days<br>• LPO in the digestive gland increased after 14 and 21 days for the 10 mg/L treatment and after 3, 14, and 21 days for the 100 mg/L treatment. At the end of the 28-day experiment, LPO levels were similar to those of the control group	
Marine mussels (*Mytilus coruscus*)	PS-MPs (2.0 µm)	Four concentration exposures (0, 10, 10^4 and 10^6 particles/L)	In water for 14 days followed by a 7-day recovery period	• During PS-MP exposure, ROS levels increased along with LPO markers (MDA and LPO)<br>• Increases in ROS and LPO were observed only in the high-concentration groups (10^4 and 10^6 particles/L)<br>• No OS was detected in the low-concentration group (10 particles/L)<br>• After a recovery period in clean water, the elevated ROS and LPO levels returned to baseline<br>• ACP and alkaline phosphatase (ALK) activities increased suggesting mussels mount an immune response to resist PS-MPs, returning to normal following 7 days in clean water	Huang et al. [20]
Marine mussels (*Mytilus coruscus*)	PS-MPs (1 µm)<br>PS-NPs (100 nm)	20 mg/L	In seawater for 2 days	• Exposure to MP and NPs, mussels showed significantly increased ROS, TAC, and GSH activities in digestive tissues<br>• ROS and TAC levels were higher in mussels exposed to NPs compared with those exposed to MPs, suggesting stronger OS responses to NPs	Qi et al. [21]
Marine mussels (*Mytilus coruscus*)	PLA MPs (5 µm, irregular fragments) with and without tris(1-chloro-2-propyl) phosphate (TCPP) an organophosphate flame retardants	PLA MPs (10^2 and 10^6 particles/L)<br>TCPP (0.5 and 50 µg/L)	In seawater for 14 days	• CAT activity increased in groups exposed to low concentrations of PLA or TCPP<br>• SOD activity increased significantly in high-TCPP + PLA exposure compared with PLA alone<br>• MDA was elevated in the low-TCPP + PLA group<br>• AKP activity increased in low-TCPP, PLA, and combined low-dose groups	Zhong et al. [22]

				but decreased under high-dose combined exposures<br>• ACP activity increased with low concentrations of either TCPP or PLA<br>• LYZ activity increased significantly in mixed treatments involving high TCPP	
Marine mussels (*Mytilus coruscus*)	PS-MPs (90 - 110 µm with and without thifluzamide (TF) and enrofloxacin (ENR)	PS-MPs (400 µg/L)<br>TF (0.06 µg/L)<br>ENR (0.06 µg/L)	In seawater for 4 weeks	• CAT activity increased in all exposure groups<br>• GST activity significantly increased in TF, ENR, PS, PS+TF, and TF+ENR groups<br>• MDA content increased in PS and PS+ENR groups	Zhang et al. [23]
Black sea urchin larva (plutei) *Arbacia lixula*	PS-MPs (1 and 5 µm) alone and conjugated with Bisphenol A (BPA)	PS-MPs (10 µg/mL)<br>BPA (5 and 25 µM)	In exposure medium for 48 hours post fertilization (hpf)	• GST activity increased in plutei exposed to both 1 and 5 µm PS MPs and to 5 µM BPA adsorbed onto both PS MP sizes<br>• GST activity was decreased in plutei exposed to BPA at 5 and 25 µM adsorbed onto 1 and 5 µm PS MPs<br>• CAT activity showed no change in plutei exposed to 1 µm PS MPs alone<br>• A decrease in CAT activity was observed in samples exposed to 5 µM BPA and to both concentrations of BPA adsorbed onto 1 and 5 µm PS MPs<br>• CAT activity was increased in plutei exposed to 5 µm PS MPs	Eliso et al. [24]
**Vertebrates**					
Tadpoles (*Xenopus tropicalis*)	Triclosan (TCS) adsorbed on PE-MPs (38.8 µm) and aged PE-MPs (36.6 µm)	PE-MPs and aged PE-MPs (1 mg/L)<br>TCS (1 µg/L)	In water for 7 days	• Exposure to PE/aged PE-MPs and TCS increased SOD and CAT activities, with the highest effects in the aged PE+TCS group<br>• MDA levels indicated that TCS drove OS, and combined exposure caused synergistic toxicity	Lin et al. [25]
Tadpoles (*Rana nigromaculata)*	PS-MPs 0.1 µm (MP1), 1 µm (MP2), and 10 µm (MP3)) with and without levofloxacin (LVFX)	LVFX (1.00 µg/L), LVFX (1.00 µg/L) with PS-MPs (MP1; 0.10 mg/L), LVFX (1.00 µg/L) with MP2 (0.10 mg/L), and LVFX (1.00 µg/L) with MP3 (0.10 mg/L)	In water for 45 days	• The LVFX-MP3 group exhibited the highest OS levels among treatments, with markedly elevated ROS, SOD, GSH-PX, and CAT<br>• Overall intestinal OS followed the order:<br>LVFX-MP3 ≥ LVFX-MP2 ≥ LVFX-MP1 ≥ LVFX	Zhang et al. [26]
Zebrafish embryos (*Danio rerio*)	PS-NPs (20 nm)	3 nL of PNP stock solution was injected into the yolk sac of 4 hpf embryo (final concentration (~270 ppm)	120 h and changes recorded at 24, 48, 72, 96 and 120 hpf	• NP-injected larvae exhibited pronounced ROS accumulation at 120 hpf throughout the body, with particularly strong signals in the head region	Sökmen et al. [27]
Zebrafish embryos (*Danio rerio*)	PS-NPs (100 nm) with and without Avobenzone (AVO)	NPs (10 µg/L)<br>AVO (10 µg/L)	In media for 144 h followed by a 72-h recovery in dH_2O	• CAT and SOD activities were altered in a time- and treatment-dependent manner, indicating OS<br>• After recovery, CAT activity normalized, while SOD activity remained elevated, suggesting persistent OS	Liu et al. [28]
Zebrafish embryos (*Danio rerio*)	Plastic microfibers (PMF) from facial mask (surgical and N95) leachate;<br>• Surgical – length 112.7 ± 57.12 µm and width of 4.28 ± 1.16 µm (PP fibers plus aluminum (Al) and calcium (Ca))<br>• N95 – length 7.89 ± 11.69 µm and width of 2.15 ± 0.50 µm	1000, 10,000, and 100,000 particle/L	In exposure media for 144 h	Zebrafish larvae exposed to PMFs from both surgical and N95 masks showed higher ROS levels compared to controls<br>• Whole body: +59.72% (surgical), +57.42% (N95)<br>• Head and trunk: +62.77% (surgical), +61.53% (N95)<br>• Tail: +53.30% (surgical), +55.43% (N95)<br>• Liver region: highest increase, +80.21% (surgical) and +81.01% (N95)	Qualhato et al. [29]

	(PET fibers and Al, Ca, silicon (Si), chlorine (Cl), and potassium (K))				
Zebrafish embryos (*Danio rerio*)	PS-NPs (20 nm)	• 3 nL (microinjection)<br>1 ppm (waterborne)	Injected into the yolk sac of embryos 3 (nL) or in media (1ppm) for 5 days, then 5 days in media without NPs and examined after 6 months after 6 months	• Decreased SOD, GPx, and OX, indicates a compromised ability to neutralize ROS<br>• Increased monoamine oxidase (MAO) in injected embryos and CAT in waterborne treatment<br>• Increased 8-hydroxy-2'-deoxyguanosine (8-OHdG), a biomarker for OS and DNA damage, expression in key brain regions	Kankaynar et al. [30]
Zebrafish embryos (*Danio rerio*)	PLA NPs (250 nm)	0.1, 1, and 100 mg/L	In exposure media for 120 hpf	• No changes in CAT activity for zebrafish	Mustapha et al. [1]*
Zebrafish embryos (*Danio rerio*)	PS-NPs (0.4-0.6 µm) with and without fluoxetine (FLX)	PS-NPs (25, 100 particles/L)<br>FLX (5, 40 ng/L)	In water for 24 and 96 hpf	• SOD and CAT activities increased, and biomarkers of LPO, protein carbonylation, and hydroperoxides were elevated, particularly in the 40 ng/L FLX + 100 particles/L MPs mixture, indicating dose- and combination-dependent OS	Orozco-Hernández et al. [31]
Zebrafish embryos (*Danio rerio*)	PS-MPs (5 µm) and PS-NPs (100 nm) with and without silver nanoparticles (Ag-NPs; 5 nm)	PS MPs/NPs (200 µg/L) and AgNPs (10 µg/L)	In media for ~120hpf	• AgNP-containing treatments increased ROS and MDA levels and decreased GSH<br>• PS M/NPs alone did not significantly induce OS<br>• Macrophage and innate immune cell recruitment increased, particularly in NP groups	Song et al. [32]
Zebrafish embryos (*Danio rerio*)	PS-MPs (5 µm) and PS-NPs (60 nm)	0.05–50 mg/L	In water for 7days post fertilization (dpf)	• Both PS-MPs and PS-NPs induced ROS accumulation in larval head and abdominal regions<br>• Elevated ROS levels were observed in all treatment groups (0.5–50 mg/L)<br>• MDA increased in the 0.5 mg/L PS-NP group and in the 0.5 and 5 mg/L PS-MP groups	Li et al. [33]
Zebrafish larvae (*Danio rerio*)	PS-NPs (80 nm) combined with 2,2 ‘,4,4 ‘-tetrabromodiphenyl ether (BDE-47)	PS-NPs (0.05, 0.1, 1, 5, 10 mg/L), BDE-47 (0.1 and 10 µg/L)	In water for 120 hpf	• PS-NPs + BDE-47 co-exposure increased the hepatotoxicity<br>• Co-exposure significantly exacerbated ROS production	Wang et al. [34]
Zebrafish larvae (*Danio rerio*)	Aged PS-MPs (1 µm) and Thiamethoxam (TMX)	Aged PS-MPs (1 µg/L)<br>TMX (1.5 µg/L)	In water for 120 hpf	• All treatments significantly decreased SOD, CAT, and GST activities and increased MDA levels<br>• Co-exposure produced the highest MDA content, indicating exacerbated LPO and compromised antioxidant defenses	Sun et al. [35]
Zebrafish larvae (*Danio rerio*)	PS-NPs (80 nm) combined with and without Mono-(2-ethylhexyl) phthalate (MEHP)	PS-NPs (10, 25 and 50 µg/mL) and MEHP (10^{-6} and 10^{-5} M)	In embryo media for 24 hpf and 7 dpf	• OS: ROS levels increased in zebrafish larvae in a PS-NP concentration–dependent manner<br>• Synergistic effect with MEHP: Co-exposure to PS-NPs and MEHP further amplified ROS production	Liu et al. [36]
Zebrafish larvae (*Danio rerio*)	PS-NPs (250 nm) with and without methylmercury (MeHg)	1000 µg/L NPs, 1 µg/L MeHg (MeHg1), 10 µg/L MeHg (MeHg10), or their respective combinations (Mix1 and Mix10)	In water for 30 days	• Immunohistochemical analysis revealed strong 8-hydroxy-2'-deoxyguanosine (8-OHdG) immunopositivity in neuronal cells of NP-injected larvae<br>• The majority of 8-OHdG signals co-localized with NP fluorescence, suggesting a spatial association between NP presence and DNA damage	Oger et al. [37]
Zebrafish juveniles (*Danio rerio*)	PET-MPs (0.15 mm) with and without abamectin (ABM)	PET-MPs (5 mg/L and 10 mg/L)<br>ABM (0.006 mg/L)	In water for 96 h	• Whole body analysis showed a decrease in CAT activity following the ABM alone and ABM + MP treatments<br>• GST activity was increased in MP-only treatments	Kazemi et al. [38]
Zebrafish juveniles (*Danio rerio*)	PS-MPs and PS-NPs with and	PS-MPs (0.1 µg/mL)<br>PS-NPs (0.1 µg/mL)	In water for 7 and 28 days	• Combined exposures altered CAT and SOD responses in an organ- and	Wu et al. [39]

	without perfluorooctanoic acid (PFOA)	PFOA (200 ng/L)		time-dependent manner, with early synergistic OS followed by impaired antioxidant capacity during prolonged exposure<br>• GST activity responses differed by particle size and duration<br>• MPs and NPs initially elevated GST when combined with PFOA, but long-term exposure reduced GST activity, suggesting antioxidant exhaustion and modulation of PFOA bioavailability via adsorption<br>• PFOA strongly increased MDA levels in all organs, confirming oxidative damage<br>• MPs + PFOA caused the highest intestinal LPO, indicating enhanced gut toxicity driven by mechanical injury and ROS generation	
Zebrafish larvae and adults (*Danio rerio*)	Schwarzbach River water samples containing PE (50%), PP (25%), PS (15%), and polyvinyl chloride (PVC) (10%) and sorbed contaminants (up to 94 different compounds)	100 mg/L (except larval behavior tests 5 mg/L)	In river water for 24, 48, 72, and 96 hpf and 21 days for adults	Schwarzbach water exposure:<br>• Strongly induced 7-Ethoxyresorufin-O-deethylase (EROD)/ cytochrome P450 (CYP450) activity at both sampling times	Hanslik et al. [40]
Zebrafish (*Danio rerio*)	PS-NPs (70 nm)	PS-NPs (0.5 and 1.5 ppm)	In water for 7 days	• ROS levels were increased in fish exposed to high concentrations of PS-NPs<br>• Tumor Necrosis Factor-alpha (TNF-α), CYP1A1, CYP11A1, and CYP19A1 were elevated in the 1.5 ppm PS-NPs group, indicating hepatic inflammation at higher exposure	Sarasamma et al. [41]
Zebrafish (*Danio rerio*)	Polymer MPs (1–5 μm) with and without Cu	MPs (2 mg/L)<br>$CuSO_4 \cdot 5H_2O$ (25 μg/L)	In water for 30 days	• CAT and GPx activities were significantly inhibited in Cu25 + MPs, with GPx also reduced in Cu25 alone<br>• GST activity increased in the Cu25 + MPs group<br>• LPO increased in Cu25 + MPs<br>• GSH levels increased in Cu25 alone<br>• GSH, GSSG, and GR were unchanged in the combined exposure<br>• Metallothionein (MT) levels increased in MPs and Cu25 alone but were reduced to control-like levels in Cu25 + MPs<br>• Combined Cu and MPs exposure overwhelms antioxidant defenses, impairing redox balance and causing oxidative damage, whereas single exposures elicit more effective compensatory responses	Santos et al. [42]
Zebrafish (*Danio rerio*)	PS-NPs (100 nm) with and without and arsenic (As)	PS-NPs (1 mg/L)<br>As (1 mg/L)	In water for 30 days	• Intestinal MAO, SOD, and MDA levels increased in co-exposure group and GSH was reduced<br>• Mitochondrial damage was also present	Zhang et al. [43]
Zebrafish (*Danio rerio*)	PS-MPs (2 and 25 μm) and PS-NPs (25 and 250 nm)	1 mg/L	In water for 14 days	• CYP450 concentrations in the liver were elevated in all exposure groups	Su et al. [44]
Zebrafish (*Danio rerio*)	PE MPs (13.5 μm) and NPs (70 nm) with and without PFOA	MPs and NPs (0.1 μg/mL) and PFOA (200 ng/L)<br>Exposure groups: PFOA, PE-MPs + PFOA, PE-NPs + PFOA, and PE-MPs + PE-NPs + PFOA	In water for 21 days (sampling every 7 days)	• After 21 days of exposure, CAT activity in gill tissue was increased, with combined-exposure groups showing higher CAT levels than the PFOA-only group, indicating sustained OS<br>• Intestinal antioxidant responses (CAT, GSH, and GST) in the MPs + PFOA, NPs + PFOA, and MPs +	Wang et al. [45]

				NPs + PFOA groups gradually returned to levels similar to those observed at day 7, suggesting the development of adaptive stress-response mechanisms<br>• In contrast, the MPs + NPs + PFOA co-exposure group exhibited elevated intestinal CAT activity and LPO levels, indicating excessive ROS production that exceeded antioxidant capacity and led to oxidative damage<br>• In the liver, co-exposure treatments resulted in higher CAT activity and LPO content than PFOA alone, reflecting enhanced OS and significant LPO injury<br>• Reduced SOD and GST activities and decreased GSH content in the liver of co-exposure groups indicated antioxidant depletion and suppression of detoxification pathways	
Painted comber (*Serranus scriba*)	PEVA and HDPE and other smaller size classes including PS, PA, and LDPE (3–1.2 µm and 1.2–0.45 µm) and other chemicals	Wild caught	Wild caught	• CAT and GST activities were highest in fish from the most polluted site and lowest at the least polluted site and MDA was elevated across all sites, indicating OS	Zitouni et al. [46]
Common carp (*Cyprinus carpio*)<br>Sea mullet (*Mugil cephalus)*<br>European flounder (*Platichthys flesus*)	Wild caught - MPs (150–1499 µm) dominated and polymer profiles differed among species and seasons	Wild caught in March and September	Wild caught in March and September	• Fish with high MP loads (MP-H) showed higher stress (Integrated Biomarker Response; IBR) than moderately contaminated fish (MP-M)<br>• MP-H carps showed signs of OS<br>• March also showed induction of GST, GR, and GPx activities, with LPO detected in mullet brains and flounder muscle	Martins et al. [47]
Grass carp (*Ctenopharyngodon idella*)	PS-MPs (5 µm)	1000 µg/L	In water for 7 days (28 h total, with 2-h sessions twice daily) in low, medium, and high velocity water	• Brain antioxidant and neurotoxicity biomarkers (SOD and LPO) differed among treatments<br>• SOD activity was highest in the MPs + HV group, followed by the MPs + medium velocity (MV) group<br>• LPO activity was significantly increased in the MPs + HV treatment, representing the strongest oxidative damage response, followed by MPs + MV	Rasta et al. [48]
Goldfish (*Carassius auratus*)	PP-MPs (6.37 µm) with and without oxytetracycline (OTC)	PP-MPs (100 µg/L), OTC (200 µg/L)	In water for 21 days	• MPs- or OTC-only exposure significantly elevated TAC levels in both the brain<br>• The combined exposure induced a stronger TAC response than single exposures	Zhang et al. [49]

Channel catfish (*Ictalurus punctatus*)	PS-NPs (80 nm) and BPA with and without Biochar	PS-NPs (0.5 mg/L)<br>BPA (0.2 mg/L)<br>Biochar (0.5 g/L)	In water for 7 days	• CAT and SOD in the liver and gut were elevated in PS-NP treatments<br>• MDA in the liver was also elevated	Zheng et al. [50]
African catfish (*Clarias gariepinus*)	PVC-MPs (95.41 ± 4.23 μm)	0.50%, 1.50% and 3.0% in the diet	In the diet for 45 days and depuration for 30 days	• A reduction in GPx activity was observed in fish exposed to 0.5% PVC<br>• GPx activity declined progressively with exposure time, but values after depuration were not different from those at days 30 and 45<br>• A decrease in SOD activity occurred in the 0.5% PVC group relative to controls<br>• SOD activity showed a biphasic pattern over time, with no treatment × duration interaction<br>• Brain CAT activity was reduced in the 0.5% PVC group compared with controls<br>• CAT activity declined during the 30–45-day exposure period but increased after depuration<br>• LPO levels were higher in PVC-exposed fish than in controls and increased in a duration-dependent manner<br>• LPO levels decreased at the end of the depuration period	Iheanacho and Odo [51]
Javanese Medaka Fish (*Oruzias javicus*)	PS-MPs (5 μm)	PS-MPs<br>LOW (100 μg/L; 1.46 × 10^3), MED (500 μg/L; 7.3 × 10^3), and HIGH (1000 μg/L; 1.46 × 10^4)	In salt water (14.5 ± 0.5 ppt) for 21 days	• CAT activity slightly increased in MP-LOW and decreased in MP-MED, with a significant decrease in MP-HIGH versus control<br>• SOD activity declined progressively across MP-LOW, MP-MED, and MP-HIGH groups; MP-HIGH was lower than MP-LOW and control<br>• MDA levels showed a progressive increase in MP-LOW, MP-MED, and MP-HIGH compared with control	Usman et al. [52]
Nile tilapia (*Oreochromis niloticus*)	NPs and light-induced zinc oxide NPs (ZnONPs; 220 nm)	NPs (10, 20, and 30 mM)<br>• ZnONPs (5 mg/L)<br>• 50% sewage effluent<br>• 75% sewage effluent<br>• 50% sewage treated effluent UV mediated photodegradation by ZnONPs (500 mg/L)<br>75% sewage treated effluent UV mediated photodegradation by ZnONPs (500 mg/L)	4 weeks	• Hepatic EROD, MDA, and 8-OHdG levels increased after a 4-week exposure to sewage<br>• Treatment of sewage effluent with ZnO nanoparticles reduced EROD activity and MDA and 8-OHdG levels in both exposure groups (TSE50 and TSE75) compared with untreated effluent<br>• Exposure to ZnO nanoparticles alone did not alter EROD, MDA, and 8-OHdG activity relative to the control	Alanazi et al. [53]
Red tilapia (*Oreochromis niloticus*)	PS-NPs (0.3, 5, and 70 − 90 μm)	100 μg /L	In water for 14 days	• PS-N/MP exposure elicited size- and time-dependent OS and neurotoxicity in tilapia<br>• All PS-N/MP treatments increased SOD activity compared to controls throughout exposure and SOD activity was affected by particle size and exposure time<br>• MDA levels were significantly influenced by particle size and exposure duration as 0.3 μm NPs and 5 μm MPs significantly increased MDA content (6 d)<br>• Longer exposures (14 d) led to lower MDA concentrations (0.3 μm NPs) and higher MDA (5 and 70–90 μm MPs)<br>• After 6 d, all N/MP sizes induced EROD activity<br>• 0.3 μm NP exposure consistently inhibited Benzyloxy-4-trifluoromethylcoumarin-O-debenzyloxylase (BFCOD) activity at	Ding et al. [54]

				both 6 and 14 d, with an 18.1% inhibition at 14 d<br>• 5 and 70–90 µm MPs exposures showed a transient increase followed by a decrease in BFCOD activity over time<br>• At 14 d, no significant differences in BFCOD activity were observed between control and MP treatments	
Red tilapia (*Oreochromis niloticusas*)	Aged (AM) and virgin (VM) PS MPs (5 µm) with and without a co-exposure to propranolol (PRP) and sulfamethoxazole (SMX)	Aged and virgin PS MPs (10 µg/L) and PRP and SMX (50 µg/L)	In water; 14-d uptake period, following by a 14-d depuration period. Fish were sampled on days 0, 7, 14, 21, and 28	• EROD in liver samples decreased in all treatments<br>• treatments of PRP, VM-PRP, and AM-PRP, decreased BFCOD<br>• activities of EROD and BFCOD were inhibited at different levels under most of the exposure conditions<br>• SOD activities in all treatments were significantly decreased at the end of exposure	Huang et al. [55]
Spotted snakehead (*Channa punctatus*)	PVC-MPs (0.315 µm) with and without Copper sulfate pentahydrate ($CuSO_4 \cdot 5H_2O$)	PVC-MPs (0.5 mg/L), Copper (0.85 mg/L), and a co-exposure	In water for 15, 30, 45, and 60-days	• ROS levels increased in all treatment groups<br>• The co-exposure group had the highest ROS levels<br>• Time-dependent increases in ROS also occurred<br>• Protein carbonyl (PC) content in the brain increased across exposure durations<br>• Both ROS production and PC content exhibited clear dose- and time-dependent increases, indicating progressive OS<br>• LPO levels demonstrated a clear dose- and time-dependent increase in OS in the fish brain<br>• Levels of 8-hydroxy-2′-deoxyguanosine (8-OHdG) in the fish brain increased over the exposure period<br>• Greatest elevation in DNA damage, with 8-OHdG levels rising in the combined exposure at day 60<br>• MAO activity in the fish brain increased in all treatment groups	Bakhasha et al. [56]
European hake (*Merluccius merluccius*)	115 MPs (36 % PE, 17 % PP, 10 % polymethyl methacrylate (PMMA), 5 % PET, 5 % PS, 3 % Polyacrylonitrile (PAN), 3 % PES and 2 % PA, 19% unidentified polymers)	Wild caught	Wild caught	• Induction in CAT-L (liver)	Lourenço et al. [57]
Pouting (*Trisopterus luscus*)	24 MPs (44 % PE, 25 % PP, 14 % PMMA, 4 % PS, 0.8 % PA, 0.8 % PES, 12% unidentified polymers)	Wild caught	Wild caught	• Induction in GR-L (liver)	Lourenço et al. [57]

Table S2. Cellular and molecular neurotoxicity changes witnessed in aquatic organisms following acute and chronic exposures to MP and NPs. These include changes in tissue morphology, DNA damage, and accumulation. *the same paper covers both invertebrates and vertebrates

Species	Plastic/size	Exposure Dose(s)	Exposure Method	Neurotoxic Effect(s)	Reference
**Invertebrates**					

Marine rotifers (*Brachionus plicatilis*) and Brine shrimp (*Artemia salina*)	PLA NPs (250 nm)	0.1, 1, and 100 mg/L	In exposure media for 24 h	• Accumulation was primarily observed in the digestive tract and showed a concentration-dependent increase in intensity; no toxic effects observed<br>• Brine shrimp experienced a decrease in ingestion ability	Mustapha et al. [1]*
Mediterranean Mussels (*Mytilus galloprovincialis*)	Leachate obtained from abandoned or lost fishing nets and cables of seven color groups (Contained PP, PE, and PA (various sizes)). Cu, Fe, Mn, Ni, Pb, and Zn, and other organic compounds were also detected in the leachate	1, 10, and 100 mg/L	In water for 0, 3, 7, 14, 21, and 28 days of exposure	• DNA damage increased after 7 and 14 days (10 mg/L)<br>• DNA damage increased between 3 and 21 days (100 mg/L)<br>• By 28 days, DNA damage levels in all treatments returned to control levels.	Vilke et al. [19]
Marine mussels (*Mytilus coruscus*)	PS-MPs (90 - 110 µm with and without TF and ENR	PS-MPs (400 µg/L)<br>TF (0.06 µg/L)<br>ENR (0.06 µg/L)	In seawater for 4 weeks	• Combined exposure to TF, ENR, or PS amplifies pollutant accumulation	Zhang et al. [23]
**Vertebrates**					
Tadpoles (*Xenopus tropicalis*)	TCS adsorbed on PE-MPs (38.8 µm) and aged PE-MPs (36.6 µm)	PE-MPs and aged PE-MPs (1 mg/L)<br>TCS (1 µg/L)	In water for 7 days	• Co-exposure to PE/aged PE-MPs + TCS increased TCS accumulation in tadpoles (aged PE+TCS > PE+TCS)	Lin et al. [25]
Glass eel (*Anguilla Anguilla)*	PS-MPs (10 µm)	Treatment 1 (0.04 mg/L)<br>Treatment 2 (0.2 mg/L)	In water for 21 days	• Particles (61) collected from eels were identified as rayon, PE, PAN/ polyacrylic (PAA), PES, polyethyleneimine (PEI), and PS	Gutierrez-Rodriguez et al. [58]
Zebrafish embryos (*Danio rerio*)	PS-NPs (20 nm)	3 nL of PNP stock solution was injected into the yolk sac of 4 hpf embryo (final concentration (~270 ppm)	120 h and changes recorded at 24, 48, 72, 96 and 120 hpf	• Cellular death was evaluated using acridine orange staining in whole live larvae at 120 hpf<br>• NP exposure resulted in an increase in overall cellular death<br>• Elevated apoptosis was consistent with other observed effects, including increased ROS levels and developmental malformations	Sökmen et al. [27]
Zebrafish embryos (*Danio rerio*)	Biomicroplastics (BioMPs) of PLA (2.34 ± 0.07 µm)	2.5 mg/L (BP I) and 5 mg/L (BP II)	In water for 30 days	• Exposure to BioMPs resulted in higher accumulation in the brain of the high-concentration group (BP II)	Chagas et al. [59]
Zebrafish embryos (*Danio rerio*)	MPs (1-5 µm) with and without copper	MPs (2 mg/L)<br>Cu (60 and 125 µg/L)	In water for 14 days	• MPs were ingested and accumulated in the gastrointestinal tract, eyes, and gills, supporting systemic exposure and potential toxicity<br>• Alterations were observed in retina and brain, with edema being the most frequent lesion across exposed groups<br>• Retinal layer disruption occurred only in Cu + MPs groups, suggesting enhanced visual system toxicity due to combined exposure<br>• MPs increased proliferating cell nuclear antigen (PCNA)-positive cells in the retina, possibly reflecting inflammation-induced proliferation or DNA repair<br>• Cu and Cu + MPs reduced PCNA-positive cells in retina and brain, indicating suppressed neurogenesis and cell proliferation	Santos et al. [60]
Zebrafish embryos (*Danio rerio*)	PS-NPs (-NH_2; 51 nm) and PS-NPs (-COOH; 50 nm)	PS-NH_2 and Polystyrene Anionic Carboxyl (PS-COOH) (30 and 50 mg/L)	In water for 24, 48, 72, 96, and 120 hpf	• Fluorescence was detected in the brain, gastrointestinal tract, and pericardium, indicating that both PS-NH_2 and PS-COOH crossed the Blood brain barrier (BBB) and entered the central nervous system (CNS)<br>• PS-NH_2 (50 mg/L) markedly increased brain fluorescence intensity	Teng et al. [61]
Zebrafish embryos (*Danio rerio*)	PS; 25 nm	10, 25, and 50 mg/L	In water for 4-100 hpf	• Highest NP accumulation (highest fluorescence intensity) occurred in the gall bladder and gut	Lin et al. [62]

				• Moderate NP fluorescence was observed in the head, eyes, and gills<br>• NP (fluorescent) signals were also detected in muscles, blood vessels, and lateral line neuromasts<br>• Fluorescence NP-labeled particles were present in circulating blood within the vessels<br>• Decreased Green fluorescent protein (GFP) intensity of spinal motor neurons (25 mg/L), indicating impaired motor neuron development<br>• Decrease eye area observed in the 25 mg/L treatment group<br>• Hair bundle (kinocilia) numbers in the lateral line were reduced at 25 and 50 mg/L	
Zebrafish embryos (*Danio rerio*)	PS-NP (30 nm)	PS-NPs (0.1, 0.5, and 3 mg/L)	In water for 120 hpf	• Zebrafish larvae (treated with 0.1 and 0.5 mg/L) had PS-NP accumulation in different organs at 24, 48, and 72 hpf<br>• Embryos accumulate PS-NP inside the chorion but mostly in the yolk<br>• Overall, accumulations in yolk > digestive tract (intestines) > pancreas > liver<br>• The eyes showed substantial PS-NP uptake as well as the brain and neuromasts of the lateral line also showed buildup<br>• Head and eye size were decreased in all treatments	Torres-Ruiz et al. [63]
Zebrafish embryos (*Danio rerio*)	Virgin and aged PS-MPs (1 µm)	0.1, 1, 10, and 100 µg/L	In media for 120 hpf	• PS-MPs were ingested by larvae and accumulated at 10 and 100 µg/L exposures subsequently impairing neuromotor behavior	Xiang et al. [64]
Zebrafish embryos (*Danio rerio*)	PMF from facial mask (surgical and N95) leachate;<br>• Surgical – length 112.7 ± 57.12 µm and width of 4.28 ± 1.16 µm (PP fibers plus aluminum (Al) and calcium (Ca))<br>• N95 – length 7.89 ± 11.69 µm and width of 2.15 ± 0.50 µm (PET fibers and Al, Ca, silicon (Si), chlorine (Cl), and potassium (K))	1000, 10,000, and 100,000 particle/L	In exposure media for 144 h	• No morphological changes in zebrafish embryos and larvae exposed to PMFs from both facial masks	Qualhato et al. [29]
Zebrafish embryos (*Danio rerio*)	PS-NPs (20 nm)	• 3 nL (microinjection)<br>1 ppm (waterborne)	Injected into the yolk sac of embryos 3 (nL) or in media (1ppm) for 5 days, then 5 days in media without NPs and examined after 6 months	• Histopathology: PS-NP exposure caused necrosis and degeneration in multiple brain regions, indicating structural damage<br>• Immunohistochemistry: Increased fluorescence signals in many regions suggesting upregulation of OS, apoptosis, and DNA damage markers<br>• Reduction in Neuropeptide Y (NPY) expression, a crucial brain chemical and neuronal growth regulator (NEGR)	Kankaynar et al. [30]
Zebrafish embryos (*Danio rerio*)	PLA NPs (250 nm)	0.1, 1, and 100 mg/L	In exposure media for 120 hpf	• In zebrafish embryos, PLA NPs were detected both in the digestive tract and in the eye region, with additional evidence of excretion through the cloaca. The fluorescent intensity at the highest concentration (100 mg/L) was significantly greater than at the lower concentrations (1 and 10 mg/L)	Mustapha et al. [1]*

				• No increased malformation prevalence was recorded in exposed embryos	
Zebrafish embryos (*Danio rerio*)	PS-MPs (5 µm) and PS-NPs (100 nm) with and without silver nanoparticles (Ag-NPs; 5 nm)	PS MPs/NPs (200 µg/L) and AgNPs (10 µg/L)	In media for ~120hpf	• AgNP-containing treatments reduced neuron numbers<br>• AgNP exposure caused pronounced axonal shortening and cerebellar and optic tectum hypoplasia<br>• AgNPs, MP, and NP exposures increased apoptosis, with NP showing the highest CNS apoptosis	Song et al. [32]
Zebrafish embryos (*Danio rerio*)	PS-NPs, anionic carboxyl PS-NPs-COOH, and cationic amino PSNPs-NH_2 (100 nm) combined with and without acrylamide (ACR)	Single exposures at 48h-LC_{50} -<br>AR (200 mg/L),<br>PS-NPs (150 mg/L),<br>PS NPs-COOH (15 mg/L),<br>PS NPs-NH_2 (0.25 mg/L),<br>combined exposures of ACR at LC_{50} + PS NPs, PS NPs-COOH or PS NPs-NH_2 at 1/3 LC_{50}, 2/3 LC_{50}, LC_{50}	In a 5 mL solution for 120 hpf	• Heart rate, a sensitive indicator of early neurodevelopment and cardiophysiological function in zebrafish, was increased in all exposure groups, indicating cardiotoxic effects of both PS-NPs and ACR<br>• GFP fluorescence was significantly suppressed in all exposure groups except PS NPs-NH_2, indicating widespread inhibition of CNS development<br>• Motor neuron development was markedly disrupted in all exposure groups, with transgenic larvae exhibiting axonal abnormalities compared with controls	Yang et al. [65]
Zebrafish embryos (*Danio rerio*)	PS-MPs (5 µm) and PS-NPs (60 nm)	0.05–50 mg/L	In water for 7dpf	• PS-NPs showed a concentration-dependent increase in fluorescence intensity and distribution in larvae, with significant accumulation at 5 and 50 mg/L	Li et al. [33]
Zebrafish embryos and larvae (*Danio rerio*)	PS-NPs (25 ± 0.6 nm) combined with and without ER antagonist ICI	PS-NPs (0.01, 0.1, 1, and 10 µg/mL) and ICI (10 µM)	In embryo medium for 2 - 120 hpf	• TH-positive neuron area (48 hpf): PSNP exposure (0.1, 1, and 10 µg/mL) caused a significant, dose-dependent reduction in the area of TH-positive neurons in zebrafish embryos, primarily in the anterior dorsal telencephalon and posterior ventral diencephalon (hypothalamic region)<br>• The highest reduction occurred at 10 µg/mL; 0.01 µg/mL had no significant effect.<br>• Co-incubation with 10 µM ICI reversed the reduction, restoring TH-positive neuron area to control levels<br>• Brain apoptosis (48 hpf): PS-NP exposure (0.1–10 µg/mL) caused a dose-dependent increase in brain apoptosis in zebrafish embryos, which was reversed by co-incubation with ICI, indicating ER-mediated neurotoxicity	Saputra et al. [66]
Zebrafish larvae (*Danio rerio*)	PS-NPs (80 nm) combined with 2,2 ‘,4,4 ‘-tetrabromodiphenyl ether (BDE-47)	PS-NPs (0.05, 0.1, 1, 5, 10 mg/L), BDE-47 (0.1 and 10 µg/L)	In water for 120 hpf	• No PS-NPs were detected inside the embryo at 48 hpf<br>• At 120 hpf, zebrafish larvae ingested PS-NPs orally and through the gills, which further entered the circulation and accumulated in the liver, heart and gastrointestinal tract	Wang et al. [34]
Zebrafish larvae (*Danio rerio*)	PS-NPs (100 nm) with and without PCB-153	PS-NPs (1.05 g/mL) PCB-153 (0.25, 0.5, 1, 2, 4, 8, and 16 µg/mL)	In E3 medium for 96h	• Fluorescent NPs were detected in caudal, gastrointestinal, and cranial regions of larvae exposed to NPs and NPs + PCB, confirming internalization<br>• Yolk sac autofluorescence was observed in all treatment groups	Varshney et al. [67]
Zebrafish larvae (*Danio rerio*)	PS-NPs (80 nm) combined with and without MEHP	PS-NPs (10, 25 and 50 µg/mL) and MEHP (10^{-6} and 10^{-5} M)	In embryo media for 24 hpf and 7 dpf	• Higher PS-NP concentration, longer exposure duration, and MEHP co-exposure synergistically promote nanoparticle accumulation in zebrafish larvae<br>• Brain immune effects: At 72 hpf, macrophages accumulated in the brain and heart	Liu et al. [36]

				• Macrophage numbers in the midbrain decreased with increasing PS-NP concentration.<br>• MEHP-enhanced immunotoxicity: Under 10^{-5} M MEHP, macrophage reduction was more pronounced, increasing PS-NP toxicity to brain immune cells<br>• Primordial hindbrain channel arteries (PHBC) alterations: vessels were shorter across exposure groups, indicating impaired cerebral vascular development<br>• NP25-induced mild nuclear elongation; co-exposure groups (MN1 and MN25) showed reduced neuron density and structural abnormalities, including neuronal swelling and nuclear elongation or contraction, indicating enhanced neurotoxicity under co-exposure<br>• Apoptosis: The number of apoptotic cells in the head and heart regions increased with rising PS-NP concentrations<br>• MEHP-enhanced neurotoxicity: MEHP co-exposure intensified PS-NP toxicity, increasing apoptotic cells in the brain	
Zebrafish larvae (*Danio rerio*)	PS-NPs (250 nm) with and without methylmercury (MeHg)	1000 μg/L NPs, 1 μg/L MeHg (MeHg1), 10 μg/L MeHg (MeHg10), or their respective combinations (Mix1 and Mix10)	In water for 30 days	• NPs increased MeHg accumulation in fish head rather than any other part of the body<br>• With 3765 ng/g ww, there was twice (exactly 2.08 times) as much MeHg accumulated in zebrafish larvae's heads than in whole larvae, showing significant accumulation in their head<br>• Cumulative, widespread bioaccumulation of NPs in the brain was detected in approximately 33% of the examined larvae<br>• DNA and oxidative damage present	Oger et al. [37]
Zebrafish (*Danio rerio*)	PS-NPs (44 nm), PS-NPs fluorescently labeled (42 nm), anionic carboxyl PS-NPs-COOH (51 nm), and cationic amino PS-NPs-NH_2 (51 nm)	10 μg/L	In water for 120 days (M and F spawned on day 113 to obtain F1 embryos for analysis)	• In male zebrafish, fluorescence intensity (FI) of PS-NH_2 and PS-COOH was elevated in the reticular formation (RF) compared to controls<br>• In female zebrafish, 10 μg/L PS-NH_2 caused a significant increase in FI in both the RF and ventral hypothalamus (Hv) regions<br>• PS-NH_2 exposure resulted in higher FI in RF and Hv regions for both sexes compared with PS, indicating enhanced brain accumulation of positively charged NPs<br>• BBB structure and integrity was disrupted in female zebrafish exposed to 10 μg/L PS or PS-COOH<br>• No significant BBB disruption was detected in male zebrafish exposed to differentially charged PS, suggesting a sex-dependent effect on barrier vulnerability<br>• Brain–somatic index was significantly reduced in PS-NH_2– and PS-COOH–exposed fish indicating inhibited brain development<br>• Histopathological analysis revealed clear brain damage in both sexes, including scattering of the periventricular gray matter layer, inflammatory cell infiltration, and colliculus mesencephali damage, with more extensive lesions observed following PS-NH_2 and PS-COOH exposure	Teng et al. [68]

				• Ultrastructural analyses demonstrated severe neuronal damage across all PS treatments, including nuclear membrane rupture, chromatin loss, mitochondrial swelling, cristae disruption, and compromised membrane integrity<br>• Transmission electron microscopy (TEM) revealed significant BBB disruption in all PS-exposed groups, characterized by irregular morphology and broken double membranes	
Zebrafish (*Danio rerio*)	PS; 0.6-1.0 µm	25 and 250 µg/L	In water for 40 days	PS-MPs accumulate in the zebrafish brain and induce neurotoxic effects including:<br>• accumulation of 1 µm PS-MPs in the zebrafish brain<br>• intensified nucleolar staining in the OT and Tel<br>• optic nerve layer also exhibited thinning<br>• perinuclear vacuoles in the Tel<br>• reduction in Nissl bodies in the Tel	Yang et al. [69]
Zebrafish (*Danio rerio*)	PS-MPs (2 and 25 µm) and PS-NPs (25 and 250 nm)	1 mg/L	In water for 14 days	• Across particle sizes and exposure durations, MP/NP accumulation followed the order: intestine > liver > gill > muscle > brain<br>• Accumulation of 25 nm NPs in the brain increased significantly from 1 to 14 days of exposure<br>• By 14 days of exposure, the brain consistently showed the lowest accumulation of small-sized NPs (25 nm and 250 nm) among all examined tissues	Su et al. [44]
Goldfish (*Carassius auratus*)	PS-MPs (30 µm) and PS-NPs (500 nm)	0.26 mg/L (low) and 0.69 mg/L (high)	In water for 28 days	• MPs induced structural damage in the olfactory bulb, including capillary congestion, cavitation, and expanded intercellular spaces<br>• Glial fibrillary acidic protein (GFAP) immunoreactivity was markedly increased, especially in NP groups, indicating astrocyte activation and neuroinflammation	Shi et al. [70]
Goldfish (*Carassius auratus*)	PP-MPs (6.37 µm) with and without OTC	PP-MPs (100 µg/L), OTC (200 µg/L)	In water for 21 days	• After 21 days of exposure, histopathological analysis revealed treatment-specific damage in fish brain tissues<br>• MPs-only exposure: intramedullary edema and occasional hairy cell astrocytomas<br>• OTC-only exposure: infectious inflammation and hairy cell astrocytomas<br>• Combined MPs + OTC exposure: more severe infectious inflammation<br>• Combined exposures caused pathological damage, with the combined treatment producing the most severe histopathological effects	Zhang et al. [49]
Grass carp (*Ctenopharyngodon idella*)	PS-MPs (5 µm)	1000 µg/L	In water for 7 days (28 h total, with 2-h sessions twice daily) in low, medium, and high velocity water	• After 7 days of exposure PS-MPs were detected in the brains of exposed fish<br>• PS-MP accumulation differed among treatments, with levels ranked as: MPs + high water velocity (HV) > MPs + medium velocity (MV) > MPs + low velocity (LV) > MPs alone<br>• Fish exposed to MPs + HV accumulated significantly higher PS-MP concentrations compared with all other treatments<br>• Fluorescence imaging confirmed the presence and localization of PS-MPs within the brain tissue of *Ctenopharyngodon Idella*	Rasta et al. [48]

				• Histopathological examination of juvenile grass carp brains revealed clear treatment-dependent differences<br>• Edema and tissue rupture in the periglomerular gray matter were evident in the LV, HV, MPs + LV, and MPs + MV treatments<br>• The MPs + HV treatment induced the most severe neuropathology, characterized by pronounced edema, tissue rupture, and cerebral hemorrhage	
Channel catfish (*Ictalurus punctatus*)	PS-NPs (80 nm) and BPA with and without Biochar	PS-NPs (0.5 mg/L)<br>BPA (0.2 mg/L)<br>Biochar (0.5 g/L)	In water for 7 days	• BPA concentrations in the gut, liver, and brain were slightly elevated in the BPA exposure group	Zheng et al. [50]
Javanese Medaka Fish (*Oruzias javicus*)	PS-MPs (5 μm)	PS-MPs<br>LOW (100 μg/L; 1.46×10^3), MED (500 μg/L; 7.3×10^3), and HIGH (1000 μg/L; 1.46×10^4)	In salt water (14.5 ± 0.5 ppt) for 21 days	• MP-LOW and MP-MED groups showed no obvious abnormalities<br>• MP-HIGH group had 26 ± 6% of slices showing cerebral edema	Usman et al. [52]
Red tilapia (*Oreochromis niloticus*)	PS-NPs (0.3, 5, and 70 − 90 μm)	100 μg /L	In water for 14 days	• M/NP accumulation in tilapia is tissue-, size-, and time-dependent, with the gut acting as the primary sink<br>• Intermediate-sized particles (5 μm) showed a unique accumulation pattern, particularly in the brain, suggesting enhanced translocation potential	Ding et al. [54]
Red tilapia (*Oreochromis niloticusas*)	Aged and virgin PS MPs (5 μm) with and without a co-exposure to PRP and SMX	Aged and virgin PS MPs (10 μg/L) and PRP and SMX (50 μg/L)	In water; 14-d uptake period, following by a 14-d depuration period. Fish were sampled on days 0, 7, 14, 21, and 28	• Accumulations of MPs followed the order of liver > brain > gills > gut in all the treatments<br>• both the virgin and aged MPs significantly increased the accumulation of PRP in the gut and brain<br>• MP aging increased the accumulation of PRP by 82.3% in the brain	Huang et al. [55]
Spotted snakehead (*Channa punctatus*)	PVC-MPs (0.315 μm) with and without Copper sulfate pentahydrate ($CuSO_4 \cdot 5H_2O$)	PVC-MPs (0.5 mg/L), Copper (0.85 mg/L), and a co-exposure	In water for 15, 30, 45, and 60-days	• Histological analysis revealed dose-dependent brain tissue damage in fish exposed to Cu and PVC-MPs including vacuolization, hypertrophy, degenerative glial cells, DNA damage, and apoptosis	Bakhasha et al. [56]

Table S3. Summary of neurochemical disruptions, including altered neurotransmitter release and degradative or hydrolytic enzyme responses, in aquatic organisms exposed acutely or chronically to MP and NP.

Species	Plastic/size	Exposure Dose(s)	Exposure Method	Neurotoxic Effect(s)	Reference
**Invertebrates**					
Marine rotifers (*Brachionus plicatilis*) and Brine shrimp (*Artemia salina*)	PLA NPs (250 nm)	0.1, 1, and 100 mg/L	In exposure media for 24 h	• No changes in acetylcholine esterase (AChE) activity for Marine rotifers or Brine Shrimp	Mustapha et al. [1]*
Brine shrimp nauplii and juveniles (*Artemia franciscana*)	Polymer MPs (1–5 μm)	0.4 and 1.6 mg/mL	In water for 2 and 5 days	• Juveniles, inhibition of Cholinesterase (ChE) (1.6 mg/mL) suggests neurotoxic effects, potentially contributing to higher mortality	Peixoto et al. [3]
Water flea (*Daphnia magna*)	Tire wear particles (TWP) MPs (0.7-70 μm) and leachate extraction from TWP for 30 and 60 min (E-30 and E-120)	TWP, 30-E, and 120-E (50, 100, 200, 400, 600, and 800 mg/L each)	In culture media for 24 and 48 h	• AChE activity was strongly induced by both particles and leachates, indicating neurotoxic stress	Liu et al. [4]

Mysid (*Neomysis awatschensis*)	PS-MP (1 µm) and As, Cd, Cu, Pb, and Zn	1. Metals (1/10th of LC_{50}),<br>2. Metals with fresh MPs (10 particles mL),<br>3. Metals premixed with MPs for 30 days, and<br>4. Metals premixed with MPs for 60 days	In water for 30 and 60 days	Juveniles:<br>• AChE activity was inhibited under metal + MPs exposure at 30 d with As and Zn compared with metals alone<br>• At 60 d, decreases in AChE activity were observed with Cu and Zn, indicating time-dependent neurotoxicity<br>Adults:<br>• AChE activity was reduced under metal + MPs (30 d) exposure with Pb<br>• At 60 d, AChE activity was decreased with Cu, Pb, and Zn, showing enhanced inhibition with prolonged exposure	Eom et al. [6]
Freshwater shrimp (*Caridina fossarum*)	PE-MPs (15–25 µm) with and without lead acetate	PE-MPs (500, and 1000 µg/L)<br>Lead (2.5, and 5 mg/L)	In water for 15 days	• Butyrylcholinesterase (BChE) activity was reduced by Pb, PE-MPs, and their mixture, with the strongest inhibition under co-exposure	Gholamhosseini et al. [7]
South American native shrimp (*Palaemon argentines*)	PLA-MPs (minor side: 116.8 ± 27.8 µm; major side: 175.8 ± 44.5 µm; mean area: 0.017 ± 0.007 mm^2) with and without MET	7.5 µg/L MET ($MET_{[7.5]}$)<br>5 mg/L PLA-MPs ($MPs_{[5]}$)<br>7.5 µg/L MET + 5 mg/L PLA-MPs ($MET_{[7.5]}$+$MPs_{[5]}$)	In water for 7 days	• AChE activity: Significantly inhibited in $MPs_{[5]}$ treatment, indicating neurotoxicity<br>• $MET_{[7.5]}$ alone showed a trend toward inhibition<br>• Co-exposure ($MET_{[7.5]}$+$MPs_{[5]}$) reversed AChE inhibition, restoring enzyme activity<br>• AChE inhibition could lead to acetylcholine (ACh) accumulation, overstimulation of receptors, impaired neurotransmission, and potential paralysis or death<br>• BChE activity: No significant changes in any treatment, indicating detoxification pathways via BChE were not affected	Bertrand et al. [8]
Red Swamp Crayfish (*Procambarus clarkii*)	MPs (size and type not stated) with and without PG	PG (10 mg/L),<br>MPs (100 mg/L), and Combination (10 mg/L PG + 100 mg/L MPs)	In water for 15 days	• Neurotoxicity biomarkers (Nitric oxide (NO) and AChE) were altered in following exposure to PG, MPs, and their combination<br>• NO levels decreased in the PG-exposed group<br>• NO showed a moderate decrease in the MPs-only group and a stronger decrease in the combined PG + MPs treatment<br>• AChE activity followed a pattern similar to NO<br>• AChE levels were reduced in the PG-exposed group<br>• AChE activity in the MPs-only and PG + MPs groups was similarly reduced	Hamed et al. [9]
Aquatic midge (*Chironomus riparius*)	PS; 1 µm	22,400, 112,000, and 224,000 plastics/kg sediments dry weight	In soil for 56 days (2 generations; 28 days for the 1st generation and other 28 days for the 2nd generation)	• No difference AChE across all treatments (2nd generation)	Sbarberi et al. [10]
Freshwater bivalves (*Corbicula fluminea*)	PS-MPs (6 µm) and PS-NPs (80 nm) with and without the antibiotic CIP	PS-MPs and PS-NPs (10 µg/g dw);<br>CIP (low; 0.5 µg/g and high; 5 µg/g, and 50 µg/g dry weight (dw))	In sediment for 10 days	• Significant reduction in AChE activity at 5 µg/g CIP and 5 µg/g CIP + PS-NP/MP treatments<br>• AChE activity negatively correlated with MDA content in co-treatments<br>• Decrease in AChE activity demonstrates CIP and CIP + MP/NP-PS induced neurotoxicity in *C. fluminea*	Guo et al. [11]

Marine clam (*Donax trunculus*)	PE-MPs (150–250 µm) with and without Gadolinium (Gd)	MPs (0.1 or 100 µg/L), Gd (500 µg/L), mixture of contaminants (0.1 MPs + 500 Gd µg/L; 100 MPs + 500 Gd µg/L)	In water for 14 days (examined every 7 days)	• AChE activity decreased after week 1 in the 500 Gd µg/L treatment and in both mixture (MPs + Gd) treatments<br>• After week 2, reduced AChE activity persisted in the 0.1 MPs µg/L + 500 Gd µg/L treatment<br>• A significant temporal effect was observed in the 0.1 MPs µg/L treatment, with AChE activity significantly higher in week 2 compared to week 1	Secco et al. [13]
Blue mussels (*Mytilus edulis*)	Pristine PE-MPs (27 – 45 µm) and Aged PE-MPs, with and without PAHs (phenanthrene and fluoranthene)	Pristine MPs (10 µg/L)<br>Phe (1 µg/L)<br>Fluo (0.5 µg/L)	In water for 8 days	• Mantle AChE activity was significantly higher in all MP-exposed groups	Moncrieffe et al. [14]
Mediterranean mussel (*Mytilus galloprovincialis*)	PET-microfibers (MF; 100 µm)	0.0005, 0.1, 1, 10, and 100 mg/L	For 32 days	• AChE was evaluated in the digestive glands and gills of mussels exposed to PET-MFs<br>• The increase in AChE activity may be linked to PET-MF–induced OS and tissue inflammation, which are associated with elevated ACh levels	Choi et al. [16]
Mediterranean mussel (*Mytilus galloprovincialis*)	PS-NPs (50 nm)	10 µg/L	In seawater for 28 days	• In gills of PS-NP–exposed mussels, AChE activity decreased after 3 days of exposure<br>• AChE activity in gills declined further over time, showing an approximately twofold reduction between days 3 and 28 of exposure	Gonçalves et al. [71]
Mediterranean mussel larvae (*Mytilus galloprovincialis*)	EMPs consisting of PE, PET, PP, PEVA, HDPE, and LDPE (sizes 1.22 to 30 µm) in control and high salinity salt water	EMPs (1, 10, 50, and 100 µg/L)<br>Control salinity (33 p.s.u)<br>High salinity (37 p.s.u)	In salt water (33 or 37 p.s.u) for 48 h	• AChE activity was affected by EMP exposure, salinity, and their combined effects in mussel D-larvae<br>• AChE activity at 33 p.s.u. was significantly inhibited at higher EMP concentrations (50 and 100 µg/L), indicating neurotoxic effects<br>• Elevated salinity alone (37 p.s.u.) increased AChE activities compared with 33 p.s.u.<br>• Combined exposure caused a concentration-dependent inhibition of AChE activity, with significant decreases at higher EMP levels	Boukadida et al. [15]
Mediterranean mussel (*Mytilus galloprovincialis*)	HDPE-MP (≤ 22 µm) and microalgae (MA; 3 and 8 µm) with and without chlorpyrifos (CPF)	MP (1.5 mg/L)<br>CPF (7.6 µg/L)<br>MP-CPF<br>MA-CPF	In sea water for 7 and 21 days	• Exposure to CPF, MA-CPF, and MP-CPF caused significant AChE inhibition in both gills and digestive gland at 7 and 21 days	Fernández et al. [17]
Mediterranean mussels (*Mytilus galloprovincialis*)	PS, PP, PET (80 - 240 µm) alone and co-exposed with Cadmium chloride ($CdCl_2$)	2 mg/L (~25,700 particles/L) plastics individually and with $CdCl_2$ (30 µg/L Cd^{2+})	In water for 7 days	• Decreased AChE in all groups	Tuncelli et al. [18]
Mediterranean mussels (*Mytilus galloprovincialis*)	Leachate obtained from abandoned or lost fishing nets and cables of seven color groups (Contained PP, PE, and PA (various sizes)). Cu, Fe, Mn, Ni, Pb, and Zn, and other organic compounds were also detected in the leachate	1, 10, and 100 mg/L	In water for 0, 3, 7, 14, 21, and 28 days of exposure	• Mussels exposed to 1, 10, or 100 mg/L leachate showed no significant changes compared with their respective controls<br>• A significant inhibition of AChE activity was detected at 10 mg/L after 7 days when compared with the highest concentration (100 mg/L)	Vilke et al. [19]
Marine mussels (*Mytilus coruscus*)	PLA MPs (5 µm, irregular fragments) with and without tris(1-chloro-2-propyl) phosphate (TCPP) an organophosphate	PLA MPs (10^2 and 10^6 particles/L)<br>TCPP (0.5 and 50 µg/L)	In seawater for 14 days	• Low TCPP exposure reduced AChE activity, and combined PLA+TCPP exposures caused further inhibition<br>• The strongest AChE inhibition occurred with low TCPP combined with high PLA	Zhong et al. [22]

	flame retardants				
Marine mussels (*Mytilus coruscus*)	PS-MPs (90 - 110 µm with and without TF and ENR	PS-MPs (400 µg/L) TF (0.06 µg/L) ENR (0.06 µg/L)	In seawater for 4 weeks	• Combined exposures (PS+TF, PS+ENR, ENR+TF) decreased AChE activity, indicating increased neurotoxicity	Zhang et al. [23]
Black sea urchin *Arbacia lixula*	PS-MPs (1 and 5 µm) alone and conjugated with BPA	PS-MPs (10 µg/mL) BPA (5 and 25 µM)	In exposure medium for 48 hpf	• Exposure to 1 and 5 µm PS MPs, 5 µM BPA, and 5 and 25 µM BPA adsorbed onto 1 and 5 µm PS MPs for and impaired the cholinergic nervous system in sea urchin embryos<br>• AChE activity was reduced in all exposed groups compared with controls with the strongest inhibition observed in embryos exposed to BPA alone and to BPA–PS MP conjugated treatments<br>• Neurotransmission pathways were consistently disrupted, with reductions in N-acetyl serotonin, choline, and tyrosine (Tyr) under all exposure conditions<br>• These neurotransmitter-related changes suggest impaired neuronal signaling and potential effects on neuromotor development	Eliso et al. [24]
Sea squirt (*Ciona robusta*)	PS NPs with and without bisphenol A (BPA)	BPA (0.01, 0.21, 0.69 mM) and PS NPs (0.0096–0.096 mM; 8.096 × 10^9–10^{10} particles, respectively)	For 15 min	• PS NPs did not affect the ChE activity alone or combined, while BPA caused a concentration-dependent inhibition of ChE activity in the viscera	Melki et al. [72]
**Vertebrates**					
Tadpoles (*Xenopus tropicalis*)	TCS adsorbed on PE-MPs (38.8 µm) and aged PE-MPs (36.6 µm)	PE-MPs and aged PE-MPs (1 mg/L) TCS (1 µg/L)	In water for 7 days	• Exposure to PE/aged PE-MPs and TCS increased AChE activities, with the highest effects in the aged PE+TCS group	Lin et al. [25]
Tadpoles (*Rana nigromaculata*)	PS-MPs 0.1 µm (MP1), 1 µm (MP2), and 10 µm (MP3)) with and without levofloxacin (LVFX)	MPs (0.10 mg/L) LVFX (1.00 µg/L)	In water for 45 days	• LVFX–MP co-exposure alters gut neurotransmitter metabolism (alterations in His, Glu, Leu, Met, Phe, and Val, Orn) via microbiota-mediated pathways, with MP particle size playing a key role in determining the magnitude and direction of neurotoxic effects	Zhang et al. [26]
Zebrafish embryos (*Danio rerio*)	BioMPs of PLA (2.34 ± 0.07 µm)	2.5 mg/L (BP I) and 5 mg/L (BP II)	In water for 30 days	• AChE activity was significantly increased in PLA BioMP–exposed groups compared with controls, with no clear concentration–response relationship	Chagas et al. [59]
Zebrafish embryos (*Danio rerio*)	PS-NPs (100 nm) with and without Avobenzone (AVO)	NPs (10 µg/L) AVO (10 µg/L)	In media for 144 h followed by a 72-h recovery in dH_2O	• AChE activity increased significantly at 144 h in all exposure groups but returned to control levels after recovery	Liu et al. [28]
Zebrafish embryos (*Danio rerio*)	PS-NPs (-NH_2; 51 nm) and PS-NPs (-COOH; 50 nm)	PS-NH_2 and PS-COOH (30 and 50 mg/L)	In water for 24, 48, 72, 96, and 120 hpf	• PS-COOH increased polyamine-related metabolites (tyramine, spermine, spermidine), potentially promoting locomotor activity<br>• PS-NH2 elevated kynurenine, VMA, lysine, putrescine, and spermidine, implicating disruption of tryptophan metabolism, monoaminergic signaling, and stress-related pathways<br>• Metabolic changes under PS-NH2 exposure are consistent with reduced serotonin (5-HT) and norepinephrine (NE) signaling and impaired neurobehavior	Teng et al. [61]
Zebrafish embryos (*Danio rerio*)	PS; 25 nm	10, 25, and 50 mg/L	In water for 4-100 hpf	• Exposure resulted in suppression of AChE	Lin et al. [62]
Zebrafish embryos (*Danio rerio*)	PS-NP (30 nm)	PS-NPs (0.1, 0.5, and 3 mg/L)	In water for 120 hpf	• AChE was decreased concentration dependent manner with 0.1, 0.5, and 3 mg/L exposed larvae having 18, 19, and 20% less activity (120 hpf)	Torres-Ruiz et al. [63]

				• Dopaminergic pathway genes were also disrupted:<br>• Significant concentration-dependent upregulation of *th1*<br>• Dopamine (DA) receptor genes *drd2a* and *drd2b* displayed biphasic expression patterns, with significant upregulation at all exposure concentrations and maximal induction at 0.5 mg/L	
Zebrafish embryos (*Danio rerio*)	Virgin and aged PS-MPs (1 μm)	0.1, 1, 10, and 100 μg/L	In media for 120 hpf	• Exposure to aged PS-MPs caused concentration-dependent increases in DA, 5-HT, Gamma-Aminobutyric Acid (GABA), and ACh levels<br>• Altered neurotransmitter profiles were associated with reduced locomotor performance, indicating neurotoxicity	Xiang et al. [64]
Zebrafish embryos (*Danio rerio*)	Virgin and photoaged PS (1 μm) with and without adsorbed clothianidin (CLO)	PS-MPs (100 μg/L)<br>CLO (3 μg/L)	In water for 5 days	• Results indicate that photoaged MPs amplify CLO-induced neurochemical and transcriptional disruptions, contributing to behavioral abnormalities in zebrafish larvae<br>• Neurotransmitter levels (5-HT, ACh, DA, GABA) were significantly altered in zebrafish larvae following CLO exposure alone<br>• Co-exposure to PS or photoaged PS with CLO<br>• Increased ACh, DA, and GABA levels<br>• Decreased 5-HT levels<br>• The P-PS + CLO group showed significantly different 5-HT and GABA levels compared to the PS + CLO group, indicating enhanced neurotoxicity after photoaging<br>• Neurotransmitter-related gene expression was significantly affected by PS + CLO co-exposure<br>• Upregulation of *gabra1*<br>• Downregulation of *5ht1b*, *5ht2c*, *drd3*, *drd1*, and *ache*<br>• Compared with PS + CLO, P-PS + CLO co-exposure further increased mRNA levels of *gad1b*, *gabra1*, and *drd1*	Ding et al. [73]
Zebrafish embryos (*Danio rerio*)	PLA NPs (250 nm)	0.1, 1, and 100 mg/L	In exposure media for 120 hpf	• No changes in AChE activity for zebrafish	Mustapha et al. [1]*
Zebrafish embryos (*Danio rerio*)	PS-MPs (5 μm) and PS-NPs (60 nm)	0.05–50 mg/L	In water for 7dpf	• PS-NPs broadly reduced DA, ACh, GABA, and 5-HT levels, with significant decreases in DA and GABA at all concentrations<br>• PS-MPs significantly reduced DA, ACh, and 5-HT at 0.5 and 5 mg/L, and GABA at 0.5 mg/L	Li et al. [33]
Zebrafish embryos and larvae (*Danio rerio*)	PS-NPs (25 ± 0.6 nm) combined with and without ER antagonist ICI	PS-NPs (0.01, 0.1, 1, and 10 μg/mL) and ICI (10 μM)	In embryo medium for 2 - 120 hpf	• AChE activity (120 hpf): Exposure to PSNPs (0.1–10 μg/mL) caused a significant, dose-dependent reduction in AChE activity, with the greatest decrease at 10 μg/mL, while the lowest concentration (0.01 μg/mL) had no significant effect; co-incubation with ICI restored activity to control levels	Saputra et al. [66]
Zebrafish larvae (*Danio rerio*)	PLABioMPs (2.34 ± 0.07 μm)	3 and 9 mg/L	In water for 5 days	• AChE activity was reduced in PLA BioMPs–exposed larvae, suggesting cholinergic disruption	De Oliveira et al. [74]
Zebrafish larvae (*Danio rerio*)	Polymer MP (1–5 μm) with and without copper sulfate pentahydrate	MPs (2 mg/L)<br>Cu (60 and 125 μg/L)	In water for 14 days	• AChE activity was significantly decreased in all exposed groups after 14 days compared to the control<br>• Cu + MPs co-exposure caused a greater inhibition of AChE than the corresponding Cu-only groups	Santos et al. [75]

				• Overall pattern of AChE inhibition: Cu + MPs > MPs > Cu > control	
Zebrafish larvae (*Danio rerio*)	Aged PS-MPs (1 µm) and Thiamethoxam (TMX)	Aged PS-MPs (1 µg/L) TMX (1.5 µg/L)	In water for 120 hpf	• Aged PS and TMX increased ACh and GABA levels and decreased 5-HT content<br>• These neurotransmitter imbalances were amplified in the combined exposure, consistent with enhanced neurotoxicity	Sun et al. [35]
Zebrafish larvae (*Danio rerio*)	PS-NPs (80 nm) combined with and without MEHP	PS-NPs (10, 25 and 50 µg/mL) and MEHP (10^{-6} and 10^{-5} M)	In embryo media for 24 hpf and 7 dpf	• AChE inhibition: Co-exposure to PS-NPs and MEHP significantly reduced AChE activity by suppressing *ache* gene expression rather than through direct enzymatic inhibition, indicating impaired neural transmission<br>• Molecular pathway disruption: co-exposure (MN25) disrupted the glycerophospholipid–choline/ACh pathway linked to AChE, with significant downregulation of *gpx4a, dok7, hspg2, and gpcpd1*, confirming transcriptomic alterations associated with impaired cholinergic metabolism	Liu et al. [36]
Zebrafish larvae and adults (*Danio rerio*)	Schwarzbach River water samples containing PE (50%), PP (25%), PS (15%), and PVC (10%) and sorbed contaminants (up to 94 different compounds)	100 mg/L (except larval behavior tests 5 mg/L)	In river water for 24, 48, 72, and 96 hpf (larval fish) and 21 days for adult fish	Schwarzbach water exposure:<br>• AChE - 6–11% reduction overall, with significant inhibition at the final sampling	Hanslik et al. [40]
Zebrafish juveniles (*Danio rerio*)	PET-MPs (0.15 mm) with and without abamectin (ABM)	PET-MPs (5 mg/L and 10 mg/L)<br>ABM (0.006 mg/L)	In water for 96 h	• AChE activity: Inhibited in both ABM-alone and MP-only, whereas combined ABM + MP exposures did not result in any changes	Kazemi et al. [38]
Zebrafish juveniles (*Danio rerio*)	PS-MPs and PS-NPs with and without PFOA	PS-MPs (0.1 µg/mL)<br>PS-NPs (0.1 µg/mL)<br>PFOA (200 ng/L)	In water for 7 and 28 days	• Co-exposure of PFOA with MPs or NPs increased intestinal AChE activity relative to PFOA alone, suggesting partial mitigation of neurotoxicity via reduced PFOA bioavailability	Wu et al. [39]
Zebrafish (*Danio rerio*)	PS-NPs (70 nm)	PS-NPs (0.5 and 1.5 ppm)	In water for 7 days	• AChE activity was inhibited in the 1.5 ppm PS-NPs group<br>• DA, melatonin, GABA, 5-HT, vasopressin, kisspeptin, and oxytocin levels were reduced in the 1.5 ppm PS-NPs group	Sarasamma et al. [41]
Zebrafish (*Danio rerio*)	PE-MPs (45–53 µm)	0.6 mg/L	In water for 1, 5, and 10 days	• The activity of AChE decreased only after MPs exposure for 5 days	Xue et al. [76]
Zebrafish (*Danio rerio*)	PS-NPs (100 nm) with and without and arsenic (As)	PS-NPs (1 mg/L)<br>As (1 mg/L)	In water for 30 days	• Combined exposure to NP + As caused a decrease in 5-HT levels in the brain, serum, and intestines (brain–gut axis), exceeding the effects observed with single exposures	Zhang et al. [43]
Zebrafish (*Danio rerio*)	PS; 0.6-1.0 µm	25 and 250 µg/L	In water for 40 days	Decreased neurotransmitters<br>• DA, ACh, Tyr, Trp, Levodopa (DOPA), GABA, 5-HT, NE	Yang et al. [69]
Zebrafish (*Danio rerio*)	PS-MPs (2 and 25 µm) and PS-NPs (25 and 250 nm)	1 mg/L	In water for 14 days	• AChE activity in the brain increased after 1 day of MP and NP exposure, indicating an acute neurochemical response<br>• Small-sized NPs (25 nm) induced higher AChE activity than larger particles (250 nm), suggesting greater neurotoxic potential of smaller particles	Su et al. [44]
Zebrafish (*Danio rerio*)	PE MPs 13.5 µm) and NPs (70 nm) with and without PFOA	MPs and NPs (0.1 µg/mL) and PFOA (200 ng/L)<br>Exposure groups: PFOA, PE-MPs + PFOA, PE-NPs + PFOA, and PE-MPs + PE-NPs + PFOA	In water for 21 days (sampling every 7 days)	• AChE activity patterns in the liver and gills showed higher activity in PFOA-only exposure compared to combined exposures, suggesting antagonistic interactions that reduced neurotoxicity under co-exposure conditions	Wang et al. [45]

Goldfish (*Carassius auratus*)	PS-MPs (30 µm) and PS-NPs (500 nm)	0.26 mg/L (low) and 0.69 mg/L (high)	In water for 28 days	• ACh and DA levels were decreased following MP exposure, particularly at higher doses and smaller particle sizes<br>• GABA levels were increased in all MP-exposed groups, suggesting altered inhibitory neurotransmission	Shi et al. [70]
Goldfish (*Carassius auratus*)	PP-MPs (6.37 µm) with and without OTC	PP-MPs (100 µg/L), OTC (200 µg/L)	In water for 21 days	• Individual and combined exposures reduced AChE and BChE activities in the fish brain with the combined exposure having the displaying the strongest inhibition<br>• MPs-only exposure increased GABA levels<br>• OTC-only exposure increased ACh levels while suppressing 5-HT<br>• Combined MPs + OTC exposure markedly elevated ACh and GABA levels and significantly reduced 5-HT	Zhang et al. [49]
Crucian carp (*Carassius carassius*)	PA of various shapes; 0.43-255 µm	0, 4, 8, 16, 32 and 64 mg/L	In water, for 1 and 2 weeks	• AChE was decreased at 32 and 64 mg/L after 1 and 2 weeks of exposure	Choi et al. [77]
Grass carp (*Ctenopharyngodon idella*)	PS-MPs (5 µm)	1000 µg/L	In water for 7 days (28 h total, with 2-h sessions twice daily) in low, medium, and high velocity water	• AChE activity was significantly reduced in the MPs + HV group	Rasta et al. [48]
Streaked prochilod (*Prochilodus lineatus*)	PE-MPs (10–90 µm) with and without copper	PE-MPs (20 µg L) Copper (II) dihydrate (10 µg L)	In water for 24 and 96 h	• Brain AChE activity was decreased in all exposed groups after 96 h	Roda et al. [78]
Channel catfish (*Ictalurus punctatus*)	PS-NPs (80 nm) and BPA with and without Biochar	PS-NPs (0.5 mg/L) BPA (0.2 mg/L) Biochar (0.5 g/L)	In water for 7 days	Nervous system-related enzyme activities (brain):<br>• ACh concentrations: Reduced in PS group<br>• Choline acetyltransferase (ChAT) activity: Reduced in PS group and BPA group<br>• AChE activity: Increased in PS group	Zheng et al. [50]
African catfish (*Clarias gariepinus*)	PVC-MPs (95.41 ± 4.23 µm)	0.50%, 1.50% and 3.0% in the diet	In the diet for 45 days and depuration for 30 days	• AChE changes were observed across exposure durations, with reduced activity at day 45 and increased activity following depuration	Iheanacho and Odo [51]
Javanese Medaka Fish (*Oruzias javicus*)	PS-MPs (5 µm)	PS-MPs LOW (100 µg/L; 1.46 × 10^3), MED (500 µg/L; 7.3 × 10^3), and HIGH (1000 µg/L; 1.46 × 10^4)	In salt water (14.5 ± 0.5 ppt) for 21 days	• AChE activity significantly decreased in MP-MED and MP-HIGH versus control<br>• ACh levels progressively increased in all PS-MP–exposed groups compared to control	Usman et al. [52]
Red tilapia (*Oreochromis niloticus*)	PS-NPs (0.3, 5, and 70 − 90 µm)	100 µg /L	In water for 14 days	• Brain AChE rates were reduced in all N/MP treatments, with inhibition rates of 14.5% (0.3 µm NPs), 21.0% (5 µm MPs), and 9.1% (70–90 µm MPs)<br>• 5 µm MP exposure caused a significant inhibition of AChE compared to control at 14 d	Ding et al. [54]
Red tilapia (*Oreochromis niloticusas*)	Aged and virgin PS MPs (5 µm) with and without a co-exposure to PRP and SMX	Aged and virgin PS MPs (10 µg/L) and PRP and SMX (50 µg/L)	In water; 14-d uptake period, following by a 14-d depuration period. Fish were sampled on days 0, 7, 14, 21, and 28	• AChE in all treatment groups showed significantly lower activities after 14-d exposure ($p < 0.05$), with the inhibition rates ranging from 36.3% to 67.0%	Huang et al. [55]
Nile tilapia (*Oreochromis niloticus*)	Light-induced zinc oxide NPs (ZnONPs; 220 nm)	NPs (10, 20, and 30 mM)<br>• ZnONPs (5 mg/L)<br>• 50% sewage effluent<br>• 75% sewage effluent<br>• 50% sewage treated effluent UV mediated photodegradation by ZnONPs (500 mg/L)	21 days	• Four-week exposure to sewage effluent decreased brain AChE activity at both SE50 and SE75<br>• Treatment of sewage effluent with ZnO nanoparticles restored AChE activity in both exposure groups<br>• Exposure to ZnO nanoparticles alone did not significantly affect	Alanazi et al. [53]

		• 75% sewage treated effluent UV mediated photodegradation by ZnONPs (500 mg/L)		brain AChE activity compared with controls	
Spotted snakehead (*Channa punctatus*)	PVC-MPs (0.315 µm) with and without Copper sulfate pentahydrate ($CuSO_4 \cdot 5H_2O$)	PVC-MPs (0.5 mg/L), Copper (0.85 mg/L), and a co-exposure	In water for 15, 30, 45, and 60-days	• ChAT activity in the fish brain decreased over the exposure period, indicating reduced ACh synthesis<br>• The strongest reduction occurred following the co-exposure for 60 days<br>• A clear dose- and time-dependent impairment of ACh synthesis in neurons was observed<br>• Reductions in AChE activity were observed in all treated groups compared to controls, showing a progressive decline over time<br>• Significant decreases in DA and 5-HT levels occurred across all exposure groups	Bakhasha et al. [56]
Painted comber (*Serranus scriba*)	PEVA and HDPE and other smaller size classes including PS, PA, and LDPE (3–1.2 µm and 1.2–0.45 µm) and other chemicals	Wild caught	Wild caught	• AChE activity decreased in most polluted sites, suggesting cholinergic neurotoxicity	Zitouni et al. [46]
Common carp (*Cyprinus carpio*)<br>Sea mullet *(Mugil cephalus)*<br>European flounder (*Platichthys flesus*)	Wild caught - MPs (150–1499 µm) dominated and polymer profiles differed among species and seasons	Wild caught in March and September	Wild caught in March and September	• MP-H carps showed signs of depressed brain AChE<br>• MP-H mullets exhibited strong neurotoxicity signals (AChE inhibition)<br>• MP-H flounders showed altered muscle ChE activity, with season-dependent patterns	Martins et al. [47]
European hake (*Merluccius merluccius*)	115 MPs (36 % PE, 17 % PP, 10 % PMMA, 5 % polyethylene terephthalate (PET), 5 % PS, 3 % PAN, 3 % polyester (PES) and 2 % PA, 19% unidentified polymers)	Wild caught	Wild caught	• AChE (brain) and ChE (dorsal muscle) unchanged	Lourenço et al. [57]
Pouting (*Trisopterus luscus*)	24 MPs (44 % PE, 25 % PP, 14 % PMMA, 4 % PS, 0.8 % PA, 0.8 % PES, 12% unidentified polymers)	Wild caught	Wild caught	• AChE (brain) and ChE (dorsal muscle) unchanged	Lourenço et al. [57]

Table S4. Genetic changes witnessed in aquatic organisms following acute and chronic exposures to MP and NPs. These gene changes include neuronal growth, apoptosis, stress, and genes regulating neurotransmitters.

Species	Plastic/size	Exposure Dose(s)	Exposure Method	Physiological Effect(s)	Reference
**Invertebrates**					
Water flea (*Simocephalus vetulus*)	PS-NPs (500 nm)	1, 5 and 10 mg/L	In water for 14 days	• Stress and protective response genes HSP70 and MPV17 showed increased expression<br>• Genes for SOD and GST were upregulated after exposure<br>• The upregulation of these genes indicates activation of cellular defenses against LPO and mitochondrial damage	Zhu et al. [5]
Freshwater bivalves (*Corbicula fluminea*)	Commercial PS-NPs (200 nm) and NPs generated	Microalgae were contaminated for 48 h with	Bivalves were then fed with the contaminated	OS genes:<br>• *sod1*, *cat*, and *gpx7* were downregulated under PS 100 (7 days),	Latchere et al. [12]

	from macro-sized plastics collected in the field (ENV-NPs; 235 ± 70 nm)	0.008, 10 and 100 µg/L PS-NPs and ENV-NPs	algae every 48 h for 21 days	ENV NP 0.008 (7 days), and ENV NP 100 (7 days), respectively<br>Neurotoxicity gene:<br>• *AchE* was downregulated only in organisms exposed to ENV NP 0.008.<br>Apoptosis genes:<br>• Exposure to PS NPs repressed *bax* (PS 10 and 100) and *gadd45* (PS 0.008)	
Mediterranean mussel larvae (*Mytilus galloprovincialis*)	EMPs consisting of PE, PET, PP, PEVA, HDPE, and LDPE (sizes 1.22 to 30 µm) in control and high salinity salt water	EMPs (1, 10, 50, and 100 µg/L)<br>Control salinity (33 p.s.u)<br>High salinity (37 p.s.u)	In salt water (33 or 37 p.s.u) for 48 h	• Genes associated with antioxidant defense (*cat, gst*), apoptosis (*caspase-3, bax*), DNA repair (*p53, DNA-ligase, bcl-2*), shell formation (*hrg, tubulin*), and stress response (*hsp27, hsp70, calreticulin*) were differentially regulated by EMP exposure, salinity, and their combination<br>• At control salinity (33 p.s.u), increasing EMP concentrations significantly upregulated *cat, hrg, tubulin, p53*, *DNA-ligase*, and *bcl-2* expression in mussel D-larvae<br>• Exposure to elevated salinity alone (37 p.s.u) induced strong upregulation of multiple genes, including *cat, gst, tubulin, p53, DNA-ligase, bax, hsp27*, and *calreticulin*, indicating enhanced OS, DNA damage responses, apoptosis, and cellular stress<br>• Combined high salinity and EMP exposure further increased *gst* expression at low EMP concentrations (1 and 10 µg/L), suggesting an amplified antioxidant response under co-stress<br>• Apoptosis-related genes *caspase-3* and *bax* were consistently upregulated across most EMP and salinity treatments<br>• At the highest EMP concentrations (50 and 100 µg/L), expression of *DNA-ligase* and *hsp70* was reduced, indicating impaired DNA repair capacity and stress response<br>• A marked repression of the shell formation gene *hrg* was observed at the highest EMP concentration (100 µg/L), suggesting potential disruption of shell development under severe exposure conditions	Boukadida et al. [15]
Marine mussels (*Mytilus coruscus*)	PS-MPs (2.0 µm)	Four concentration exposures (0, 10, 10^4 and 10^6 particles/L)	In water for 14 days followed by a 7-day recovery period	• mRNA expression of heat shock protein Hsp70 was significantly upregulated at 10^6 particles/L and increased with longer exposure, indicating a protective cellular response<br>• Heat shock proteins are essential for protecting cells and repairing damaged proteins under stress conditions	Huang et al. [20]
Marine mussels (*Mytilus coruscus*)	PS-MPs (90 - 110 µm with and without TF and ENR	PS-MPs (400 µg/L)<br>TF (0.06 µg/L)<br>ENR (0.06 µg/L)	In seawater for 4 weeks	• Co-exposure significantly upregulated CYP1A1 and GST genes more than single exposures, activating detoxification pathways	Zhang et al. [23]
**Vertebrates**					
Tadpoles (*Xenopus tropicalis*)	TCS adsorbed on PE-MPs (38.8 µm) and aged PE-MPs (36.6 µm)	PE-MPs and aged PE-MPs (1 mg/L)<br>TCS (1 µg/L)	In water for 7 days	• Combined aged PE+TCS exposure upregulated developmental (*PPARα/γ, nkx2.5*), antioxidant (*sod, cat*), neuroprotective (*5-HT*), and inflammatory genes (*IL-1β, IL-6*), linking bioaccumulation to OS, neurotoxicity, inflammation, and developmental effects	Lin et al. [25]
Glass eel (*Anguilla Anguilla*)	PS-MPs (10 µm)	Treatment 1 (0.04 mg/L)<br>Treatment 2 (0.2 mg/L)	In water for 21 days	• Differences in the expression of genes related to OS (*sod1*, *sod2*, *cat*, and *gpx1*), neurotoxicity (*ache*), and metal	Gutierrez-Rodriguez et al. [58]

				detoxification (*mt2*) were detected among estuaries	
Zebrafish embryos (*Danio rerio*)	PS-NPs (100 nm) with and without Avobenzone (AVO)	NPs (10 µg/L)<br>AVO (10 µg/L)	In media for 144 h followed by a 72-h recovery in dH_2O	• AVO primarily altered genes involved in nervous system development, with early upregulation of *α1-tubulin, elavl3, gap43, gfap, mbp*, and *syn2a*, and downregulation of *lfng*; these effects were transient and largely recovered<br>• Both AVO and NPs disrupted stem cell differentiation–related genes (*foxg1, her5, her6, shha, sox2*), with most changes reversible after recovery<br>• NPs mainly affected retinal development genes (↓*lhx9*, *six6*; ↑*pax2*, *pax6*, *six3*), with some effects persisting after recovery; AVO partially attenuated NP effects on *lhx9*	Liu et al. [28]
Zebrafish embryos (*Danio rerio*)	MPs (1-5 µm) with and without copper	MPs (2 mg/L)<br>Cu (60 and 125 µg/L)	In water for 14 days	• MPs and Cu suppressed key neural stem/proliferation markers (*pcna, sox2*), particularly at early developmental stages, indicating impaired neural progenitor cell proliferation<br>• *NeuroD* and *olig2* were downregulated by MPs and Cu, suggesting disrupted neuronal differentiation and motor neuron specification<br>• Islet genes (*islet1*, *islet2a*, *islet2b*) were consistently downregulated during early development, pointing to impaired motor neuron determination and potential locomotor dysfunction	Santos et al. [60]
Zebrafish embryos (*Danio rerio*)	PS-NPs (-NH_2; 51 nm) and PS-NPs (-COOH; 50 nm)	PS-NH_2 and PS-COOH (30 and 50 mg/L)	In water for 24, 48, 72, 96, and 120 hpf	• PS-NH2 (50 mg/L) increased cellular apoptosis<br>• Key apoptosis-related genes (*cas3, cas9, bcl2, bax, mdm*) were broadly downregulated, particularly under PS-NH2 exposure, indicating disruption of mitochondrial apoptotic signaling<br>• Overall, apoptotic signaling was more severely affected by PS-NH2 than PS-COOH	Teng et al. [61]
Zebrafish embryos (*Danio rerio*)	PS-MPs (5 µm) and PS-NPs (60 nm)	0.05–50 mg/L	In water for 7dpf	• PS-NPs upregulated antioxidant genes (*sod1* and *cat*), indicating an adaptive OS response<br>• PS-NPs upregulated *pcna* (0.05 mg/L) and *bdnf* (50 mg/L), while downregulating *gfap* (5 mg/L)<br>• PS-NPs caused marked downregulation of *mbpa* and *ache* across all concentrations<br>• PS-MPs repressed *pcna* and *bdnf*, but upregulated *gap43* at low concentrations, suggesting a compensatory neural response<br>• Expression of *sox2* and *tuba1a* was unaffected by either particle type	Li et al. [33]
Zebrafish embryos (*Danio rerio*)	PS; 25 nm	10, 25, and 50 mg/L	In water for 4-100 hpf	Gene expression in zebrafish embryos:<br>• *ache, pax6a, otx2*: mRNA levels decreased at 25 and 50 mg/L<br>• *syn2a, mbp, pax6b, rx1*: decreases only observed at 50 mg/L	Lin et al. [62]
Zebrafish embryos (*Danio rerio*)	PS-NPs (30 nm)	0.1, 0.5 and 3 ppm	In media for 120 hpf	• *ache* mRNA expression was significantly downregulated at 0.5 and 3 ppm NP, suggesting neurotoxic effects<br>• caspase-1 and caspase-8 were upregulated at 3 ppm, indicating activation of inflammatory and apoptotic pathways<br>• Anti-apoptotic genes *hsp70* and *bcl2a* were downregulated, supporting apoptosis progression<br>• *il1β* expression increased in a concentration-dependent manner	Martin-Foglar et al. [79]

				• SOD1 and SOD2 were upregulated at 3 ppm, indicating OS induction at the highest NP concentration<br>• *gadd45α* and *rad51* showed downregulation at higher concentrations, suggesting impaired DNA repair capacity<br>• *hsp70* expression was dose-dependently downregulated, with a significant decrease at 3 ppm<br>• *hsp90* and *hsp27* expression were unchanged, suggesting an altered stress and anti-apoptotic response	
Zebrafish embryos (*Danio rerio*)	PS-NP (30 nm)	PS-NPs (0.1, 0.5, and 3 mg/L)	In water for 120 hpf	• Hypothalamic–pituitary–thyroid (HPT) axis genes were significantly affected:<br>• Dose-dependent downregulation of *trh*, *trhr*, and *tshb*.<br>• Dose-dependent upregulation of *tshr*, *trα*, and *ugt1ab*.<br>• Hypothalamic–pituitary–interrenal (HPA) axis genes exhibited marked transcriptional responses:<br>• Upregulation of *crha*, *crhpb*, and *acthb*<br>• *crhb* showed a biphasic response, with upregulation at 0.1 and 0.5 mg/L followed by reduced expression at 3 mg/L	Torres-Ruiz et al. [63]
Zebrafish embryos (*Danio rerio*)	Virgin and aged PS-MPs (1 μm)	0.1, 1, 10, and 100 μg/L	In media for 120 hpf	• Aged PS-MPs altered neurotransmission-related gene expression<br>• DA- and 5-HT–related genes were generally downregulated, while GABA- and ACh–related genes were upregulated<br>• High concentrations of aged PS-MPs produced stronger transcriptional effects	Xiang et al. [64]
Zebrafish embryos (*Danio rerio*)	PS-NPs (20 nm)	• 3 nL (microinjection) 1 ppm (waterborne)	Injected into the yolk sac of embryos 3 (nL) or in media (1ppm) for 5 days, then 5 days in media without NPs and examined after 6 months	• Downregulation SOD, GPx, and OX, indicates a compromised ability to neutralize ROS<br>• Increased MAO in injected embryos and CAT in waterborne treatment<br>• Increased 8-OHdG, a biomarker for OS and DNA damage, expression in key brain regions<br>• Reduction in NPY expression, a crucial brain chemical and NEGR	Kankaynar et al. [30]
Zebrafish embryos (*Danio rerio*)	PS-NPs (0.4-0.6 μm) with and without fluoxetine (FLX)	PS-NPs (25, 100 particles/L)<br>FLX (5, 40 ng/L)	In water for 24 and 96 hpf	• Apoptosis-related genes (*baxa, casp3*) were upregulated, anti-apoptotic gene *bcl2* downregulated, and FLX-MPs mixtures caused stronger gene expression changes than single exposures, suggesting synergistic effects on apoptotic and stress pathways	Orozco-Hernández et al. [31]
Zebrafish embryos (*Danio rerio*)	PS-MPs (5 μm) and PS-NPs (100 nm) with and without silver nanoparticles (Ag-NPs; 5 nm)	PS MPs/NPs (200 μg/L) and AgNPs (10 μg/L)	In media for ~120hpf	• Neurodevelopmental and vascular genes (*elavl3*, *pax6a*, *egr2b*, *kdrl*, *fli1a*) were downregulated by AgNP-containing exposures<br>• Apoptosis (*casp3*, *casp9*) and inflammatory genes (*tnfa*) were strongly upregulated, especially in NP treatments	Song et al. [32]
Zebrafish embryos (*Danio rerio*)	PS-NPs, anionic carboxyl PS-NPs-COOH, and cationic amino PSNPs-NH_2 (100 nm) combined with and without acrylamide (ACR)	Single exposures at 48h-LC_{50} -<br>AR (200 mg/L),<br>PS-NPs (150 mg/L),<br>PS NPs-COOH (15 mg/L),<br>PS NPs-NH_2 (0.25 mg/L),<br>combined exposures of ACR at LC_{50} + PS NPs, PS NPs-COOH or PS NPs-NH_2 at 1/3 LC_{50}, 2/3 LC_{50}, LC_{50}	In a 5 mL solution for 120 hpf	• Exposure to ACR and PS NPs significantly altered the expression of CNS development–related genes (*Neurog1, Elavl3, Gfap*), indicating early neurotoxicity in zebrafish embryos<br>• ACR alone strongly suppressed *Neurog1*, *Elavl3*, and *Gfap* expression, indicating broad inhibition of neuronal differentiation and astrocyte function, whereas co-exposure with PS-NPs partially mitigated ACR effects on	Yang et al. [65]

				Neurog1 and *Gfap* expression, suggesting compensatory or antagonistic interactions<br>• Analysis of motor neuron development genes (*Gap43, Mbpa, Shha*) further supported neurodevelopmental disruption following exposure	
Zebrafish embryos and larvae (*Danio rerio*)	PS-NPs (25 ± 0.6 nm) combined with and without ER antagonist ICI	PS-NPs (0.01, 0.1, 1, and 10 μg/mL) and ICI (10 μM)	In embryo medium for 2 - 120 hpf	• Dopaminergic gene expression (120 hpf): PSNP exposure reduced *th1*, *th2*, *dat*, and *ddc* expression, which was reversed by co-incubation with ICI to control levels<br>• Apoptotic and anti-apoptotic gene expression (120 hpf): PSNP exposure increased *tp53*, *casp3*, *casp9*, and *bax* expression and decreased *bcl2a* expression, which was reversed by co-incubation with ICI to control levels	Saputra et al. [66]
Zebrafish larvae (*Danio rerio*)	PS-NPs (80 nm) combined with 2,2 ‘,4,4 ‘-tetrabromodiphenyl ether (BDE-47)	PS-NPs (0.05, 0.1, 1, 5, 10 mg/L), BDE-47 (0.1 and 10 μg/L)	In water for 120 hpf	• Antioxidant gene *gpx1a* transcription was downregulated in all exposure groups<br>• CYP1A1 were upregulated (0.05 mg/L PS-NPs +0.1 μg/L BDE-47 and 0.1 mg/L PS-NPs +0.1 μg/L BDE-47 co-exposure)	Wang et al. [34]
Zebrafish larvae (*Danio rerio*)	Aged PS-MPs (1 μm) and Thiamethoxam (TMX)	Aged PS-MPs (1 μg/L) TMX (1.5 μg/L)	In water for 120 hpf	• Co-exposure altered the expression of genes related to OS (e.g., *gstr1, sod1, cat1, gpx1a, ho-1, nrf2b*) and neurotransmission (*5-HT, ACh, and GABA* pathway genes).	Sun et al. [35]
Zebrafish larvae (*Danio rerio*)	PS-NPs (80 nm) combined with and without MEHP	PS-NPs (10, 25 and 50 μg/mL) and MEHP (10^{-6} and 10^{-5} M)	In embryo media for 24 hpf and 7 dpf	• Apoptosis-related gene expression: *p53* expression increased by 1.2-fold (NP25) and 1.5-fold (MN25), while *caspase-3* mRNA increased 1.35-fold in the MN25 group<br>• Neurodevelopmental gene expression: Co-exposure groups showed altered neural gene expression, with *gfap* upregulated, *mbp* downregulated, and *elavl3* showing a decreasing trend, suggesting impaired glial function and myelination<br>• OS gene response: Expression of the OS–related gene *keap-1* was significantly upregulated following exposure	Liu et al. [36]
Zebrafish larvae and juveniles (*Danio rerio*)	PS-NPs (250 nm) with and without methylmercury (MeHg)	1000 μg/L NPs, 1 μg/L MeHg (MeHg1), 10 μg/L MeHg (MeHg10), or their respective combinations (Mix1 and Mix10)	In water for 30 days	Gene ontology (GO) analysis indicated exposure-specific biological process alterations:<br>• NPs: Only one altered process (peptidyl-serine dephosphorylation)<br>• Mix1: Predominant effects on eye development and vision-related proteins<br>• MeHg1: Diverse impacts including adenosine metabolism, antioxidant activity, muscle development, and visual function<br>• MeHg10: Strong alterations in antioxidant defense, detoxification pathways, catabolism, and sensory development<br>• Mix10**:** Disruption of neurotransmitter transport and secretion, synaptic function, cell signaling, catabolism, and visual processes	Oger et al. [37]
Zebrafish (*Danio rerio*)	Polymer MPs (1–5 μm) with and without Cu	MPs (2 mg/L) $CuSO_4 \cdot 5H_2O$ (25 μg/L)	In water for 30 days	• Serotonergic disruption: *tph1a* was significantly upregulated in MPs and Cu25 + MPs, indicating enhanced 5-HT synthesis potentially related to stress<br>• Apoptotic activation: *casp3* was upregulated in Cu25 + MPs, with a trend toward increased *casp8* and no changes in *casp9* or *aif*, suggesting apoptosis	Santos et al. [42]

Zebrafish (*Danio rerio*)	PS-NPs (100 nm) with and without and arsenic (As)	PS-NPs (1 mg/L)<br>As (1 mg/L)	In water for 30 days	• MAO mRNA was downregulated in the NP + As group<br>• Tryptophan hydroxylase (TPH) genes *tph1a*, *tph1b*, and *tph2* were downregulated in the co-exposure group, indicating suppressed 5-HT synthesis<br>• 5-HT receptor expression in brain: co-exposure increased *htr1aa*, *htr1ab*, and *htr2c* mRNA levels and *htr1b* and *htr4* were decreased	Zhang et al. [43]
Zebrafish (*Danio rerio*)	PS-NPs (44 nm), PS-NPs fluorescently labeled (42 nm), anionic carboxyl PS-NPs-COOH (51 nm), and cationic amino PSNPs-NH_2 (51 nm)	10 µg/L	In water for 120 days (M and F spawned on day 113 to obtain F1 embryos for analysis)	Male zebrafish brain gene expression:<br>• PS exposure: Downregulation of GAP, TNFα, and IL-13; upregulation of GFAP and Mafbb<br>• PS-NH_2 exposure: Downregulated GAP, GFAP, BDNF, TNFα, and IL-13; upregulated Mafbb and IL-4.<br>• PS-COOH exposure: Increased IL-4 and IL-13; decreased GFAP, TNFα, and Mafbb<br>• Compared to PS: PS-NH_2 increased Mafbb and IL-13 but decreased GFAP, TNFα, and IFN; PS-COOH upregulated GAP, GFAP, IL-4, IL-13 and downregulated TNFα, Mafbb<br>Female zebrafish brain gene expression:<br>• PS exposure: Downregulation of TNFα and IFN; upregulation of Mafbb, IL-4, IL-13.<br>• PS-NH_2 exposure: Upregulated *GFAP, Mafbb, IL-4, IL-13*; downregulated *IFN*<br>• PS-COOH exposure: Upregulated IFN and Mafbb; downregulated *IL-4* and *IL-13*.<br>• Compared to PS: PS-NH_2 upregulated GFAP and TNFα, downregulated IFN, IL-4, IL-13; PS-COOH increased TNFα and IFN, decreased Mafbb, IL-4, IL-13<br>L-4 and IL-13 expression in zebrafish brains showed sex-specific responses after PS-COOH exposure:<br>• Males: Upregulation of IL-4 and IL-13<br>• Females: Downregulation of IL-4 and IL-13	Teng et al. [68]
Zebrafish (*Danio rerio*)	PS; 0.6-1.0 µm	25 and 250 µg/L	In water for 40 days	Inflammatory responses in the zebrafish brain:<br>• 25 µg/L PS-MP exposure led to a 4.0-fold upregulation of *il-6*<br>• 250 µg/L PS-MP exposure induced a 1.9-fold upregulation of *il-1β*<br>Upregulation of circadian rhythm genes<br>• 14 genes including *per1a, per1b, per2, per3, cry2, cry5*, were upregulated<br>Increased microglial activation<br>• general microglial activation marker *CD68* (2.2-fold)<br>• M1 phenotype marker *CD16* (9.3-fold)	Yang et al. [69]

Goldfish (*Carassius auratus*)	PS-MPs (30 µm) and PS-NPs (500 nm)	0.26 mg/L (low) and 0.69 mg/L (high)	In water for 28 days	• MP exposure altered neurotransmitter-related genes in a size- and dose-dependent manner<br>• *DAT* and *ChAT* were downregulated, *GAD2* was upregulated at higher exposures, and GPCR-related olfactory receptor genes were broadly suppressed	Shi et al. [70]
Channel catfish (*Ictalurus punctatus*)	PS-NPs (80 nm) and BPA with and without Biochar	PS-NPs (0.5 mg/L) BPA (0.2 mg/L) Biochar (0.5 g/L)	In water for 7 days	Pituitary Adenylate Cyclase-Activating Polypeptide (PACAP) expression (brain):<br>• Down-regulated in PS group<br>• Strongly down-regulated in BPA group pro-opiomelanocortin expression (brain):<br>• Up-regulated in PS group r*ab3a* expression (brain):<br>• Up-regulated in PS group	Zheng et al. [50]
Spotted snakehead (*Channa punctatus*)	PVC-MPs (0.315 µm) with and without Copper sulfate pentahydrate ($CuSO_4 \cdot 5H_2O$)	PVC-MPs (0.5 mg/L), Copper (0.85 mg/L), and a co-exposure	In water for 15, 30, 45, and 60-days	• mRNA expression of apoptosis-related genes was analyzed after 60 days<br>• Anti-apoptosis genes (*bdnf*, *miR132*, *bcl2*) were downregulated<br>• Pro-apoptotic genes (*foxo3a, bim, bax, apaf1, casp9, casp3*) were upregulated	Bakhasha et al. [56]

Table S5. Behavioral alterations in aquatic invertebrates and vertebrates following acute and chronic exposure to MPs and NPs.

Species	Plastic/size	Exposure Dose(s)	Exposure Method	Behavioral Effect(s)	Reference
**Invertebrates**					
Polychaete (*Hediste diversicolor*)	PET-MPs (125 µm to 1 mm) with and without the antibiotic CIP and industrial effluents	MPs (0.032 g/L and 0.054 g/L) CIP (130 ng/L and 1300 ng/L) Industrial effluents (3%)	In sea water for 28 days	• Burial behavior was altered among groups<br>• Polychaetes exposed to CIP alone (both concentrations) and CIP combined with low MP density buried faster than controls<br>• Burrowing time was reduced in 3% effluent + low CIP and 3% effluent + high CIP + low MP groups<br>• Exposure to low CIP plus low MPs reduced burrowing time relative to the effluent group only<br>• High MP density increased burrowing time, with the 3% effluent + high CIP + high MP group showing slower burial	Araújo et al. [2]
Water flea (*Daphnia magna*)	Tire wear particles (TWP) MPs (0.7-70 µm) and leachate extraction from TWP for 30 and 60 min (E-30 and E-120)	TWP, 30-E, and 120-E (50, 100, 200, 400, 600, and 800 mg/L each)	In culture media for 24 and 48 h	• Both TWP and leachates reduced swimming speed, acceleration, and activity range, even at low concentrations<br>• Filtration and ingestion rates were reduced by particles and declined further with increasing leachate concentrations	Liu et al. [4]
Freshwater bivalves (*Corbicula fluminea*)	PS-MPs (6 µm) and PS-NPs (80 nm) with and without the antibiotic CIP	PS-MPs and PS-NPs (10 µg/g dw); CIP (0.5 µg/g, 5 µg/g, and 50 µg/g dry weight (dw))	In sediment for 10 days	• Filtration rates decreased in a concentration-dependent manner for all treatments<br>• A correlation was found between reduced AChE activity and decreased filtration rate, confirming that neurotoxicity affects siphon behavior	Guo et al. [11]
Freshwater bivalves (*Corbicula fluminea*)	Commercial PS-NPs (200 nm) and NPs generated from macro-sized plastics collected in the field (ENV-NPs; 235 ± 70 nm)	Microalgae were contaminated for 48 h with 0.008, 10 and 100 µg/L PS-NPs and ENV-NPs	Bivalves were then fed with the contaminated algae every 48 h for 21 days	• After 21 days, burrowing speed was higher in bivalves exposed to PS-NPs at 100 µg/L<br>• At 10 and 100 µg/L, exposure to ENV-NPs resulted in more impaired burrowing capacity than exposure to PS-NPs<br>• For PS-NPs, only the lowest concentration (0.008 µg/L) impaired burrowing behavior relative to the	Latchere et al. [12]

				higher concentrations (10 and 100 μg/L)	
**Vertebrates**					
Tadpoles (*Rana nigromaculata)*	PS-MPs 0.1 μm (MP1), 1 μm (MP2), and 10 μm (MP3)) with and without levofloxacin (LVFX)	MPs (0.10 mg/L)<br>LVFX (1.00 μg/L)	In water for 45 days	• Abnormal behaviors increased in tadpoles exposed to LVFX–MP1 and LVFX–MP3, with higher proportions of swirling behavior and feeding activity<br>• Overall activity and tail-beat frequency were reduced in LVFX–MP1 and LVFX–MP3 treatments<br>• The LVFX–MP3 group showed a higher proportion of tadpoles lying laterally or dorsally, indicating pronounced behavioral impairment<br>• Tadpoles in LVFX–MP3 spent more time in the peripheral area of the tank, indicative of altered spatial behavior<br>• Air surface breathing increased markedly after co-exposure with MPs, particularly in LVFX–MP1 and LVFX–MP3, suggesting enhanced stress responses<br>• Vertical distribution shifted with exposure: control tadpoles primarily occupied the bottom layer, while LVFX–MP1 and LVFX–MP3 groups showed increased surface occupancy<br>• Behavioral impact severity followed the order: LVFX–MP3 > LVFX–MP1 > LVFX–MP2 ≥ LVFX	Zhang et al. [26]
Zebrafish embryos (*Danio rerio*)	PLA BioMPs (2.34 ± 0.07 μm)	2.5 mg/L (BP I) and 5 mg/L (BP II)	In water for 30 days	Social interaction with non-predatory fish:<br>• Control fish remained closer to the heterospecific stimulus and showed higher interaction frequency<br>• Shoals exposed to BP II exhibited increased cohesion<br>• A concentration-dependent increase in distance from the non-predator was observed with increasing PLA BioMP exposure<br>Response to simulated terrestrial predator (mouse):<br>• Control shoals preferentially occupied the middle zone, BP I shoals stayed closer to the surface, and BP II shoals remained predominantly in the lower zone<br>• Shoal cohesion was highest in the BP II group<br>• Control fish displayed significantly more explosive escape behaviors compared to both exposed groups	Chagas et al. [59]
Zebrafish embryos (*Danio rerio*)	PS-NPs (100 nm) with and without Avobenzone (AVO)	NPs (10 μg/L)<br>AVO (10 μg/L)	In media for 144 h followed by a 72-h recovery in dH_2O	• All exposure groups showed altered swimming trajectories, reduced swimming space, and circular movements<br>• Swimming speed was reduced by AVO, NPs, and co-exposure, with stronger effects from NPs and co-exposure<br>• Behavioral impairments improved after recovery but did not fully return to control levels, indicating lasting neurobehavioral effects	Liu et al. [28]
Zebrafish embryos (*Danio rerio*)	PS-NPs (-NH_2; 51 nm) and PS-NPs (-COOH; 50 nm)	PS-NH_2 and PS-COOH (30 and 50 mg/L)	In water for 24, 48, 72, 96, and 120 hpf	• PS-NH2 significantly reduced swimming velocity, acceleration, distance traveled, and active time, while increasing inactivity<br>• PS-COOH did not produce comparable locomotor deficits	Teng et al. [61]
Zebrafish embryos (*Danio rerio*)	PS; 25 nm	10, 25, and 50 mg/L	In water for 4-100 hpf	• Touch-evoked escape response (TEER; % of embryos showing C-	Lin et al. [62]

				curvature) decreased at 25 and 50 mg/L<br>• Maximum velocity of TEER decreased at 50 mg/L<br>• Maximum acceleration of TEER decreased at 25 and 50 mg/L<br>• No changes observed at 10 mg/L	
Zebrafish embryos (*Danio rerio*)	Virgin and aged PS-MPs (1 µm)	0.1, 1, 10, and 100 µg/L	In media for 120 hpf	• Both virgin and aged PS-MPs reduced swimming speed and distance in zebrafish larvae<br>• Hypoactivity was concentration-dependent and more pronounced with aged PS-MPs<br>• Aged PS-MPs affected behavior across both light and dark cycles<br>• Toxicity of aged PS-MPs was greater than that of virgin PS-MPs	Xiang et al. [64]
Zebrafish embryos (*Danio rerio*)	PMF from facial mask (surgical and N95) leachate;<br>• Surgical – length 112.7 ± 57.12 µm and width of 4.28 ± 1.16 µm (PP fibers plus aluminum (Al) and calcium (Ca))<br>N95 – length 7.89 ± 11.69 µm and width of 2.15 ± 0.50 µm (PET fibers and Al, Ca, silicon (Si), chlorine (Cl), and potassium (K))	1000, 10,000, and 100,000 particle/L	In exposure media for 144 h	• Behavioral responses were mask-dependent, with N95 mask PMFs inducing stronger effects than surgical mask PMFs<br>• Exposure to N95 mask PMFs at higher concentrations (10,000 and 100,000 particles/L) significantly increased mean swimming velocity compared to controls and surgical mask treatments<br>• Total distance traveled increased in larvae exposed to surgical mask PMFs (10,000 and 100,000 particles/L) and in all N95-mask PMF treatments<br>• Freezing behavior did not differ across all treatments<br>• PMF-exposed larvae showed increased activity and agitation and exhibited thigmotaxis, avoiding the center of the arena and remaining near the edges compared with controls	Qualhato et al. [29]
Zebrafish embryos (*Danio rerio*)	Virgin and photoaged PS (P-PS) (1 µm) with and without adsorbed clothianidin (CLO)	PS-MPs (100 µg/L)<br>CLO (3 µg/L)	In water for 5 days	• CLO alone and CLO co-exposed with PS or photoaged PS (P-PS) mainly induced short-term alterations in swimming performance and stress responses during light–dark transitions<br>• PS + CLO exposure significantly reduced swimming speed during dark phases compared to CLO alone<br>• P-PS + CLO exposure caused a more pronounced reduction in swimming speed, occurring during both light and dark phases<br>• Swimming speed in the P-PS + CLO group was significantly lower than in the PS + CLO group<br>• Overall, results indicate that photoaging enhances the neurotoxic effects of MPs when co-exposed with CLO	Ding et al. [73]
Zebrafish embryos (*Danio rerio*)	PS-NPs (25 ± 0.6 nm) combined with and without Estrogen Receptor antagonist ICI 182,720 (ICI)	PS-NPs (0.01, 0.1, 1, and 10 µg/mL) and ICI (10 µM)	In embryo medium for 2 - 120 hpf	• Tactile responsiveness: PS-NP exposure (0.1–10 µg/mL) caused a significant, dose-dependent reduction in tactile responses, which was reversed by co-incubation with 10 µM ICI, restoring responses to control levels.<br>• Cross-line response (120 hpf): PS-NP exposure (0.1–10 µg/mL) caused a significant, dose-dependent decrease in cross-line responders, which was reversed by co-incubation with 10 µM ICI to control levels<br>• Anxiety-like behavior (thigmotaxis, 120 hpf): PS-NP exposure significantly increased time spent in the edge zone under both dark and light conditions, which was reversed by co-incubation	Saputra et al. [66]

				with 10 μM ICI to levels comparable to controls<br>• Swimming velocity (120 hpf): PS-NP exposure caused a significant, concentration-dependent decrease in swimming velocity over 20 minutes, with the greatest reduction at 10 μg/mL, which was restored to control levels by co-incubation with 10 μM ICI.<br>• Total movement distance (120 hpf): PS-NP exposure (0.1–10 μg/mL) significantly reduced total distance traveled, and co-incubation with 10 μM ICI restored distances to control levels<br>• Average swimming speed (120 hpf): PS-NP exposure induced a significant, dose-dependent decrease in average swimming speed, which was reversed by co-incubation with 10 μM ICI to control levels.	
Zebrafish embryos (*Danio rerio*)	PS-MPs (5 μm) and PS-NPs (100 nm) with and without silver nanoparticles (Ag-NPs; 5 nm)	PS MPs/NPs (200 μg/L) and AgNPs (10 μg/L)	In media for ~120hpf	• AgNPs, MP, and NP exposure reduced locomotor activity, increased immobility, altered turning angles, and impaired responses to light–dark transitions, sound, and tactile stimuli<br>• Combined exposures, especially NP, produced anxiety-like behaviors and delayed escape responses	Song et al. [32]
Zebrafish embryos (*Danio rerio*)	PS-NPs, anionic carboxyl PS-NPs-COOH, and cationic amino PSNPs-NH_2 (100 nm) combined with and without ACR	Single exposures at 48h-LC_{50} -<br>AR (200 mg/L),<br>PS-NPs (150 mg/L),<br>PS NPs-COOH (15 mg/L),<br>PS NPs-NH_2 (0.25 mg/L), combined exposures of ACR at LC_{50} + PS NPs, PS NPs-COOH or PS NPs-NH_2 at 1/3 LC_{50}, 2/3 LC_{50}, LC_{50}	In a 5 mL solution for 120 hpf	• Only positively charged PS NPs–NH_2 significantly increased total distance traveled and swimming speed, indicating a stronger impact on larval locomotor activity compared with PS NPs–COOH<br>• In combined exposure treatments (ACR + PS NPs), most locomotor parameters (distance, speed, and acceleration) were elevated, with the exception of acceleration in the PS NPs–COOH + ACR group<br>• Although co-exposure did not significantly differ from PS NPs–NH_2 alone, an overall upward trend in locomotor activity suggested that joint exposure may exacerbate neurobehavioral toxicity<br>• Altered swimming trajectories were observed in all exposed groups, characterized by restricted movement, circling behavior, and edge-preference, whereas control larvae displayed evenly distributed movement patterns<br>• These abnormal trajectories were more pronounced in co-exposure groups, indicating enhanced neurobehavioral disruption when ACR and PS NPs were present together	Yang et al. [65]
Zebrafish embryos (*Danio rerio*)	PS-MPs (5 μm) and PS-NPs (60 nm)	0.05–50 mg/L	In water for 7dpf	• PS-NPs suppressed spontaneous tail-coiling at 50 mg/L, whereas PS-MPs had no effect<br>• In dark exploration tests, PS-NPs increased swimming distance at early stages (5–6 dpf), while PS-MPs induced a dose-dependent increase in motility across concentrations<br>• Behavioral alterations diminished by 7 dpf for both particle types<br>• In light exploration tests, PS-NPs had no effect on swimming distance, while PS-MPs showed a trend toward reduced swimming at later stages<br>• In light–dark stimulation assays, PS-NP–exposed larvae retained normal light–dark rhythms but exhibited reduced swimming speeds	Li et al. [33]

				• PS-MP–exposed larvae lost normal light–dark rhythmicity and displayed hyperactivity under light stimulation, especially at 50 mg/L	
Zebrafish larvae (*Danio rerio*)	PLABioMPs (2.34 ± 0.07 μm)	3 and 9 mg/L	In water for 5 days	• PLA BioMPs exposure reduced locomotor activity, evidenced by shorter distance moved and lower swimming speed<br>• Anxiety-like behavior increased, indicated by enhanced thigmotaxis and more frequent freezing events	De Oliveira et al. [74]
Zebrafish larvae (*Danio rerio*)	Polymer MP (1–5 μm) with and without copper sulfate pentahydrate	MPs (2 mg/L)<br>Cu (60 and 125 μg/L)	In water for 14 days	• Mean distance to the center of the well did not differ among High Cu exposure, especially when combined with MPs, caused locomotor hypoactivity and impaired behavioral responses to aversive stimuli<br>• Social (shoaling) behavior remained intact, indicating selective effects on locomotion and cognition rather than group cohesion	Santos et al. [75]
Zebrafish larvae (*Danio rerio*)	PS-NP (30 nm)	PS-NPs (0.1, 0.5, and 3 mg/L)	In water for 120 hpf	• At 24 hpf, tail activity (bursts/min) increased in a concentration dependent manner<br>• Mean burst duration (burst quality) decreased in all treatments<br>• During the dark interval, there was an increase in larvae activity at 0.1 and 0.5 mg/L and a decrease for larvae exposed to 3 mg/L<br>• During the light period there was a decrease in movement for larvae at 0.1 mg/L and an increase at 0.5 mg/L and 3 mg/L<br>• Thigmotaxis behavior (anxiety) during visual stimulation showed that during dark periods, increased anxiety at 0.1 and 0.5 mg/L with less time in the well center and more time at the edge<br>• During the light period, embryos spend less time in the well center and more near the edge<br>• Larvae exposed to 3 mg/L spent more time at the well center and less time at the edge during both light and dark periods (decreased anxiety)<br>• Anxiolytic effects following auditory stimulation (tapping noises): larvae exposed to 0.5 and 3 mg/L exhibited more time in well centers and less at the edge<br>• In response to a first loud auditory stimulus, larvae exposed to 0.1 and 0.5 mg/L PS-NP showed increased reaction (more distance traveled) during the first jump	Torres-Ruiz et al. [63]
Zebrafish larvae (*Danio rerio*)	• PS-NPs (80 nm) combined with 2,2 ‘,4,4 ‘-tetrabromodiphenyl ether (BDE-47)	PS-NPs (0.05, 0.1, 1, 5, 10 mg/L)<br>BDE-47 (0.1 and 10 μg/L)	In water for 120 hpf	• Spontaneous movements in PS-NPs single exposure group showed a decreasing trend with increasing PS-NPs<br>• PS-NPs + BDE-47 co-exposure, all co-exposures also showed a decreasing trend in spontaneous movements with increasing PS-NPs concentration<br>• PS-NPs + BDE-47 (0.1 and 10 μg/L) co-exposure increased spontaneous movements	Wang et al. [34]
Zebrafish larvae (*Danio rerio*)	Aged PS-MPs (1 μm) and TMX	Aged PS-MPs (1 μg/L)<br>TMX (1.5 μg/L)	In water for 120 hpf	• Swimming velocity was reduced by aged PS and TMX individually and was most strongly suppressed in the combined exposure, demonstrating synergistic impairment of locomotor activity	Sun et al. [35]
Zebrafish larvae (*Danio rerio*)	PS-NPs (100 nm) with and without PCB-153	PS-NPs (1.05 g/mL) PCB-153 (0.25, 0.5, 1, 2, 4, 8, and 16 μg/mL)	In E3 medium for 96h	• Larvae exposed to NPs + PCB exhibited pronounced hyperlocomotion, with increased	Varshney et al. [67]

				distance moved, velocity, and total movement<br>• NPs or PCB alone did not affect locomotion<br>• Clockwise rotation frequency increased in all treatment groups	
Zebrafish larvae (*Danio rerio*)	PS-MPs and PS-NPs (0.05, 0.25, 0.53, 2.1, 6.02, and 10.2 µm)	0.0005 to 0.2µg/µL	In exposure media for 120 hpf	• Behavioral tracking revealed clear deviations from control larvae, particularly at the highest MP concentration<br>• Behavioral toxicity was predominantly expressed as reduced baseline locomotor activity across particle sizes<br>• Decreased dark response was most evident for larger MP during the General and Behavioral Toxicity assay<br>• The smallest particles (0.05 µm) showed the greatest sensitivity and variability in behavioral<br>• Larger particles (≥2.1 µm) generally elicited behavioral effects only at the highest tested concentration in both assays	Levesque et al. [80]
Zebrafish larvae (*Danio rerio*)	PS-NPs (80 nm) combined with and without MEHP	PS-NPs (10, 25 and 50 µg/mL) and MEHP (10^{-6} and 10^{-5} M)	In embryo media for 24 hpf and 7 dpf	• Autonomous movement (10 min darkness): Co-exposure to PS-NPs (NP10, NP25, NP50) and 10^{-5} M MEHP significantly reduced average speed by 36.6%, 53.0%, and 34.4%, respectively<br>• Movement distance: Co-exposure decreased displacement distance, with NP25 + 10^{-5} M MEHP showing the greatest reduction<br>• Cumulative movement duration: High-concentration MEHP enhanced the neurobehavioral toxicity of PS-NPs, affecting both moving and non-moving durations<br>• Individual exposures: PS-NPs or MEHP alone did not alter average speed, total movement distance, or cumulative movement duration<br>• Total swimming distance (25 min light/dark stimulation, 7 dpf): NP25 and MN25 groups showed significantly increased swimming distance during all dark stages compared to controls<br>• Movement distance under light/dark cycles: MN25 larvae moved less than controls in both light and dark periods<br>• Swimming speed: Transition from dark to light caused rapid speed decrease; NP25 and MN25 larvae swam slower than controls, with MN25 showing the lowest speed<br>• Immobility: During the light period, immobility increased by 2.2% in MN25; during the dark period, immobility increased by 2.84% (NP25) and 2.1% (MN25)	Liu et al. [36]
Zebrafish larvae and juveniles (*Danio rerio*)	PS-NPs (250 nm) with and without methylmercury (MeHg)	1000 µg/L NPs, 1 µg/L MeHg (MeHg1), 10 µg/L MeHg (MeHg10), or their respective combinations (Mix1 and Mix10)	In water for 30 days	• Visual motor response differed by developmental stage, light condition, and exposure type in zebrafish larvae and juveniles<br>• NP exposure caused a slight, reduction in larval activity<br>• High-dose MeHg (MeHg10) and Mix10 reduced larval swimming activity in both light and dark conditions<br>• NP exposure increased juvenile activity, with an increase in the dark	Oger et al. [37]

				• MeHg1 did not affect juvenile swimming behavior in either light or dark conditions<br>• Mix1 reduced juvenile activity by ~16.5% in both light and dark<br>• MeHg10 and Mix10 caused marked hypoactivity in juveniles, decreasing swimming distance in both light and dark conditions	
Zebrafish larvae and adults (*Danio rerio*)	Schwarzbach River water samples containing PE (50%), PP (25%), PS (15%), and PVC (10%) and sorbed contaminants (up to 94 different compounds)	100 mg/L (except larval behavior tests 5 mg/L)	In river water for 24, 48, 72, and 96 hpf	Schwarzbach water exposure:<br>• Increased larval swimming activity compared to controls<br>• Caused an increase in activity during the final dark phase<br>• Led to slight hyperactivity and reduced adaptation to light–dark transitions	Hanslik et al. [40]
Zebrafish (*Danio rerio*)	PS-NPs (70 nm)	PS-NPs (0.5 and 1.5 ppm)	In water for 7 days	• Fish (1.5 ppm) exhibited hyperactivity-like behavior, evidenced by increased average swimming speed and reduced freezing time<br>• Increased top-zone entries and distance traveled, indicating altered exploration<br>• Locomotion paths and behavioral alterations differed in the high-concentration group<br>• Both PS-NPs doses reduced aggression, shown by less mirror biting and time near the mirror<br>• PS-NP- exposed fish showed reduced locomotion, including decreased average swimming speed, swimming time movement ratio, and rapid movement ratio<br>• Freezing behavior increased in PS-NPs–exposed groups<br>• Zebrafish (1.5 ppm) showed a reduced average distance from the separator, indicating diminished avoidance of the predator<br>• Chronic high-dose PS-NPs exposure disrupts normal circadian locomotor rhythms in zebrafish, inducing hypoactivity and altered movement patterns during both light and dark cycles	Sarasamma et al. [41]
Zebrafish (*Danio rerio*)	PS-NPs (100 nm) with and without and arsenic (As)	PS-NPs (1 mg/L)<br>As (1 mg/L)	In water for 30 days	• Locomotor activity: co-exposure (NPs + As) caused reduced swimming activity<br>• Anxiety-like behavior: co-exposed zebrafish spent more time in the lower layer, indicating increased anxiety-like behavior<br>• Learning and memory: As and NPs + As groups displayed impaired learning/memory	Zhang et al. [43]
Zebrafish (*Danio rerio*)	PS-NPs (44 nm), PS-NPs fluorescently labeled (42 nm), anionic carboxyl PS-NPs-COOH (51 nm), and cationic amino PSNPs-NH_2 (51 nm)	10 µg/L	In water for 120 days (M and F spawned on day 113 to obtain F1 embryos for analysis)	• Exposure to PS in adult zebrafish led to reduced locomotor activity, including decreased active time, swimming velocity, and total distance traveled<br>• PS-NH_2 and PS-COOH exposure impaired cognitive performance in both male and female zebrafish, as shown by altered spatial preference and goal-zone performance<br>• Depressive-like behaviors were observed, with increased static duration in males exposed to PS and PS-COOH and in females exposed to PS-NH_2<br>• Social behavior was disrupted in males exposed to PS-COOH, evidenced by reduced social distance metrics	Teng et al. [68]

				• Offspring (F1 generation) of PS-COOH–exposed parents exhibited increased activity and swimming velocity, suggesting transgenerational effects of chronic PS exposure	
Zebrafish (*Danio rerio*)	PS; 0.6-1.0 µm	25 and 250 µg/L	In water for 40 days	Induction of depression-like behaviors<br>• impaired locomotion<br>• reduced movement<br>• increased freezing behavior<br>• reduced exploration of the light zone<br>• reduced average inter-individual distances	Yang et al. [69]
Goldfish (*Carassius auratus*)	PS-MPs (30 µm) and PS-NPs (500 nm)	0.26 mg/L (low) and 0.69 mg/L (high)	In water for 28 days	• MP exposure reduced goldfish activity in odorant-supplemented zones<br>• Behavioral impairment was size- and concentration-dependent, with the strongest suppression in fish exposed to high concentrations and NPs	Shi et al. [70]

ABBREVIATIONS

5-HT – Serotonin
8-OHdG – 8-hydroxy-2'-deoxyguanosine
ACh – Acetylcholine
AChE – Acetylcholinesterase
ACP – Acid phosphatase
ACR – Acrylamide
ALK – Alkaline phosphatase
ALT – Alanine aminotransferase
BBB – Blood brain barrier
BChE – Butirylcholinesterase
BFCOD – Benzyloxy-4-trifluoromethylcoumarin-O-debenzyloxylase
BioMPs – Biomicroplastics
BPA – Bisphenol A
CAT – Catalase
CAT-L – Catalase (liver)
CbE – Carboxylesterase
ChAT – Choline acetyltransferase
ChE – Cholinesterase
CIP – Ciprofloxacin
CNS – Central nervous system
CYP450 – Cytochrome P450
DA – Dopamine
dpf – Days post fertilization
DOPA – Levodopa
EROD – 7-Ethoxyresorufin-O-deethylase
EMPs – Environmental microplastics
ENV – Environmental
ENR – Enrofloxacin
FI – Fluorescence intensity
GABA – Gamma-Aminobutyric Acid
GFAP – Glial fibrillary acidic protein
GFP – Green fluorescent protein

GPx – Glutathione peroxidase
GR – Glutathione reductase
GSSG – Oxidized states of glutathione levels
GSH – Reduced glutathione
GSH-PX – Glutathione peroxidase
GST – Glutathione-S-transferase
HDPE – High-density polyethylene
hpf – Hours post fertilization
il-1β – Interleukin-1 beta
il-6 – Interleukin-6
LDPE – Low-density polyethylene
LPO – Lipid peroxidation
LYZ – Lysozyme
MAO – Monoamine oxidase
MDA – Malondialdehyde
MEHP – Mono-(2-ethylhexyl) phthalate
MET – Metolachlor
MP – Microplastic
MT – Metallothionein
NE – Norepinephrine
NEGR – Neuronal Growth Regulator
NO – Nitric oxide
NP – Nanoplastic
NPY – Neuropeptide Y
OS – Oxidative stress
OTC –oxytetracycline
p.s.u – Practical salinity unit
PA – Polyamide
PAA – Polyacrylic
PACAP – Pituitary Adenylate Cyclase-Activating Polypeptide
PAH – Polyaromatic hydrocarbons
PAN – Polyacrylonitrile
PCNA – Proliferating cell nuclear antigen
PE – Polyethylene
PEI – Polyethyleneimine
PES – Polyester
PET – Polyethylene terephthalate
PEVA – Polyethylene vinyl acetate
PFOA – Perfluorooctanoic acid
PG - Pyrogallol
PHX – Phenoloxidase
PLA – Polylactic acid
PMF – Plastic microfibers
PMMA – Polymethyl methacrylate
PP – Polypropylene
PRP – Propranolol
PS – Polystyrene
PS-COOH – Polystyrene Anionic Carboxyl
$PS\text{-}NH_2$ – Polystyrene Cationic Amino
PVC – Polyvinyl chloride
RF – Reticular formation

ROS – Reactive oxygen species
se-GPX – Selenium-dependent glutathione peroxidase
SMX – Sulfamethoxazole
SOD – Superoxide dismutase
TAC – Total antioxidant capacity
TCPP – tris(1-chloro-2-propyl) phosphate
TCS – Triclosan
TEM – Transmission electron microscopy
TF – Thifluzamide
TMX – Thiamethoxam
TNF-α – Tumor Necrosis Factor-alpha
TPH – Tryptophan hydroxylase
Trp – Tryptophan
Tyr – Tyrosine

REFERENCES

1. Mustapha, D.S.; Rodríguez-Díaz, O.; Cajaraville, M.P.; Orbea, A. PLA Nanoplastics Accumulate but Do Not Cause Acute Toxicity to Marine Rotifers, Brine Shrimps, and Zebrafish Embryos. *J. Xenobiotics* **2025**, *15*, 196, doi:10.3390/jox15060196.
2. Araújo, A.M.; Ringeard, H.; Nunes, B. Do Microplastics Influence the Long-Term Effects of Ciprofloxacin on the Polychaete *Hediste diversicolor*? An Integrated Behavioral and Biochemical Approach. *Environ. Toxicol. Pharmacol.* **2023**, *99*, 104088, doi:10.1016/j.etap.2023.104088.
3. Peixoto, D.; Torreblanca, A.; Pereira, S.; Vieira, M.N.; Varó, I. Effect of Short-Term Exposure to Fluorescent Red Polymer Microspheres on *Artemia franciscana* Nauplii and Juveniles. *Environ. Sci. Pollut. Res.* **2022**, *29*, 6080–6092, doi:10.1007/s11356-021-15992-y.
4. Liu, J.; Feng, Q.; Yang, H.; Fan, X.; Jiang, Y.; Wu, T. Acute Toxicity of Tire Wear Particles and Leachate to *Daphnia magna*. *Comp. Biochem. Physiol. Part C Toxicol. Pharmacol.* **2023**, *272*, 109713, doi:10.1016/j.cbpc.2023.109713.
5. Zhu, C.; Zhou, H.; Bao, M.; Tang, S.; Gu, X.; Han, M.; Li, P.; Jiang, Q. Polystyrene Microplastics Induce Molecular Toxicity in *Simocephalus vetulus*: A Transcriptome and Intestinal Microorganism Analysis. *Aquat. Toxicol.* **2024**, *275*, 107046, doi:10.1016/j.aquatox.2024.107046.
6. Eom, H.-J.; Haque, Md.N.; Lee, S.; Rhee, J.-S. Exposure to Metals Premixed with Microplastics Increases Toxicity through Bioconcentration and Impairs Antioxidant Defense and Cholinergic Response in a Marine Mysid. *Comp. Biochem. Physiol. Part C Toxicol. Pharmacol.* **2021**, *249*, 109142, doi:10.1016/j.cbpc.2021.109142.
7. Gholamhosseini, A.; Banaee, M.; Zeidi, A.; Multisanti, C.R.; Faggio, C. Individual and Combined Impact of Microplastics and Lead Acetate on the Freshwater Shrimp (*Caridina fossarum*): Biochemical Effects and Physiological Responses. *J. Contam. Hydrol.* **2024**, *262*, 104325, doi:10.1016/j.jconhyd.2024.104325.
8. Bertrand, L.; Yacelga Villavicencio, N.C.; Rimondino, G.N.; Gonzalez, M.F.; Amé, M.V. Roles of Bio-Based Microplastics in Modulating the Toxic Effects of the Herbicide Metolachlor on the South American Native Species *Palaemon argentinus*: Single and Co-Exposure Effects. *Aquat. Toxicol.* **2025**, *287*, 107532, doi:10.1016/j.aquatox.2025.107532.
9. Hamed, M.; Said, R.E.M.; Shaalan, W.M.; Elbaghdady, H.A.M.; Sayed, A.E.-D.H. Immunological, Neurological, and Intestinal Changes in Red Swamp Crayfish

(*Procambarus clarkii*) Exposed to the Combined Toxicity of Pyrogallol and Microplastics. *Mar. Pollut. Bull.* **2025**, *213*, 117641, doi:10.1016/j.marpolbul.2025.117641.
10. Sbarberi, R.; Magni, S.; Ponti, B.; Tediosi, E.; Neri, M.C.; Binelli, A. Multigenerational Effects of Virgin and Sampled Plastics on the Benthic Macroinvertebrate *Chironomus riparius*. *Aquat. Toxicol.* **2025**, *279*, 107205, doi:10.1016/j.aquatox.2024.107205.
11. Guo, X.; Cai, Y.; Ma, C.; Han, L.; Yang, Z. Combined Toxicity of Micro/Nano Scale Polystyrene Plastics and Ciprofloxacin to *Corbicula fluminea* in Freshwater Sediments. *Sci. Total Environ.* **2021**, *789*, 147887, doi:10.1016/j.scitotenv.2021.147887.
12. Latchere, O.; Métais, I.; Perrein-Ettajani, H.; Lemoing, M.; Feurtet-Mazel, A.; Gonzalez, P.; Daffe, G.; Gigault, J.; Catrouillet, C.; Châtel, A.; et al. Trophic Transfer Effects of PS Nanoplastics and Field-Derived Nanoplastics in the Freshwater Clam *Corbicula fluminea*. *Aquat. Toxicol.* **2024**, *277*, 107160, doi:10.1016/j.aquatox.2024.107160.
13. Secco, S.; Cunha, M.; Leite, C.; Libralato, G.; Trifuoggi, M.; Giarra, A.; Soares, A.M.V.M.; Freitas, R.; Scalici, M. Breaking New Ground: Gadolinium and Microplastics Co-Exposure and Biochemical Alterations in Marine Clam *Donax trunculus*. *Aquat. Toxicol.* **2025**, *286*, 107394, doi:10.1016/j.aquatox.2025.107394.
14. Moncrieffe, R.; Masry, M.; Cai, B.; Rossignol, S.; Kamari, A.; Poirier, L.; Bertrand, S.; Wong-Wah-Chung, P.; Zalouk-Vergnoux, A. Study of the Ageing and the Sorption of Polyaromatic Hydrocarbons as Influencing Factors on the Effects of Microplastics on Blue Mussel. *Aquat. Toxicol.* **2023**, *262*, 106669, doi:10.1016/j.aquatox.2023.106669.
15. Boukadida, K.; Mlouka, R.; Abelouah, M.R.; Chelly, S.; Romdhani, I.; Conti, G.O.; Ferrante, M.; Cammarata, M.; Parisi, M.G.; AitAlla, A.; et al. Unraveling the Interplay between Environmental Microplastics and Salinity Stress on *Mytilus galloprovincialis* Larval Development: A Holistic Exploration. *Sci. Total Environ.* **2024**, *927*, 172177, doi:10.1016/j.scitotenv.2024.172177.
16. Choi, J.S.; Kim, K.; Park, K.; Park, J.-W. Long-Term Exposure of the Mediterranean Mussels, *Mytilus galloprovincialis* to Polyethylene Terephthalate Microfibers: Implication for Reproductive and Neurotoxic Effects. *Chemosphere* **2022**, *299*, 134317, doi:10.1016/j.chemosphere.2022.134317.
17. Fernández, B.; Vidal-Liñán, L.; Bellas, J.; Campillo, J.A.; Chaves-Pozo, E.; Albentosa, M. The Particle Effect: Comparative Toxicity of Chlorpyrifos in Combination with Microplastics and Phytoplankton Particles in Mussel. *Aquat. Toxicol.* **2024**, *275*, 107053, doi:10.1016/j.aquatox.2024.107053.
18. Tuncelli, G.; Can Tuncelli, I.; Dagsuyu, E.; Turkyilmaz, I.B.; Yanardag, R.; Erkan, N. The Effect of Different Types of Microplastic and Acute Cadmium Exposure on the *Mytilus galloprovincialis* (Lamarck, 1819). *Sci. Total Environ.* **2024**, *936*, 173505, doi:10.1016/j.scitotenv.2024.173505.
19. Vilke, J.M.; Fonseca, T.G.; Alkimin, G.D.; Gonçalves, J.M.; Edo, C.; Errico, G.D.; Seilitz, F.S.; Rotander, A.; Benedetti, M.; Regoli, F.; et al. Looking beyond the Obvious: The Ecotoxicological Impact of the Leachate from Fishing Nets and Cables in the Marine Mussel *Mytilus galloprovincialis*. *J. Hazard. Mater.* **2024**, *473*, 134479, doi:10.1016/j.jhazmat.2024.134479.
20. Huang, W.; Wang, X.; Chen, D.; Xu, E.G.; Luo, X.; Zeng, J.; Huan, T.; Li, L.; Wang, Y. Toxicity Mechanisms of Polystyrene Microplastics in Marine Mussels Revealed by High-Coverage Quantitative Metabolomics Using Chemical Isotope Labeling Liquid Chromatography Mass Spectrometry. *J. Hazard. Mater.* **2021**, *417*, 126003, doi:10.1016/j.jhazmat.2021.126003.
21. Qi, P.; Qiu, L.; Feng, D.; Gu, Z.; Guo, B.; Yan, X. Distinguish the Toxic Differentiations between Acute Exposure of Micro- and Nano-Plastics on Bivalves: An Integrated Study Based on Transcriptomic Sequencing. *Aquat. Toxicol.* **2023**, *254*, 106367, doi:10.1016/j.aquatox.2022.106367.

22. Zhong, Z.; Huang, W.; Yin, Y.; Wang, S.; Chen, L.; Chen, Z.; Wang, J.; Li, L.; Khalid, M.; Hu, M.; et al. Tris(1-Chloro-2-Propyl) Phosphate Enhances the Adverse Effects of Biodegradable Polylactic Acid Microplastics on the Mussel *Mytilus coruscus*. *Environ. Pollut.* **2024**, *359*, 124741, doi:10.1016/j.envpol.2024.124741.
23. Zhang, Z.-M.; Liu, H.; Zuo, H.-L.; Wang, Y.-N.; Sun, A.-L.; Chen, J.; Shi, X.-Z. Unraveling the Toxic Trio: Combined Effects of Thifluzamide, Enrofloxacin, and Microplastics on *Mytilus coruscus*. *J. Hazard. Mater.* **2025**, *494*, 138441, doi:10.1016/j.jhazmat.2025.138441.
24. Eliso, M.C.; Billè, B.; De Marco, G.; Pulvirenti, E.; Dal Bello, F.; Rapisarda, P.; Pereira, P.; Galati, M.; Oliveri Conti, G.; Ferrante, M.; et al. Embryotoxicity of Polystyrene Microplastics, Alone and Conjugated with Bisphenol A, in the Black Sea Urchin *Arbacia lixula*: A Multi-Biomarker Approach. *J. Hazard. Mater.* **2025**, *499*, 140139, doi:10.1016/j.jhazmat.2025.140139.
25. Lin, D.; Cen, Z.; Zhang, C.; Lin, X.; Liang, T.; Xu, Y.; Zheng, L.; Qiao, Q.; Huang, L.; Xiong, K. Triclosan-Loaded Aged Microplastics Exacerbate Oxidative Stress and Neurotoxicity in *Xenopus tropicalis* Tadpoles via Increased Bioaccumulation. *Sci. Total Environ.* **2024**, *935*, 173457, doi:10.1016/j.scitotenv.2024.173457.
26. Zhang, W.; Zhang, W.; Teng, M.; Xu, J.; Wang, J.; Yang, J.; Liu, Y. The Effect and Mechanism of Variable Particle Size Microplastics and Levofloxacin on the Neurotoxicity of *Rana nigromaculata* Based on the Microorganism-Intestine-Brain Axis. *J. Environ. Manage.* **2024**, *354*, 120329, doi:10.1016/j.jenvman.2024.120329.
27. Sökmen, T.Ö.; Sulukan, E.; Türkoğlu, M.; Baran, A.; Özkaraca, M.; Ceyhun, S.B. Polystyrene Nanoplastics (20 nm) Are Able to Bioaccumulate and Cause Oxidative DNA Damages in the Brain Tissue of Zebrafish Embryo (*Danio rerio*). *NeuroToxicology* **2020**, *77*, 51–59, doi:10.1016/j.neuro.2019.12.010.
28. Liu, Y.; Wang, Y.; Li, N.; Jiang, S. Avobenzone and Nanoplastics Affect the Development of Zebrafish Nervous System and Retinal System and Inhibit Their Locomotor Behavior. *Sci. Total Environ.* **2022**, *806*, 150681, doi:10.1016/j.scitotenv.2021.150681.
29. Qualhato, G.; Cirqueira Dias, F.; Rocha, T.L. Hazardous Effects of Plastic Microfibres from Facial Masks to Aquatic Animal Health: Insights from Zebrafish Model. *Sci. Total Environ.* **2024**, *951*, 175555, doi:10.1016/j.scitotenv.2024.175555.
30. Kankaynar, M.; Sulukan, E.; Yildirim, S.; Şenol, O.; Atakay, M.; Baran, A.; Kiliclioglu, M.; Bolat, İ.; Yildiz, E.; Ceyhun, H.A.; et al. Unseen Threats: How Nanoplastics Trigger Anxiety and Depression-like Behaviors in Zebrafish (*Danio rerio*). *Environ. Pollut.* **2025**, *386*, 127229, doi:10.1016/j.envpol.2025.127229.
31. Orozco-Hernández, J.M.; Hernández-Varela, J.D.; Gómez-Oliván, L.M.; Chanona-Pérez, J.J.; Hernández-Díaz, M.; Juan-Reyes, N.S.; Rosales-Pérez, K.E.; Juan-Reyes, S.S. Toxic Interactions between Fluoxetine and Microplastics in Zebrafish Embryonic Development. *Sci. Total Environ.* **2025**, *970*, 179040, doi:10.1016/j.scitotenv.2025.179040.
32. Song, J.; Pu, Q.; Chen, C.; Liu, X.; Zhang, X.; Wang, Z.; Yan, J.; Wang, X.; Wang, H.; Qian, Q. Neurological Outcomes of Joint Exposure to Polystyrene Micro/Nanospheres and Silver Nanoparticles in Zebrafish. *Environ. Health Perspect.* **2025**, *133*, 057007, doi:10.1289/EHP14873.
33. Li, J.; Chen, Y.; Chen, Y.; Xie, H.; Wu, G.; Zhang, Y.; Wu, K. Polystyrene Microplastics and Nanoplastics Induce Neurotoxicity in Zebrafish via Oxidative Stress and Neurotransmitter Disruption. *Comp. Biochem. Physiol. Part C Toxicol. Pharmacol.* **2026**, *300*, 110397, doi:10.1016/j.cbpc.2025.110397.
34. Wang, Q.; Chen, G.; Tian, L.; Kong, C.; Gao, D.; Chen, Y.; Junaid, M.; Wang, J. Neuro- and Hepato-Toxicity of Polystyrene Nanoplastics and Polybrominated Diphenyl Ethers on Early Life Stages of Zebrafish. *Sci. Total Environ.* **2023**, *857*, 159567, doi:10.1016/j.scitotenv.2022.159567.

35. Sun, Y.; Ding, P.; Zhang, J.; Sun, K.; Li, X.; Ge, Q.; Dang, Y.; Yu, Y.; Hu, G. Combined Neurotoxicity of Aged Microplastics and Thiamethoxam in the Early Developmental Stages of Zebrafish (*Danio rerio*). *Environ. Pollut.* **2024**, *348*, 123853, doi:10.1016/j.envpol.2024.123853.
36. Liu, Y.; Tang, Q.-P.; Zuo, S.-J.; Ding, Y.; Guo, F.-Y.; Zhang, B.-F.; Zhou, Q.-H.; Xie, D.; Pei, D.-S. Synergistic Neurotoxicity of Polystyrene Nanoparticles and MEHP in Zebrafish (*Danio rerio*). *Environ. Pollut.* **2025**, *382*, 126765, doi:10.1016/j.envpol.2025.126765.
37. Oger, M.J.L.; Bernay, B.; Tessier, E.; Amouroux, D.; Kestemont, P.; Cornet, V. The Trojan Horse Effect of Nanoplastics Exacerbates Methylmercury-Induced Neurotoxicity during Zebrafish Development. *Environ. Pollut.* **2025**, *384*, 126966, doi:10.1016/j.envpol.2025.126966.
38. Kazemi, S.; Hanachi, P.; Zivary, S.; Kasmaie, A.; Walker, T.R.; Goshtasbi, H. Combined Effects of Polyethylene Terephthalate and Abamectin on Enzymatic Activity and Histopathology Response in Juvenile Zebrafish (*Danio rerio*). *Environ. Sci. Pollut. Res.* **2024**, *31*, 43987–43995, doi:10.1007/s11356-024-33981-9.
39. Wu, W.; Li, R.; Zhang, Z.; Liu, G.; Sun, Y.; Wang, C. The Exploration of Chronic Combined Toxic Mechanisms of Environmental PFOA and Polyethylene Micro/Nanoplastics on Adult Zebrafish (*Danio rerio*), Using Aquatic Microcosm Systems. *Aquat. Toxicol.* **2025**, *287*, 107534, doi:10.1016/j.aquatox.2025.107534.
40. Hanslik, L.; Huppertsberg, S.; Kämmer, N.; Knepper, T.P.; Braunbeck, T. Rethinking the Relevance of Microplastics as Vector for Anthropogenic Contaminants: Adsorption of Toxicants to Microplastics during Exposure in a Highly Polluted Stream - Analytical Quantification and Assessment of Toxic Effects in Zebrafish (*Danio rerio*). *Sci. Total Environ.* **2022**, *816*, 151640, doi:10.1016/j.scitotenv.2021.151640.
41. Sarasamma, S.; Audira, G.; Siregar, P.; Malhotra, N.; Lai, Y.-H.; Liang, S.-T.; Chen, J.-R.; Chen, K.H.-C.; Hsiao, C.-D. Nanoplastics Cause Neurobehavioral Impairments, Reproductive and Oxidative Damages, and Biomarker Responses in Zebrafish: Throwing up Alarms of Wide Spread Health Risk of Exposure. *Int. J. Mol. Sci.* **2020**, *21*, 1410, doi:10.3390/ijms21041410.
42. Santos, D.; Luzio, A.; Félix, L.; Bellas, J.; Monteiro, S.M. Oxidative Stress, Apoptosis and Serotonergic System Changes in Zebrafish (*Danio rerio*) Gills after Long-Term Exposure to Microplastics and Copper. *Comp. Biochem. Physiol. Part C Toxicol. Pharmacol.* **2022**, *258*, 109363, doi:10.1016/j.cbpc.2022.109363.
43. Zhang, C.; Li, Y.; Yu, H.; Li, T.; Ye, L.; Zhang, X.; Wang, C.; Li, P.; Ji, H.; Gao, Q.; et al. Co-Exposure of Nanoplastics and Arsenic Causes Neurotoxicity in Zebrafish (*Danio rerio*) through Disrupting Homeostasis of Microbiota–Intestine–Brain Axis. *Sci. Total Environ.* **2024**, *912*, 169430, doi:10.1016/j.scitotenv.2023.169430.
44. Su, M.; Gu, D.; Liang, L.; Zhou, Z.; Zhu, C.; Qi, J.; Wu, P.; Xu, T.; Jiang, Z. Size-Dependent and Tissue Specific Accumulation of Polystyrene Microplastics and Nanoplastics in Zebrafish. *Aquat. Toxicol.* **2026**, *291*, 107678, doi:10.1016/j.aquatox.2025.107678.
45. Wang, X.; Li, R.; Cheng, B.; Sun, Y.; Yao, X.; Wang, C. Combined Toxicity of Polyethylene Micro/Nanoplastics and PFOA in Zebrafish (*Danio rerio*): Impacts on Antioxidant, Neurotransmission, and Gut Microbiota. *Environ. Toxicol. Pharmacol.* **2026**, *121*, 104907, doi:10.1016/j.etap.2025.104907.
46. Zitouni, N.; Cappello, T.; Missawi, O.; Boughattas, I.; De Marco, G.; Belbekhouche, S.; Mokni, M.; Alphonse, V.; Guerbej, H.; Bousserrhine, N.; et al. Metabolomic Disorders Unveil Hepatotoxicity of Environmental Microplastics in Wild Fish *Serranus scriba* (Linnaeus 1758). *Sci. Total Environ.* **2022**, *838*, 155872, doi:10.1016/j.scitotenv.2022.155872.

47. Martins, A.; Barboza, L.G.; Vieira, L.R.; Botelho, M.J.; Vale, C.; Guilhermino, L. Relations between Microplastic Contamination and Stress Biomarkers under Two Seasonal Conditions in Wild Carps, Mullets and Flounders. *Mar. Environ. Res.* **2025**, *204*, 106925, doi:10.1016/j.marenvres.2024.106925.
48. Rasta, M.; Kakakhel, M.A.; Taleshi, M.S.; Lashkaryan, N.S.; Manke, J.; Liu, L.; Soomro, S.; Shi, X. Interactive Effects of Hydrodynamics and Microplastics on Bioaccumulation, Histopathological Alterations, Biomarker Responses, and Gene Expression in Grass Carp Brain. *Ecotoxicol. Environ. Saf.* **2025**, *302*, 118596, doi:10.1016/j.ecoenv.2025.118596.
49. Zhang, P.; Lu, G.; Zhang, L.; Yan, Z.; Zhang, J.; Ding, K. Unraveling the Mechanism of Brain Damage in *Carassius auratus* by Polypropylene Microplastics and Oxytetracycline via the Brain-Gut-Microbiota Axis. *J. Environ. Manage.* **2025**, *392*, 126711, doi:10.1016/j.jenvman.2025.126711.
50. Zheng, Q.; Zheng, Y.; Junaid, M.; Zeng, M.; Liao, H.; Li, Y.; Zhao, Y.; Huang, Q.; Wang, J. Biochar Alleviates Nanoplastics and Bisphenol A Mediated Immunological, Neurological and Gut Microbial Toxicity in Channel Catfish *Ictalurus punctatus*. *Chemosphere* **2025**, *378*, 144422, doi:10.1016/j.chemosphere.2025.144422.
51. Iheanacho, S.C.; Odo, G.E. Neurotoxicity, Oxidative Stress Biomarkers and Haematological Responses in African Catfish (*Clarias gariepinus*) Exposed to Polyvinyl Chloride Microparticles. *Comp. Biochem. Physiol. Part C Toxicol. Pharmacol.* **2020**, *232*, 108741, doi:10.1016/j.cbpc.2020.108741.
52. Usman, S.; Abdull Razis, A.F.; Shaari, K.; Amal, M.N.A.; Saad, M.Z.; Mat Isa, N.; Nazarudin, M.F. Polystyrene Microplastics Exposure: An Insight into Multiple Organ Histological Alterations, Oxidative Stress and Neurotoxicity in Javanese Medaka Fish (*Oryzias javanicus* Bleeker, 1854). *Int. J. Environ. Res. Public. Health* **2021**, *18*, 9449, doi:10.3390/ijerph18189449.
53. Alanazi, M.Q.; Virk, P.; Alterary, S.S.; Awad, M.; Ahmad, Z.; Albadri, A.M.; Ortashi, KalidM.; Ahmed, M.M.A.; Ali Yousef, M.I.; Elobeid, M.; et al. Effect of Potential Microplastics in Sewage Effluent on Nile Tilapia and Photocatalytic Remediation with Zinc Oxide Nanoparticles. *Environ. Pollut.* **2023**, *332*, 121946, doi:10.1016/j.envpol.2023.121946.
54. Ding, J.; Huang, Y.; Liu, S.; Zhang, S.; Zou, H.; Wang, Z.; Zhu, W.; Geng, J. Toxicological Effects of Nano- and Micro-Polystyrene Plastics on Red Tilapia: Are Larger Plastic Particles More Harmless? *J. Hazard. Mater.* **2020**, *396*, 122693, doi:10.1016/j.jhazmat.2020.122693.
55. Huang, Y.; Ding, J.; Zhang, G.; Liu, S.; Zou, H.; Wang, Z.; Zhu, W.; Geng, J. Interactive Effects of Microplastics and Selected Pharmaceuticals on Red Tilapia: Role of Microplastic Aging. *Sci. Total Environ.* **2021**, *752*, 142256, doi:10.1016/j.scitotenv.2020.142256.
56. Bakhasha, J.; Saxena, V.; Arya, N.; Kumar, P.; Srivastava, A.; Yadav, K.K.; Tomar, S.; Mishra, S.; Banaee, M.; Faggio, C.; et al. Neurotoxic Synergy of Copper and PVC Microplastics Triggers Apoptosis via the BDNF/miR132/FOXO3a Pathway for the First Time in Fish Brain. *Comp. Biochem. Physiol. Part C Toxicol. Pharmacol.* **2025**, *297*, 110265, doi:10.1016/j.cbpc.2025.110265.
57. Lourenço, S.C.; Aleluia, A.A.M.R.A.; Barboza, L.G.A.; Otero, X.L.; Cunha, S.C.; Fernandes, J.O.; Guilhermino, L. Microplastic Contamination and Biological Alterations in Atlantic Wild Fish Populations, and Human Health Risks Associated to Fillet Consumption. *Mar. Environ. Res.* **2025**, *208*, 107139, doi:10.1016/j.marenvres.2025.107139.
58. Gutierrez-Rodriguez, A.; Nuñez-Moyano, C.; Rivas-Iglesias, L.; Acle, S.; Royo-Martin, L.J.; Garcia-Vazquez, E.; Machado-Schiaffino, G. Molecular Responses to Pollution Stress in Glass Eels (*Anguilla anguilla*): Gene Expression Changes Associated with Varying Contamination Levels and Temperature across Estuaries. *Aquat. Toxicol.* **2026**, *290*, 107623, doi:10.1016/j.aquatox.2025.107623.

59. Chagas, T.Q.; Freitas, Í.N.; Montalvão, M.F.; Nobrega, R.H.; Machado, M.R.F.; Charlie-Silva, I.; Araújo, A.P.D.C.; Guimarães, A.T.B.; Alvarez, T.G.D.S.; Malafaia, G. Multiple Endpoints of Polylactic Acid Biomicroplastic Toxicity in Adult Zebrafish (*Danio rerio*). *Chemosphere* **2021**, *277*, 130279, doi:10.1016/j.chemosphere.2021.130279.
60. Santos, D.; Luzio, A.; Bellas, J.; Monteiro, S.M. Microplastics- and Copper-Induced Changes in Neurogenesis and DNA Methyltransferases in the Early Life Stages of Zebrafish. *Chem. Biol. Interact.* **2022**, *363*, 110021, doi:10.1016/j.cbi.2022.110021.
61. Teng, M.; Zhao, X.; Wu, F.; Wang, C.; Wang, C.; White, J.C.; Zhao, W.; Zhou, L.; Yan, S.; Tian, S. Charge-Specific Adverse Effects of Polystyrene Nanoplastics on Zebrafish (*Danio rerio*) Development and Behavior. *Environ. Int.* **2022**, *163*, 107154, doi:10.1016/j.envint.2022.107154.
62. Lin, L.-Y.; Kantha, P.; Horng, J.-L. Toxic Effects of Polystyrene Nanoparticles on the Development, Escape Locomotion, and Lateral-Line Sensory Function of Zebrafish Embryos. *Comp. Biochem. Physiol. Part C Toxicol. Pharmacol.* **2023**, *272*, 109701, doi:10.1016/j.cbpc.2023.109701.
63. Torres-Ruiz, M.; De Alba González, M.; Morales, M.; Martin-Folgar, R.; González, M.C.; Cañas-Portilla, A.I.; De La Vieja, A. Neurotoxicity and Endocrine Disruption Caused by Polystyrene Nanoparticles in Zebrafish Embryo. *Sci. Total Environ.* **2023**, *874*, 162406, doi:10.1016/j.scitotenv.2023.162406.
64. Xiang, C.; Chen, H.; Liu, X.; Dang, Y.; Li, X.; Yu, Y.; Li, B.; Li, X.; Sun, Y.; Ding, P.; et al. UV-Aged Microplastics Induces Neurotoxicity by Affecting the Neurotransmission in Larval Zebrafish. *Chemosphere* **2023**, *324*, 138252, doi:10.1016/j.chemosphere.2023.138252.
65. Yang, H.; Kong, L.; Chen, Z.; Wu, J. Effect of Functional Groups of Polystyrene Nanoplastics on the Neurodevelopmental Toxicity of Acrylamide in the Early Life Stage of Zebrafish. *Aquat. Toxicol.* **2025**, *278*, 107177, doi:10.1016/j.aquatox.2024.107177.
66. Saputra, F.; Tsao, Y.-T.; Pramata, A.D.; Soegianto, A.; Hu, S.-Y. Polystyrene Nanoplastics Act as Endocrine Disruptors Altering Neurotransmitter Levels and Locomotor Activity via Estrogen Receptor during Early Zebrafish Development. *Aquat. Toxicol.* **2025**, *286*, 107468, doi:10.1016/j.aquatox.2025.107468.
67. Varshney, S.; Hegstad-Pettersen, M.M.; Siriyappagouder, P.; Olsvik, P.A. Enhanced Neurotoxic Effect of PCB-153 When Co-Exposed with Polystyrene Nanoplastics in Zebrafish Larvae. *Chemosphere* **2024**, *355*, 141783, doi:10.1016/j.chemosphere.2024.141783.
68. Teng, M.; Li, Y.; Zhao, L.; White, J.C.; Sun, J.; Zhang, Z.; Chen, L.; Zhu, J.; Wu, F. Life Cycle Exposure to Differentially Charged Polystyrene Nanoplastics Leads to Gender-Specific Particle Accumulation and Neurotoxicity in Zebrafish (*Danio rerio*). *Environ. Int.* **2025**, *198*, 109441, doi:10.1016/j.envint.2025.109441.
69. Yang, B.; Han, Y.; Hu, S.; Xie, X.; Zhu, X.; Yuan, L. Polystyrene Microplastics Induce Depression-like Behavior in Zebrafish via Neuroinflammation and Circadian Rhythm Disruption. *Sci. Total Environ.* **2025**, *959*, 178085, doi:10.1016/j.scitotenv.2024.178085.
70. Shi, W.; Sun, S.; Han, Y.; Tang, Y.; Zhou, W.; Du, X.; Liu, G. Microplastics Impair Olfactory-Mediated Behaviors of Goldfish *Carassius auratus*. *J. Hazard. Mater.* **2021**, *409*, 125016, doi:10.1016/j.jhazmat.2020.125016.
71. Gonçalves, J.M.; Benedetti, M.; d'Errico, G.; Regoli, F.; Bebianno, M.J. Polystyrene Nanoplastics in the Marine Mussel *Mytilus galloprovincialis*. *Environ. Pollut.* **2023**, *333*, 122104, doi:10.1016/j.envpol.2023.122104.
72. Melki, S.; Ferrari, E.; Ahmed, R.B.; Spagnuolo, A.; Corsi, I. Single but Not Combined In Vitro Exposure to Bisphenol A and Nanoplastics Affects the Cholinergic Function of the Ascidian *Ciona robusta*. *J. Xenobiotics* **2024**, *14*, 1930–1940, doi:10.3390/jox14040103.
73. Ding, P.; Han, Y.; Sun, Y.; Chen, X.; Ge, Q.; Huang, W.; Zhang, L.; Li, A.J.; Hu, G.; Yu, Y. Synergistic Neurotoxicity of Clothianidin and Photoaged Microplastics in Zebrafish:

Implications for Neuroendocrine Disruption. *Environ. Pollut.* **2025**, *368*, 125797, doi:10.1016/j.envpol.2025.125797.
74. De Oliveira, J.P.J.; Estrela, F.N.; Rodrigues, A.S.D.L.; Guimarães, A.T.B.; Rocha, T.L.; Malafaia, G. Behavioral and Biochemical Consequences of *Danio rerio* Larvae Exposure to Polylactic Acid Bioplastic. *J. Hazard. Mater.* **2021**, *404*, 124152, doi:10.1016/j.jhazmat.2020.124152.
75. Santos, D.; Luzio, A.; Matos, C.; Bellas, J.; Monteiro, S.M.; Félix, L. Microplastics Alone or Co-Exposed with Copper Induce Neurotoxicity and Behavioral Alterations on Zebrafish Larvae after a Subchronic Exposure. *Aquat. Toxicol.* **2021**, *235*, 105814, doi:10.1016/j.aquatox.2021.105814.
76. Xue, Y.-H.; Feng, L.-S.; Xu, Z.-Y.; Zhao, F.-Y.; Wen, X.-L.; Jin, T.; Sun, Z.-X. The Time-Dependent Variations of Zebrafish Intestine and Gill after Polyethylene Microplastics Exposure. *Ecotoxicology* **2021**, *30*, 1997–2010, doi:10.1007/s10646-021-02469-4.
77. Choi, J.-H.; Lee, J.-H.; Jo, A.-H.; Choi, Y.J.; Choi, C.Y.; Kang, J.-C.; Kim, J.-H. Microplastic Polyamide Toxicity: Neurotoxicity, Stress Indicators and Immune Responses in Crucian Carp, *Carassius carassius*. *Ecotoxicol. Environ. Saf.* **2023**, *265*, 115469, doi:10.1016/j.ecoenv.2023.115469.
78. Roda, J.F.B.; Lauer, M.M.; Risso, W.E.; Bueno Dos Reis Martinez, C. Microplastics and Copper Effects on the Neotropical Teleost *Prochilodus lineatus*: Is There Any Interaction? *Comp. Biochem. Physiol. A. Mol. Integr. Physiol.* **2020**, *242*, 110659, doi:10.1016/j.cbpa.2020.110659.
79. Martin-Folgar, R.; Torres-Ruiz, M.; De Alba, M.; Cañas-Portilla, A.I.; González, M.C.; Morales, M. Molecular Effects of Polystyrene Nanoplastics Toxicity in Zebrafish Embryos (*Danio rerio*). *Chemosphere* **2023**, *312*, 137077, doi:10.1016/j.chemosphere.2022.137077.
80. Levesque, B.; Hrapovic, S.; Berrué, F.; Vogt, A.; Ellis, L.D.; Hermabessiere, L. Evaluation of Phenotypic and Behavioral Toxicity of Micro- and Nano-Plastic Polystyrene Particles in Larval Zebrafish (*Danio rerio*). *Toxicol. Sci.* **2025**, *205*, 154–165, doi:10.1093/toxsci/kfaf015.
